# Structural insights into hybrid immiscible blends of metal–organic framework and sodium ultraphosphate glasses†

**DOI:** 10.1039/d3sc02305b

**Published:** 2023-09-12

**Authors:** Ashleigh M. Chester, Celia Castillo-Blas, Roman Sajzew, Bruno P. Rodrigues, Ruben Mas-Balleste, Alicia Moya, Jessica E. Snelson, Sean M. Collins, Adam F. Sapnik, Georgina P. Robertson, Daniel J. M. Irving, Lothar Wondraczek, David A. Keen, Thomas D. Bennett

**Affiliations:** a Department of Materials Science and Metallurgy, University of Cambridge Cambridge CB3 0FS UK tdb35@cam.ac.uk; b Otto Schott Institute Materials Research, University of Jena Fraunhoferstrasse 6 07743 Jena Germany; c Department of Inorganic Chemistry, Universidad Autónoma de Madrid 28049 Madrid Spain; d Institute for Advanced Research in Chemical Sciences (IAdChem), Universidad Autónoma de Madrid 28049 Madrid Spain; e School of Chemical and Process Engineering, School of Chemistry, Bragg Centre for Materials Research, University of Leeds Woodhouse Lane LS2 9JT UK; f Diamond Light Source Ltd Diamond House, Harwell Campus, Didcot, Oxfordshire OX11 0DE UK; g ISIS Facility, Rutherford Appleton Laboratory Harwell Campus, Didcot, Oxfordshire OX11 0QX UK

## Abstract

Recently, increased attention has been focused on amorphous metal–organic frameworks (MOFs) and, more specifically, MOF glasses, the first new glass category discovered since the 1970s. In this work, we explore the fabrication of a compositional series of hybrid blends, the first example of blending a MOF and inorganic glass. We combine ZIF-62(Zn) glass and an inorganic glass, 30Na_2_O–70P_2_O_5_, to combine the chemical versatility of the MOF glass with the mechanical properties of the inorganic glass. We investigate the interfacial interactions between the two components using pair distribution function analysis and solid state NMR spectroscopy, and suggest potential interactions between the two phases. Thermal analysis of the blend samples indicated that they were less thermally stable than the starting materials and had a *T*_g_ shifted relative to the pristine materials. Annular dark field scanning transmission electron microscopy tomography, X-ray energy dispersive spectroscopy (EDS), nanoindentation and ^31^P NMR all indicated close mixing of the two phases, suggesting the formation of immiscible blends.

## Abstract 2

The characterisation of hybrid blends comprising a metal–organic framework (MOF) glass and a phosphate glass is explored. The mechanical and thermal properties of the blends are studied and interfacial interactions between the phases are analysed.

## Graphical abstract

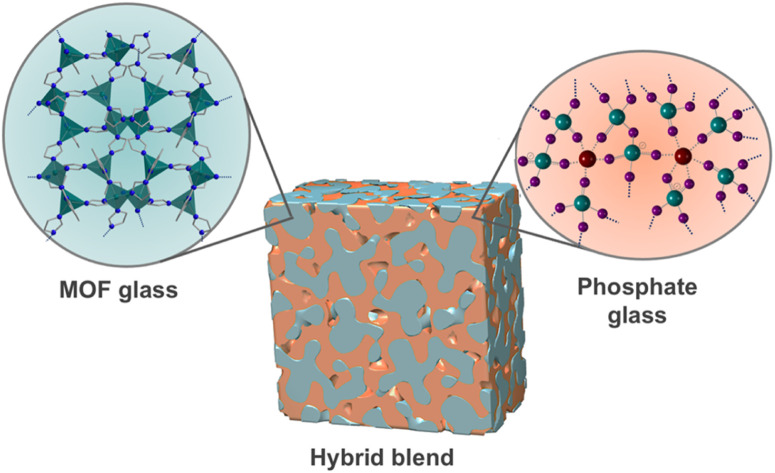

## Introduction

In recent years, rapidly growing interest in metal–organic frameworks (MOFs) has emerged on account of their chemical tuneability, versatile structures and unique physical properties. MOFs comprise metal ions or clusters known as secondary building units (SBUs) and multidentate organic linkers.^1–3^ Various potential applications of MOFs have been suggested; these include drug delivery,^4^ heterogenous catalysis,^5^ water harvesting,^6^ gas storage and separation,^7^ and optical sensing.^8^

In addition to their porous architectures and high internal surface areas, an interesting phenomenon has emerged whereby several MOFs can be melt-quenched to form a MOF glass. A glass is considered an amorphous material that exhibits a transition from a brittle solid to a viscoelastic state over a specific temperature range, characterised by the glass transition temperature (*T*_g_).^9^

A large proportion of known MOF glasses are melt-quenched from crystalline zeolitic imidazolate frameworks (ZIFs), a subgroup of MOFs, which consist of tetrahedrally-coordinated metal ions (*e.g.* Zn^2+^, Co^2+^) and imidazolate or imidazolate-derived linkers.^10,11^

**Fig. 1:**
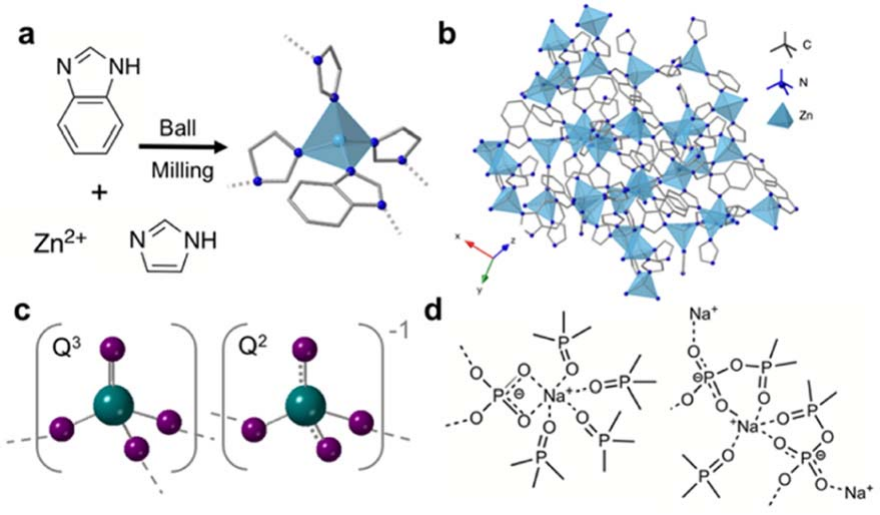
(a) Synthesis route to ZIF-62, (b) crystal structure of ZIF-62 where dark blue, grey and light blue represent nitrogen, carbon and zinc respectively, (c) polyhedra contained in phosphate glasses with 70% P_2_O_5_ (oxygen-purple, phosphorous-green), dashed lines indicate delocalisation of the negative charge on the non-bridging oxygen atoms and (d) structural motifs present upon the addition of sodium oxide to ultraphosphate glasses.

A crystalline ZIF that has excellent glass forming ability is ZIF-62, [Zn(Im)_1.75_(bIm)_0.25_] where Im = imidazolate (C_3_H_3_N_2_^−^) and bIm = benzimidazolate (C_7_H_5_N_2_^−^) (Fig. 1a and b). Suggested applications of ZIF-62 glass (a_g_ZIF-62) include energy storage,^11^ catalysis^12^ and lithium-ion batteries.^13^ Additionally, the use of a_g_ZIF-62 as gas separation membranes has also been studied because of its intrinsic microporosity.^14^ Glassy membranes are particularly interesting as they avoid issues arising from defects and grain boundaries associated with membranes composed of polycrystalline materials.^11,15,16^

Several studies have focused on combining a_g_ZIF-62 with other materials, such as crystalline MOFs,^17^ carbon black,^13^ lead halide perovskites^18^ and organic materials to form composite membranes.^19,20^

Currently, phosphate glasses are of interest to a multitude of industries as laser hosts,^21^ biomaterials,^22^ proton conductors,^23^ and in nuclear waste immobilisation and remediation.^24^ Structurally, phosphate glasses are based on the network former P_2_O_5_ with PO_4_ building blocks interconnected *via* bridging oxygen atoms (Fig. 1c).^25,26^ These polyhedra can be described by Q^*n*^ notation, where *n* is the number of bridging oxygen atoms per tetrahedra. Unmodified phosphate glasses comprise Q^3^ groups which contain a phosphorous atom with one P=O and connected to three bridging oxygen atoms. This intrinsic non-bridging oxygen (NBO) is responsible for the lower melting temperatures (*T*_m_), *T*_g_s and higher melt fragilities of phosphate glasses when compared to similar silicate and germanate glasses. For binary ultraphosphate glasses, such as sodium ultraphosphate glasses *x*Na_2_O(1 − *x*)-P_2_O_5_, where 0 ⩽ *x* ⩽ 0.5, Q^2^ and Q^3^ groups are the dominant species.^27^ Q^3^ species, described above, contain a P=O bond, whereas Q^2^ species contain a phosphorous atom connected to two bridging atoms. Upon the addition of modifiers such as alkali and alkali earth metal oxides to vitreous P_2_O_5_ (*i.e.*, 100% P_2_O_5_ glass), the P–O–P bonds throughout the glass are disrupted *via* the conversion of these bridging oxygen species to NBOs.^28^ The subsequent reduction in network connectivity decreases the *T*_g_ of the modified glass. When sodium oxide is introduced to the phosphate network, various structural motifs (Fig. 1d) have been identified, in which sodium ions form ionic bonds with the oxygen atoms in the Q^2^ and Q^3^ units.^29^

A potential advantage of blending two glasses is avoiding the drawbacks associated with the constituent parent materials. Blending is well-established in polymer mixing in the plastics industry,^30,31^ where a blend can be considered a macroscopically homogenous mixture of two or more different species.^32^ MOF glasses exhibit elastic moduli in between those of inorganic glasses (brittle yet scratch resistant) and organic polymers (ductile yet scratch prone).^33,34^ In the case of a_g_ZIF-62 however, workability (*i.e.*, the degree to which a material can be shaped without crack formation) is hampered by a high viscosity in the molten phase, which makes it hard to cast into different morphologies.^35^ As such, producing bulk, bubble-free a_g_ZIF-62 remains a substantial challenge, owing to the high viscosity (*η* = 10^5.1^ Pa s)^36^ at the *T*_m_ (∼437 °C) of crystalline ZIF-62.^36^ On the other hand, ultraphosphate glasses are hygroscopic and combining them with a hydrophobic glass could improve their stability.

Here, we report the synthesis of a compositional series of a_g_ZIF-62 and an ultraphosphate glass, 30Na_2_O–70P_2_O_5_ to combine the chemical tuneability and porosity of MOF glasses with the mechanical properties of phosphate glasses. We term the resulting products immiscible blends, where immiscibility refers to the inability of a mixture to form a single phase.^32^

## Experimental

### Materials

Zinc oxide nanopowder (ZnO), zinc acetate dihydrate (Zn(OAc)_2_·2H_2_O, ⩾98%) and imidazole (⩾99.5%) were purchased from Sigma Aldrich. Benzimidazole (99%) was purchased from Alfa Aesar. *N*,*N*-Dimethylformamide (DMF) (99.5%) and dichloromethane (DCM) stabilised with amylene (99.8%) were purchased from Fischer Scientific. Dimethyl sulfoxide (DMSO-*d*_6_) (99.8 at% *D*, containing 0.03% (v/v) tetramethylsilane (TMS)) was purchased from VWR. All materials were used as received.

### ZIF-62 mechanosynthesis and glass synthesis

ZnO (<100 nm, 400.95 mg, 4.95 mmol), Zn(OAc)_2_·2H_2_O (10.975 mg, 0.05 mmol), imidazole (595 mg, 8.75 mmol), benzimidazole (147.5 mg, 1.25 mmol) and DMF (500 μL) were added to a 50 mL stainless steel grinding jar. Two 20 mm stainless steel grinding balls were added and the jar was sealed. The jar was shaken at 30 Hz for 30 minutes in a Retsch MM400 mixer mill. The powder was isolated by vacuum filtration and washed with fresh DMF (60 mL). The washed powder was placed in approximately 20 mL DCM for 24 hours for solvent exchange. After this period, the residual DCM was removed by vacuum filtration and the powder was placed in a vacuum oven under dynamic vacuum for three hours at 170 °C. To synthesise the a_g_ZIF-62, crystalline ZIF-62 was heated to 400 °C for one hour in a vacuum furnace and then left to cool to room temperature under vacuum.

### Synthesis of 30Na_2_O–70P_2_O_5_

(NH_4_)_2_HPO_4_ and Na_2_CO_3_ were weighed and mixed in the appropriate proportions. The 50 g batches were then melted at 900 °C for one hour in alumina crucibles and quenched to form homogeneous glassy samples. After quenching, the glass was annealed at 20 °C below *T*_g_ for 30 minutes and allowed to cool to room temperature.

### Blend synthesis

Powders of a_g_ZIF-62 and 30Na_2_O–70P_2_O_5_ were ball-milled in a Retsch MM400 mixer mill for five minutes at 20 Hz. The homogenised mixtures were pelletised by applying 0.74 GPa in a 13 mm dye in a pellet press. Pellets were placed between metal slabs to force the grains of the individual components into close contact. The pellets were then held at 400 °C for 10 minutes in a vacuum furnace and allowed to cool to room temperature under vacuum.

### Powder X-ray diffraction (PXRD)

Samples were measured on a Bruker D8 advanced diffractometer (Cu Kα radiation *λ* = 1.518 Å) equipped with a position sensitive LynxEye detector with Bragg–Brentano parafocusing geometry at room temperature. Samples were packed into 5 mm discs on low background silicon substrates. Pawley refinement of crystalline ZIF-62 was performed using the crystallographic information file of ZIF-62 and Topas academic software version 7.2.^37^

### Differential scanning calorimetry (DSC)

DSC measurements were taken using a NETSCH DSC 214 Polyma instrument, using an empty aluminium crucible as the reference. Background corrections were performed for all measurements using an empty aluminium pan on the same heating cycle. Approximately five mg of each sample was loaded into an aluminium crucible (30 μL) with a pierced lid under argon. Heating and cooling rates were 10 °C min^−1^. Glass transition temperatures (*T*_g_) were obtained from the second DSC upscan and corresponded to the mid-point of the change in gradient of the heat flow signal.

### Simultaneous DSC-TGA

TGA traces of all samples were recorded using a TA instruments Q-650 series DSC and the data analysed using TA Universal Analysis software. Approximately 5–10 mg of each sample was placed in open alumina crucibles and heated at 10 °C min^−1^ under argon.

### Thermomechanical analysis (TMA)

Small sections of pelletised sample were analysed using TA Instruments Q400 thermomechanical materials analyser (TMA), with an applied force of 10 mN under a nitrogen atmosphere. All samples were heated below the onset of thermal decomposition; details on the heating programs used can be found in the figure captions.

### ^1^H Nuclear magnetic resonance (NMR) spectroscopy

Approximately five mg of each sample was dissolved in a solution of DMSO-*d*_6_ (1.0 mL, 0.03% TMS) and D_2_O (0.2 mL, 35% DCl), before measurement at room temperature on a Bruker Advance III HD 500 MHz spectrometer at the Department of Chemistry, University of Cambridge. TMS was used as a standard and data processing and analysis was carried out using TopSpin software version 4.1.1.^38^

### Optical microscopy

A Leica MZ95 microscope and a Optika C–B10 camera with a 10 megapixels CMOS sensor was used to obtain optical images of all samples.

### Pycnometry

He pycnometric measurements were performed on a Micromeritics AccuPyc 1340 with a 1 cm^3^ insert. Approximately 100–200 mg of sample was used per measurement and 10 volume measurements were performed per sample.

### Scanning electron microscopy-X-ray energy dispersive spectroscopy (SEM-EDS)

Samples were mounted on an aluminium stub using carbon tape and sputter coated with gold to avoid sample charging. The samples were coated using a current of 40 mA for one minute. SEM analysis was performed at 10 keV using a FEI Nova Nano SEM 450, calibrated with a copper metal standard. Secondary electron (SE) and backscattered electron (BSE) imaging modes were used, details are in the figure captions. EDS mapping was performed at 15 keV.

### Scanning transmission electron microscopy (STEM) and STEM-EDS

Powder samples were prepared for electron microscopy by first suspending them in isopropyl alcohol (IPA) followed by sonication for 10 minutes. The suspension was then drop-cast onto copper-supported C-flat grids (Protochips). Annular dark field scanning transmission electron microscopy (ADF-STEM) tomography and X-ray energy dispersive spectroscopy (EDS) was carried out using an FEI Titan^3^ Themis 300 (ThermoFisher) at the University of Leeds, equipped with a Super-X (Bruker) four-detector EDS system and an ‘X-FEG’ high brightness electron source. The microscope was operated at 300 kV. ADF-STEM images were acquired across a tilt range of −75° to +65°, with a tilt angle increment of 2°. EDS maps were acquired at 0° tilt for both samples after the acquisition of the tilt-series to avoid degradation of the tilt-series data from any electron beam-induced changes to the sample (beam damage) from increased electron fluence required for EDS mapping. Details on image processing can be found in the ESI.†

### Elemental analysis

CHN combustion analysis was carried out using a CE440 Elemental Analyser, EAI Exeter Analytical Inc at the Department of Chemistry, University of Cambridge. Approximately 3–5 mg of each sample was used for each measurement.

### WDX spectroscopy

Glass samples were embedded in polyester resin, polished and coated with carbon. Chemical composition was determined with a JEOL JXA-8230 electron microprobe analyser at the Geology Institute of the University of Jena. For each sample measurements of Na, P and O, composition was determined at 30 different positions.

### Fourier-transformed infrared spectroscopy

Approximately five mg of each sample was mixed with KBr and pelletised for FTIR measurement on a Thermo Scientific Nicolet iS10 model FTIR spectrometer in transmission mode. A background scan was taken between all samples and the scans had a resolution of 2 cm^−1^.

### Raman spectroscopy

Raman investigation was performed using a confocal Raman microscope (Renishaw InVia) equipped with a suitable edge-filter for the elastically scattered intensities and a 50× LD objective lens using 785 nm laser excitation to reduce fluorescence. Prior to sample characterisation, the grating and detector were calibrated against a single crystal silicon reference sample. Spectra were collected in the range 100–1250 cm^−1^ with ∼1.2 cm^−1^ resolution. Each measurement consisted of up to 60 individual accumulations at ∼1 s accumulation time to maximise the signal to noise ratio without oversaturation of the detector.

### Pair distribution function

Samples were ground and loaded into borosilicate capillaries with an inner diameter of 0.78 mm and a height of 3.9 cm before sealing. Total scattering data for PDF analysis were collected at the I15-1 beamline at the Diamond Light Source, UK (experiment EE20038, *λ* = 0.189578 Å, 65.40 keV). Empty instrument (background) and empty capillary scans were run; all scans were collected over a ∼0.4 < *Q* < ∼26 Å^−1^ range. The raw data were processed using GudrunX^39–41^ and over a 0.5 < *Q* < ∼20 Å^−1^ range and were corrected for background, container, absorption, multiple and Compton scattering. Fourier transformation of the processed total scattering data yielded real space pair distribution function, *G*(*r*). The *D*(*r*) form is used to accentuate high-*r* correlations.^41^

### ^31^P NMR spectroscopy

^31^P MAS NMR spectra were collected at 161.97 MHz (magnetic field of 9.4 T) on a Bruker AV-400-WB with a 4 mm triple channel probe with ZrO rotors, Kel-F-plug at room temperature and 10 kHz slew rate. A single π/2 pulse of 60 kHz and spectral width of 100 kHz was used in the direct irradiation tests. The relaxation time was 40 seconds and samples were accumulated for 128 scans. In the CP-MAS tests a ^1^H excitation pulse of 3 μs, 3 ms contact time, 100 kHz spectral width, ppm 15 decoupling at 80 kHz were used, and samples accumulated with 512 scans were used. The relaxation time was 5 seconds. In both cases, (NH_4_)H_2_PO_4_ (ADP) at 0.81 ppm was used as secondary reference with respect to H_3_PO_4_ (85%) as the primary reference. The chemical shift resolution was ± 0.2 ppm.

### Nanoindentation

Surface mechanical properties were studied with a nanoindentation setup (G200, KLA Inc.) equipped with a Continuous Stiffness Measurement (CSM) module. The indentation modulus *E* was determined from indentation experiments with a three-sided Berkovich diamond tip (Synton-MDP Inc.). The instrument's frame compliance and area function of the indenter tip were calibrated before the first experiment on a fused silica reference glass (Corning Code 7980, Corning Inc.). On each sample up to 200 indentations with a depth limit of 1 μm were performed at a constant strain-rate of 0.05 s^−1^ and the analysis performed according to the procedure proposed by Oliver and Pharr.^42^

## Results and discussion

### Initial considerations and blend synthesis

a_g_ZIF-62 was selected as the MOF glass component because of the relatively large working range between *T*_g_ and the thermal decomposition temperature (*T*_d_), especially when compared to other coordination polymer glasses.^33,43^ For the inorganic glass component, an ultraphosphate glass was selected. Previously, chemical compatibility in a coordination polymer containing Zn^2+^, 1,2,4-triazole and orthophosphates has been demonstrated, and more recently composites between a_g_ZIF-62 and fluoroaluminophosphate glass were successfully synthesised. Here, the aim was to create a hybrid blend between a_g_ZIF-62 and 30Na_2_O–70P_2_O_5_, the latter was selected specifically for its low *T*_g_ (*T*_g_ = 181 °C).^45^

Given these considerations, a compositional series with varying atomic ratios of zinc and phosphorous was prepared: 1 : 1 a_g_ZIF-62: 30Na_2_O–70P_2_O_5_ (1 : 1 Zn : P), 1 : 3 Zn : P and 1 : 6 Zn : P, which corresponded to 72 : 28, 54 : 46, 30 : 70 wt% a_g_ZIF-62: inorganic glass, respectively. For clarity, the hybrid blends will be referred to as 1 : 1 Zn : P (1 : 1 blend), 1 : 3 Zn : P (1 : 3 blend) and 1 : 6 Zn : P (1 : 6 blend).

**Fig. 2:**
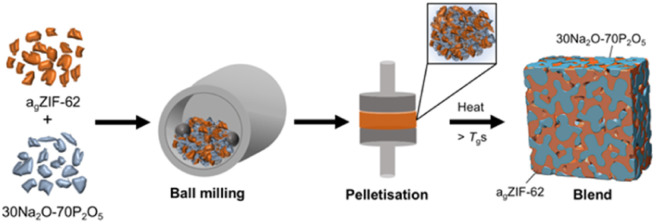
Schematic depiction of the synthetic procedure used to form the MOF glass–inorganic glass hybrid blends.

The structure and thermal response characteristics of the separate inorganic glass and a_g_ZIF-62 were confirmed by PXRD, FTIR, TGA and DSC (Fig. S1–S12†). The blends were synthesised according to the schematic in Fig. 2. Initially, powders of both glasses were ball-milled to produce a physical mixture before pelletisation and heating to 400 °C. This working temperature (*T*_w_) exceeded the *T*_g_s of the a_g_ZIF-62 (*T*_g_ = 334 °C) and the inorganic glass, enabling both glasses to enter a relatively low viscosity regime to facilitate liquid phase mixing before cooling to room temperature. The *T*_w_ needed to be sufficiently higher than the *T*_g_ of a_g_ZIF-62 to optimise mixing and the promotion of interfacial interactions, given the high viscosity of a_g_ZIF-62.

### X-ray diffraction, FTIR, Raman and ^1^H NMR spectroscopy

Powder X-ray diffraction (PXRD) patterns of the physical mixtures (*i.e.*, ball-milled powders of each glass prior to pelletisation and heating) display the expected amorphous behaviour of the starting materials (Fig. S13†). The weak, sharp peak at 2*θ* ∼27° in the inorganic glass is also evident in the 1 : 1 Zn : P physical mixture, in addition to two other peaks. However, these small peaks are minor and likely correspond to an unidentified crystalline impurity.

**Fig. 3:**
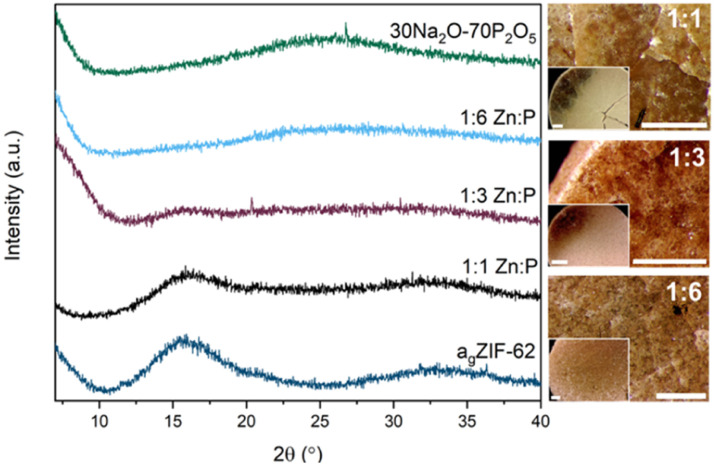
PXRD patterns of all three blends with the two parent glasses and optical microscopy images of the three blends. Scale bar for all images, including insets, is 1 mm.

PXRD of the blends confirm that the amorphous nature of the starting materials was retained successfully for all compositions post heating (Fig. 3). The absence of significant Bragg peaks indicates no recrystallisation of either glass or decomposition of the a_g_ZIF-62 to zinc oxide occurred. As expected, the patterns resemble the inorganic PXRD more closely with increasing proportion of inorganic glass, accompanied by a concomitant decrease in intensity of the diffuse scattering features associated with a_g_ZIF-62 (2*θ* ∼ 16° and ∼33°).

FTIR and ^1^H NMR spectroscopy were used to confirm the presence of the a_g_ZIF-62 phase in the blends. The ^1^H NMR spectra of the blends display all hydrogen environments of the benzimidazole and imidazole linkers, with a similar ratio of H_1_ to H_2_ (Fig. S14–S16†). A minor decrease in this ratio is observed in the 1 : 6 blend spectrum.

For the 1 : 1 sample, the FTIR spectrum of the blend closely matches the a_g_ZIF-62 spectrum (Fig. S17†). No differences in the FTIR spectrum are observed between the physical mixture and the blend (Fig. S18†). The same observations occur in the 1 : 3 sample. However, the 1 : 6 sample contains a_g_ZIF-62 peaks that have broadened on account of the increased proportion of the highly disordered inorganic glass. Again, little to no differences are observed post heat treatment (Fig. S18†).

The retention of the a_g_ZIF-62 phase is also evident in the Raman spectra of all three samples, which contain key a_g_ZIF-62 peaks, such as the C–N bond of the imidazolate ring at 1170 cm^−1^ observed in the literature (Fig. S19†).^46^ Raman spectroscopy was also used in an attempt to analyse the interface, which was done previously with similar materials.^45^ However, a high background signal, indicative of fluorescence in the spectra of all samples, led to no peaks being evident below 400 cm^−1^ (*i.e.*, the range in which changes in zinc bonding environment might be observed).

### Microscopy

Optical microscopy shows clear differences between the pellets pre (Fig. S13†) and post heat treatment. Optical images after heat treatment show flow between the two materials in some regions in the samples (Fig. 3).

**Fig. 4:**
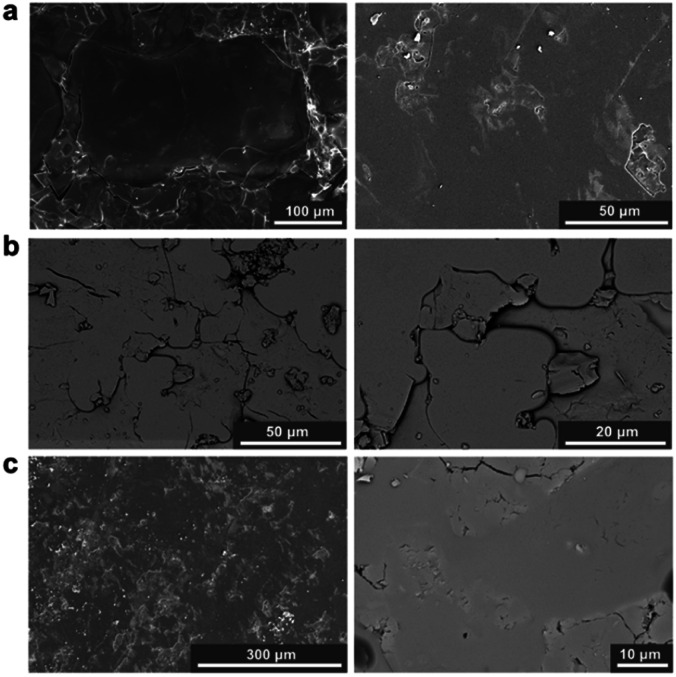
SEM images of the (a) 1 : 1 blend (b) 1 : 3 blend and (c) 1 : 6 blend. Secondary electron imaging mode was used for images (a and c) (left) and backscattered electron imaging mode was used for images (b and c) (right). Small artefacts are present in (a) (left) and (c) (left).

Furthermore, sample homogeneity was investigated by SEM analysis and EDS mapping. When compared to the 1 : 1 physical mixture (Fig. S20†), flow of the two glasses is evident in the 1 : 1 blend. SEM images of the blends show relatively smooth, homogenous surfaces, with several artefacts present on these surfaces (Fig. 4). EDS mapping identifies individual domains of the a_g_ZIF-62 and inorganic glass, shown by the zinc and phosphorous elemental maps (Fig. S21–S23†) throughout the samples.

**Fig. 5:**
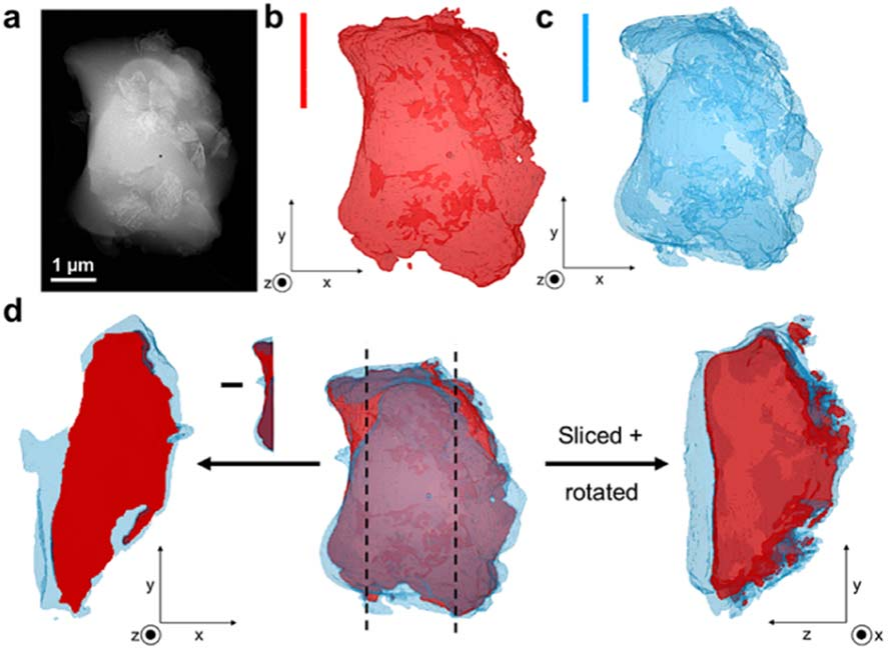
(a) Annular dark field STEM (ADF-STEM) image of the 1 : 3 blend, (b) ADF-STEM tomography of the inorganic phase of the grain, (c) ADF-STEM tomography of the a_g_ZIF-62 phase of the particle and (d) (centre) Combined structure of the studied particle showing close contact of the phases, with cross-sectional images on either side and vertical lines to indicate where the image was sliced. The left-hand image shows the exposed inorganic phase when the image is sliced on the left and confirms that the a_g_ZIF-62 is present above and below the inorganic phase. The right-hand image is the result of the particle being cut on the left and rotated.

To assess the mixing of the individual constituents further, chemically sensitive ADF-STEM tomography corroborated by two-dimensional STEM-EDS mapping was performed on the 1 : 1 and 1 : 3 blend samples (Fig. S24–S32†). ADF-STEM tomography probes atomic number density and enables intensity-based segmentation of domains with different density. Guided by intensity distribution analysis, the inorganic and MOF glass components were separated by intensity thresholding using edge spread function (ESF) curves in a non-standardised approach. The resulting images indicate the presence of the a_g_ZIF-62 around the denser, inorganic glass for both the 1 : 1 blend (Fig. S31†) and 1 : 3 blend (Fig. 5), confirmed by EDS elemental mapping (Fig. S26, S27, S29 and S30†).

Importantly, cross-sectional images of the sample indicate an exposed inorganic glass phase when the image is sliced, with the a_g_ZIF-62 above and below (Fig. 5d). This suggests the presence of a particle with both phases in close contact, instead of separate inorganic and MOF glass particles lying on top of each other. Overall, close mixing of the two components is evident.

### Thermal analysis

All three blends, physical mixtures and parent glasses were thermally characterised by TGA and DSC measurements (Fig. S33–S46†). TGA shows an earlier mass loss of the blends *versus* the parent glasses, indicating decreased thermal stability of the blends. Initial mass loss corresponding to surface water loss of 2.3% and 2.4%, and *T*_d_s of 236.8 °C and 249.7 °C are observed for the 1 : 1 and 1 : 3 blends, respectively (Fig. S36a, S37 and S38†). The TGA trace for the 1 : 6 blend displays an initial mass loss of 1.9% until 245 °C, corresponding to surface water loss (Fig. S39†). Above 245 °C there is minor mass loss of 4.3% until the main onset of decomposition at 388.6 °C. To understand this decreased thermal stability, TGA was performed on the pristine starting materials heat treated under the same experimental conditions used for the blends. A small decrease in thermal stability is observed (Fig. S4a and S9b†), but this is much smaller than the reduced thermal stability of the blends; the latter could be indicative of an interaction between the two components. This effect has been previously observed in polymer blends in which the resulting blend showed decreased thermal stability to the parent polymers because of interactions between them, which can be compositionally dependent.^47,48^ It has also been observed in polymer-carbon composites.^49^

To investigate the lower *T*_d_s of the blends relative to the parent materials further, PXRD analysis was done on all blend samples after heating them to 800 °C (Fig. S40†). Additionally, TGA experiments heating the samples to 500 °C were performed on the 1 : 1 and 1 : 3 blends followed by PXRD analysis to assess initial decomposition products. After 500 °C, diffuse scattering features associated with a_g_ZIF-62 (2*θ* ∼16° and ∼33°) are reduced, with weak Bragg peaks observed for the 1 : 3 blend. Upon heating to 800 °C, these diffuse scattering features are reduced further. Moreover, the ^1^H NMR spectrum of the 1 : 3 blend post heating to 500 °C (Fig. S41†) shows decomposition of the linkers, where the integrals of the benzimidazole and imidazole are not the expected ratio. The 1 : 6 blend shows multiple peaks in its PXRD pattern taken after TGA experiments at 800 °C. Most of these features are present in the ball-milled, pelletised and heat-treated inorganic glass control (Fig. S9c†). This indicates recrystallisation of the inorganic phase of the blend, where these peaks become visible as the inorganic content of the blends increases.

Given its higher onset of decomposition compared to the other samples, thermomechanical analysis (TMA) was used to further analyse the thermal behaviour of the 1 : 6 blend (Fig. S36b†). An inflection in the TMA curve occurs at 210.9 °C. TMA measurements of the pristine starting materials suggest that this inflection in the 1 : 6 blend probably corresponds to a softening of the inorganic component. A second inflection in the TMA curve was observed at 287.6 °C, which corresponds to the minor mass loss in the TGA trace observed before the main onset of decomposition at 388.6 °C.

Air stability tests were also performed on the pristine inorganic glass and the intermediate 1 : 3 blend (Fig. S36c and d†), where both samples retained their amorphous nature, shown by PXRD, after 10 days air exposure. However, the pristine glass formed a gel-like substance resulting from copious water uptake from the air, whereas the 1 : 3 blend retained its original shape, with several water droplets visible on its surface.

Prior to DSC measurement of the blends, DSC measurements were performed on the physical mixtures (Fig. S42–S44†). All three mixtures display an initial endothermic peak in the first upscan, which most likely corresponds to surface water loss as phosphate glasses _*x*_Na_2_O-(1 − *x*)P_2_O_5_ with *x* < 0.5 are hygroscopic.^25^ For the 1 : 1 physical mixture, a weak glass transition feature at 212 °C and 213 °C is present in the second and third DSC upscans respectively, before onset of decomposition. The 1 : 3 physical mixture displays a similar result, in which a single *T*_g_ at 211 °C and 213 °C is obtained from the second and third DSC upscans respectively before *T*_d_. After the initial endothermic feature in the 1 : 6 physical mixture in the first upscan, the inorganic *T*_g_ is visible at 193 °C before a second *T*_g_ at 241 °C. Again, a single *T*_g_ at 208 °C is evident in both the second and third DSC upscans, with *T*_g_s also visible on cooling.

*T*_g_ values of 211 °C and 215 °C are present in the first DSC upscans of the corresponding blends for the 1 : 1 and 1 : 3 samples respectively (Fig. S45–S47†). A change in the DSC signal of the 1 : 6 blend near the *T*_g_ values of the other blends can be observed in its first upscan, however it is difficult to accurately determine the *T*_g_ in this upscan as it does not resemble the expected shape of a *T*_g_ (Fig. S48†). Nonetheless, the *T*_g_ value of the other two blends is also evident in the 1 : 6 blend in the second and third upscans, as well as upon cooling and is at the temperature where the inflection in the TMA curve occurs.

**Fig. 6:**
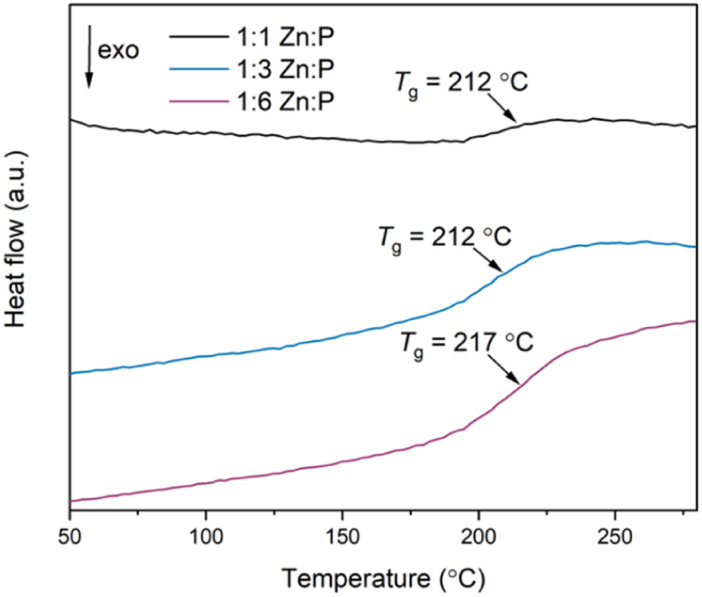
Second DSC upscans of all three blends using a heating and cooling rate of 10 °C min^−1^.

The second DSC upscans display a single *T*_g_ at 212 °C, 212 °C and 217 °C for the 1 : 1, 1 : 3 and 1 : 6 blends respectively, as observed in the DSC traces of the physical mixtures (Fig. 6). These are also reproduced in the third upscans. Interestingly, the *T*_g_s of the physical mixtures and corresponding blends are shifted higher than the inorganic glass *T*_g_ by more than 25 °C (Table 1). Overall, the strength of the DSC signal at *T*_g_ varies according to the mass of the phosphate glass phase.

**Table 1:** *T*_g_ and *T*_d_ values of the blends and starting materials

Material	*T*_g_ (°C)	*T*_d_ (°C)
agZIF-62	334	602
1 : 1 Zn : P blend	212	237
1 : 3 Zn : P blend	212	250
1 : 6 Zn : P blend	217	389
30Na_2_O–70P_2_O_5_	181	630

Determining the origin of this *T*_g_ value is not clear-cut. Two distinct *T*_g_s from the starting materials would be expected in a heterogenous composite with interlocked, separate domains of the individual constituents,^45^ whereas a single, new *T*_g_ value would be obtained if a homogenous blend has formed. The latter effect was observed after liquid phase mixing of ZIF-62 and ZIF-4-Co, [Co(Im)_2_], in which a melt-quenched glass with a single *T*_g_ distinct from either parent material was obtained, indicating the formation of a miscible blend.^35^ Previous studies on blending phosphate glasses indicate that blends consisting of different glasses produced two distinct *T*_g_s, but these were shifted relative to the pristine glasses because of the close mixing of the two phases.^50^ Here, we suggest that the *T*_g_s of the blends are related to the inorganic phase itself because the variable strength of the DSC signal at *T*_g_ is related to the mass fraction of this phase for constant sample mass used in the DSC studies. The a_g_ZIF-62 *T*_g_ is not present in the scans because the heating range used is lower than the *T*_d_ of the 1 : 1 and 1 : 3 blends (a_g_ZIF-62 *T*_g_ > blends' *T*_d_).

As such, we describe the products as immiscible blends, where miscibility can be identified by a new, single *T*_g_ in a mixture able to form a single phase.^50^

### Pair distribution function

Pair distribution function (PDF) analysis is an emerging technique for probing the interatomic distances in various materials.^41,51,52^ The PDF gives atomic distance information from atom–atom correlation histograms, providing insights into the local structure of materials.

The total scattering data obtained from X-ray synchrotron diffraction measurements were normalised to produce the structure factors, *S*(*Q*), of the parent glasses (Fig. S49†) and the blends (Fig. S50†). The associated PDFs were obtained by Fourier transforming these structure factors. The obtained PDF for a_g_ZIF-62 is consistent with literature PDFs of a_g_ZIF-62 and contains short-range (<6 Å) peaks at interatomic distances corresponding to those within the imidazolate ring of the linkers and the local Zn environment (A–E) present in the crystal structure of ZIF-62 (Fig. S51a†).^11,45^

Assigning peaks to the inorganic glass (IG) is more challenging given the lack of a crystalline analogue to which bond lengths could be compared. Nonetheless, the main correlations P–O, P–Na and Na–O were identified by comparing the PDF peaks to bond distances in the Na_3_P_3_O_9_ phase that sodium ultraphosphate glasses can recrystallise to, in addition to those obtained from other scattering experiments (Fig. S51b†).^25,53–55^ Importantly, comparing PDFs and *S*(*Q*)s of the starting materials before and after heating indicate no structural changes of either component glass resulting from the thermal treatment occurred (Fig. S52†).

**Fig. 7:**
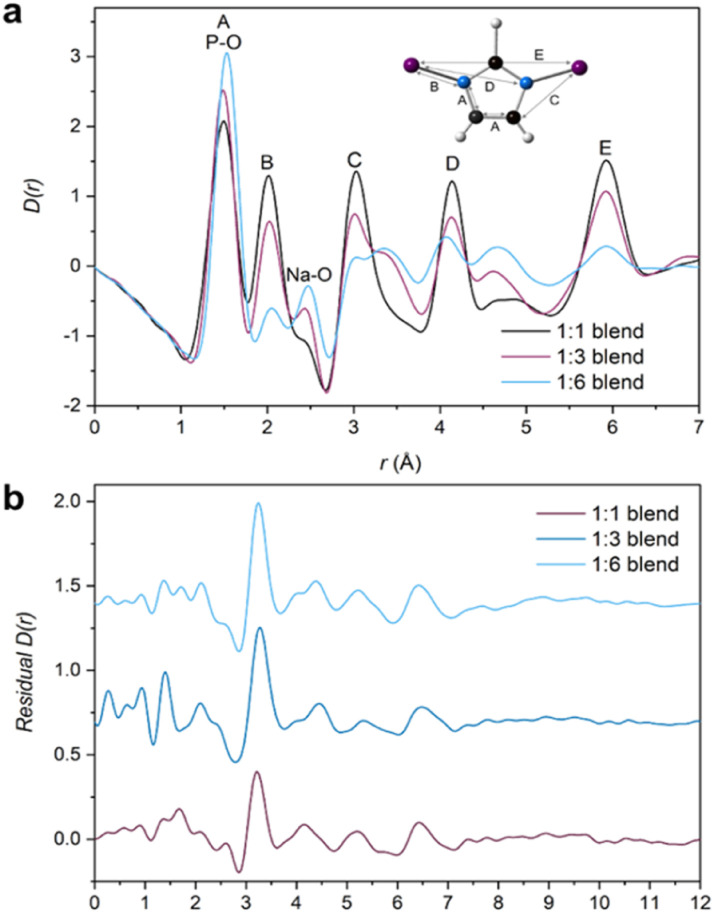
(a) X-ray pair distribution functions (PDF) of all three blends (1 : 3 unadjusted—see main text for details) with the assigned correlations from the parent glasses, namely A–E from the imidazolate ring and IG peak assignments at 1.5 Å and 2.48 Å for P–O and Na–O respectively and (b) Residuals obtained from the MLR fitting of the 1 : 1, adjusted 1 : 3 and 1 : 6 blend samples.

PDFs of the compositional blend series contain correlations from both a_g_ZIF-62 and the inorganic glass (Fig. 7a). Small differences in the position of the lowest-*r* correlation are observed in the three blend samples. This is likely the result of a change in the intensity ratio of two overlapping peaks in this region, correlation A from the a_g_ZIF-62 and the P–O bond from the IG. As expected, a_g_ZIF-62 peaks (A–E) increase with increasing a_g_ZIF-62 content in the blends, while the IG peak intensity increases with increasing inorganic glass content.

### Deciphering the interface

Despite the insights this initial PDF analysis provides, it does not yield information on interfacial interactions; these can potentially be deduced through the use of principal component analysis (PCA),^56^ Non-Negative Matrix Factorisation (NMF)^57^ or differential PDF.^58^ However, challenges arise with these approaches because of the small interaction volume between the two components relative to the bulk of the material and care must be taken during data interpretation.^45,57^ Here, multiple linear regression (MLR) analysis was performed on the total scattering data, which has previously been applied to X-ray diffraction patterns to assess the surface area of mesoporous materials^59^ and on X-ray fluorescence spectrometry data.^60^

The blend samples were fitted using the two PDFs of the starting materials. Intuitively, the blend PDFs can be thought of as a combination of the starting materials' PDFs in varying proportions, with a potential interface between them. However, careful data interpretation is required as size of interface contributions are at the limit of accuracy of the technique. Moreover, only two PDFs are used for the fitting and some of the interface's contribution to the blends' PDF could be inadvertently fitted.

By using a linear combination of the starting materials' PDFs to fit the blend PDF, the residual could yield features not described by the two end members, which could be ascribed to the interface. Each sample PDF was fitted according to eqn (1), where *C*_1_ and *C*_2_ are related to the proportion of a_g_ZIF-62 and IG respectively. The effect of pelletisation, ball, milling and heat treatment on the starting materials has been accounted for in the fact that there is minimal deviation between the starting material *D*(*r*)s (Fig. S52†).

1 Blend *D*(*r*) = *C*_1_(a_g_ZIF-62 *D*(*r*)) + *C*_2_(IG *D*(*r*))

Initially, fitting of the blend samples yielded reasonable *R*^2^ values of 0.985 and 0.963 (*i.e.*, close to 1) for the 1 : 1 and 1 : 6 blends respectively (Table S2†). The residuals from these fits are mostly flat except for several unassigned features and a key feature at 3.22 and 3.25 Å for the 1 : 1 and 1 : 6 samples, respectively (Fig. S54†). This peak is not present in either starting material or the empty capillary used for data correction and could represent an atom–atom correlation at the interface between the inorganic and MOF glasses.

However, the form of the residual for the 1 : 3 blend deviates from the other two samples, with a *R*^2^ value of 0.932 and a negative peak at *r* = 1.6 Å, which corresponds to the Si–O bond length in the borosilicate glass which was used as the sample container. The presence of a positive or negative characteristic PDF peak from the capillary is routinely used to confirm the efficacy of the capillary subtraction. Here, strong peaks from the samples partially overlap the Si–O peak from the capillary making this diagnostic difficult, but it was detected in the linear regression differential. This suggests that the sample container had been over-subtracted during data correction and normalisation. Further support for this is seen in the position of the proposed interface peak which occurs in the residual at 3.33 Å (*i.e.*, slightly shifted from the position of this peak from the other samples). This shift could be caused by an additional negative peak at 3.1 Å corresponding to the Si–Si distance in borosilicate glass removing intensity from the low-*r* side of the positive peak (Fig. S56†). As such, the total scattering data from this sample were recorrected using the empty capillary (container) scattering reduced by 7.5% to account for this issue. This new adjusted PDF shows minor deviation from the unaltered/unadjusted 1 : 3 blend, with negligible differences between the *D*(*r*) and *S*(*Q*)s (Fig. S55†). By subsequently performing MLR on this adjusted sample, the features in the residual associated with mis-subtraction of the capillary scattering were suppressed (Fig. 7b). No evidence of mis-subtraction was observed in the other two blend samples and so no adjustments were made.

Multiple linear regression analysis was also performed on the physical mixtures (Fig. S57†) to highlight differences post heat treatment. Adequate fitting with reasonable *R*^2^ values (Table S2†) were obtained and a peak at *r* ∼ 3.25 Å is evident in all three residuals. A negative peak at *r* = 1.6 Å was not observed and so no further adjustments were made.

**Fig. 8:**
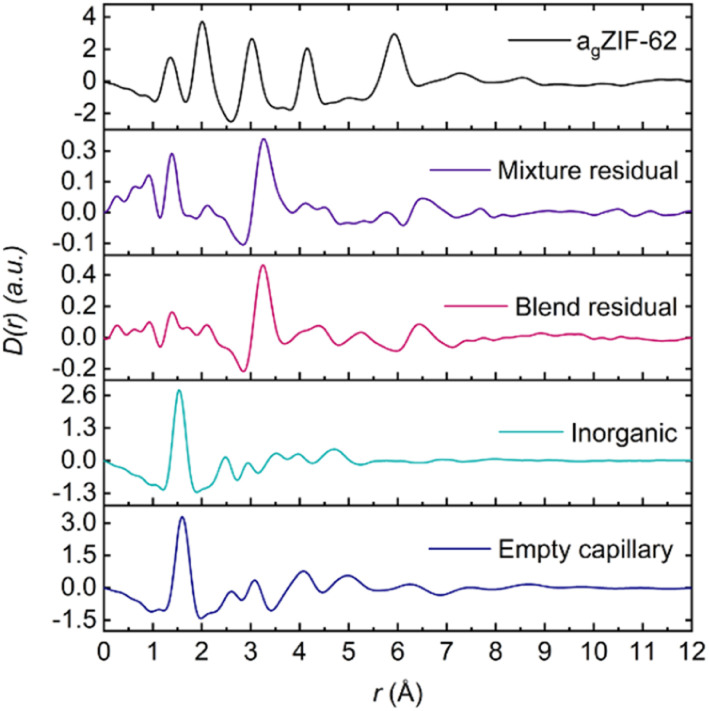
Average residual of the physical mixtures and blends plotted against the starting materials' *D*(*r*).

The observed peaks are weaker than those in the blend residuals (Fig. 8), qualitatively suggesting more interfacial interactions post heating. This difference is clearer when comparing the average blend residual (potential interface peak: *r* = 3.24 Å) with the average physical mixture residual (potential interface peak: *r* = 3.26 Å) and in the fact that the linear combination of the starting materials' PDFs fit the PDFs from the mixtures better (Fig. S58†). This is evident in a plot of *R*^2^ values against composition, in which *R*^2^ values of the blends deviate more from an ideal fit (*R*^2^ = 1) than those of the physical mixtures. Overall, the residuals obtained for the physical mixtures show negligible differences between the corresponding blends for the 1 : 1 and 1 : 3 compositions (Fig. S60†). There is deviation for the 1 : 6 compositions at higher *r* values, but the origin of these features is unclear.

Assigning this potential interface peak to a specific correlation is not trivial. However, the structures of crystalline zinc phosphate phases (Fig. S61†) have been used to obtain sensible bond lengths involving O, P and Zn atoms. These suggest that the interface peak arises from a Zn⋯P distance in which the Zn and P atoms are bridged *via* an oxygen atom. Furthermore, the difference in peak amplitudes in the mixtures and blends can be rationalised when considering the plethora of reactions that can be initiated *via* ball-milling.^61,62^ More specifically, the Zn–O–P interaction has been mechanochemically induced in the literature in which a zinc phosphate coordination polymer was synthesised by hand grinding alone.^44^ Here, we tentatively suggest that ball-milling initiates the interfacial interactions and subsequent heat treatment strengthens these interactions even further, culminating in a larger interface peak.

These results could be a possible explanation for the difference in thermal stability between the blends and the parent materials under the same experimental conditions (Fig. S36a†). A change in thermal properties has been demonstrated in ternary phosphate glasses where ZnO (essentially a source of Zn^2+^ ions) has been added as a modifier. The ZnO culminates in weaker P–O–Zn bonds throughout the glass network, replacing the stronger P–O–P bonds, giving a less compact glass network with decreased crosslink density and more ionic character.^63,64^

### ^31^P NMR

**Fig. 9:**
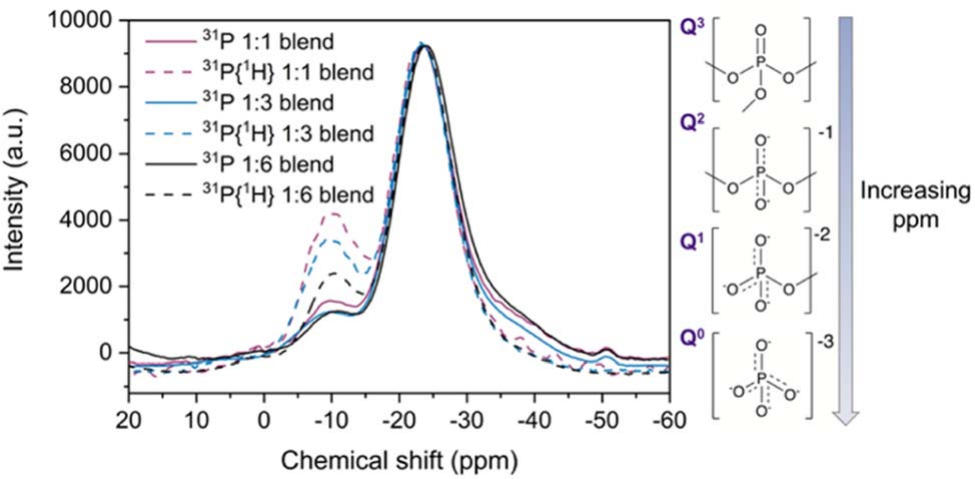
^31^P solid state NMR and ^31^P{^1^H} CP NMR (dotted lines) of the three blends, with insets showing the phosphate environments present in the pristine inorganic glass.

Solid state magic angle spinning ^31^P nuclear magnetic resonance (MAS NMR) spectroscopy was performed to investigate changes in the phosphate tetrahedra in the new blend materials, given the relatively high sensitivity of the spin ½^31^P nuclei. Direct NMR and ^31^P{^1^H} cross polarisation (CP) NMR measurements (Fig. 9) were carried out to analyse the effect of adding a_g_ZIF-62 to the phosphate glass.

Fitting the ^31^P NMR spectra of the pristine glass yielded (Fig. S62d†) peaks at −26.4 ppm and (seen as a shoulder in the spectra) at −38.5 ppm (Table S4†). The main peak of the pristine glass at −26.4 ppm corresponds to phosphorus in a Q^2^ unit, which contains two bridging oxygen atoms (Fig. 9 inset) and is a plausible site for coordination to metal ions such as zinc.^65–67^ This peak displays a shift towards less negative ppm values when a_g_ZIF-62 is added to the blends (Fig. S63 and Table S4†). Previous reports on Na_2_O–ZnO–P_2_O_5_ glasses indicate that the presence of Zn centres can induce a shift in the Q^2^ signal of the ^31^P NMR spectra^65,68^ and more generally, the chemical shift of ^31^P nuclei is sensitive to the modifier cations bonded to the non-bridging oxygen atoms.^67^ The peak shift in the blend samples could be indicative of a potential P–O⋯Zn interaction.

The peak at −38.5 ppm in the pure inorganic glass spectrum can be assigned to Q^3^ tetrahedron containing three bridging oxygens and one terminal P=O.^66^ This peak decreases in intensity in the blend samples, which could suggest a strong polarisation of the P=O bonds to the Zn centres in the blends, again resulting in a shift of their ^31^P NMR signal. The pure glass ^31^P spectrum also shows additional peaks between 0 and −11 ppm, which are in the region of Q^0^ (0 ppm) and Q^1^ tetrahedra (−11 ppm). The origin of these peaks likely correlates to adventitious hydrolysis of the glass, depolymerising the glass network over time. Only one of these peaks (Q^1^ at −10.7 ppm) is present in the blend spectra, with negligible shift. Several other peaks are present in the pure inorganic glass spectrum, with a possible explanation discussed in the ESI (Fig. S64 and S65†).

Complementary to direct NMR studies, cross polarisation (CP) NMR measurements were also performed. Comparison of direct and CP measurements reveals a decrease in the relative intensity of Q^3^ signal with respect to the Q^2^ major peak in the CP spectra. A systematic increase in the intensity of the −10.7 ppm signal in the CP spectra relative to direct NMR spectra was also observed for the blend samples (Table S5†). This can be attributed to the proximity to protons in the imidazolate ligands of the a_g_ZIF-62 inducing an increase of the intensity in the CP spectra by polarisation transfer between ^31^P nuclei in the phosphate glass and ^1^H nuclei in the imidazolate rings.

### Nanoindentation

Nanoindentation measurements were carried out to assess the mechanical properties of these new materials and to verify the extent of mixing between the phases. However, reliable data could only be obtained for the two blends with the lower inorganic glass content (1 : 1 and 1 : 3 blend) and for the starting materials. For the blend with the highest phosphate glass content, there was a high tendency of the material to stick to the tip post indentation, which made subsequent indentation tests meaningless as they were dominated by the changed contact surface.

**Fig. 10:**
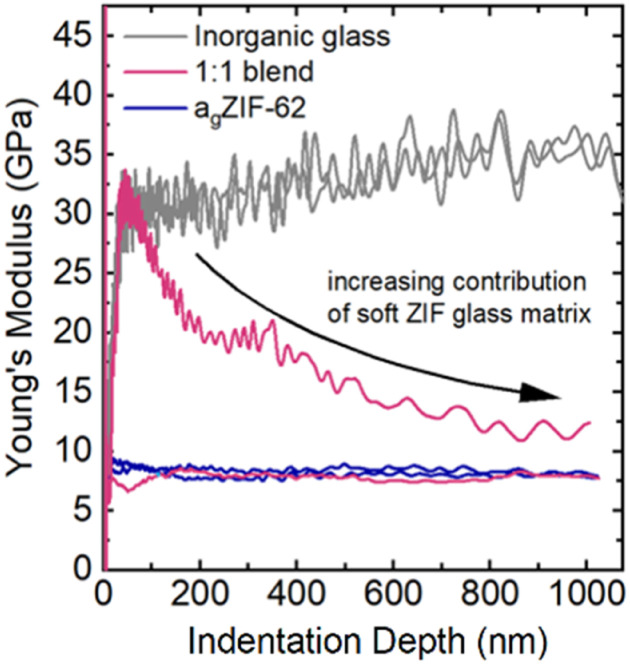
Exemplary depth-profiles of the Young's modulus of the 1 : 1 blend (pink curve) revealing either indentation behaviour of the a_g_ZIF-62 (*E* ∼ 7–8 GPa, blue line) or contribution of the stiffer phosphate glass (E ∼ 35 GPa, grey line) on the initial contact with rapid decrease towards higher depths when a larger volume is indented. Indentation tests were performed on different parts of the 1 : 1 blend where initially regions of inorganic glass and a_g_ZIF-62 are indented, with increasing contribution from a_g_ZIF-62 at larger indentation depths.

The indentation derived Young's modulus (*E*), an indicator of the stiffness of a material under tension or compression, was obtained for a_g_ZIF-62 (∼7–8 GPa), whilst the stiffer inorganic glass exhibited an *E* value of about 35 GPa (Fig. 10).

Previous nanoindentation measurements on a_g_ZIF-62 composites with a fluoroaluminophosphate glass revealed regions of low and high modulus (*E*), interpreted as a heterogenous interlocking of the a_g_ZIF-62 and inorganic glass domains.^45^ In the well-mixed blends prepared in this study, no distinct domains of hard and soft glass are detectable. Instead, their response was investigated by deriving a distribution of average *E* in between the starting materials' *E* values over hundreds of indentation tests. This contrast with the study on composites highlights how the selection of inorganic glass can influence the nature of the produced composite or blend, and consequently affect the resulting mechanical properties.

**Fig. 11:**
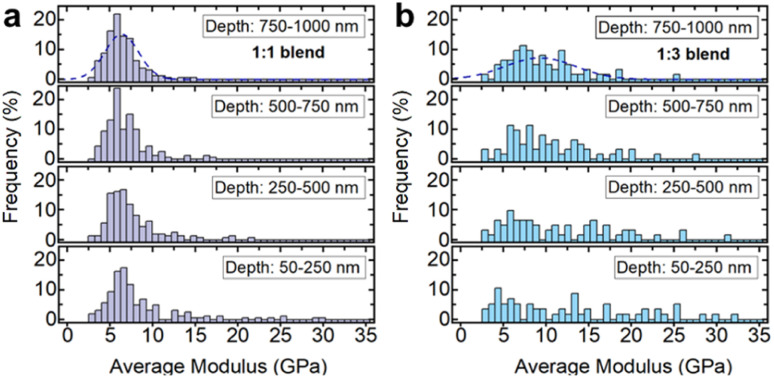
Nanoindentation results on 1 : 1 and 1 : 3 blends of inorganic glass (30Na_2_O–70P_2_O_5_) and a_g_-ZIF62, distributions of the indentation moduli depending on the depth-range used for averaging for the (a) 1 : 1 blend and (b) 1 : 3 blend.

Due to large differences in the elastic moduli of the two phases and the fine-grained powders used in the blend, the stiffer inorganic glass was only detectable if the indentation tip exclusively interacted with an inorganic glass particle (Fig. 10 and 11). If both phases were indented or the interacting volume was larger than the volume of the inorganic glass grain, the total indentation response was dominated by the softer a_g_ZIF-62 phase, which leads to a rapidly decreasing modulus at higher indentation depths.

Histograms of the average moduli of both samples (Fig. 11a and b) show that while at low depths higher moduli, *i.e.*, inorganic glass particles, are still measured, the large moduli tail largely mostly disappears for higher indentation depths. The 1 : 3 blend (Fig. 11b) contains a larger fraction of stiffer, inorganic phase, resulting in a higher probability of measuring the inorganic phase at low depths, or seeing its contributions at larger indentation depths.

The nanoindentation experiments confirm the creation of a mixed, granular immiscible blend with contributions from both phases, in agreement with the electron tomography and thermal analysis results.

## Conclusions

A compositional series of a_g_ZIF-62 and an ultraphosphate glass, 30Na_2_O–70P_2_O_5_, was synthesised to combine the mechanical and chemical properties of the inorganic glass with the chemical versatility offered by glassy MOFs.

Reasonable chemical compatibility was realised between the glasses, with no evidence of decomposition or recrystallisation from PXRD, FTIR, Raman or ^1^H NMR spectroscopy. The thermal behaviour of the materials was assessed, showing *T*_g_s shifted relative to that of the inorganic glass. Additionally, TGA revealed decreased thermal stability relative to the starting materials and their heat-treated controls, suggesting a destabilising effect of interfacial interaction. The *T*_g_s were not compositionally dependent, which is sometimes observed in polymer blends, but the *T*_d_s varied according to the amount of inorganic phase present.

The nature of the interfacial interaction was examined using multiple linear regression of total scattering data, from which a P–O⋯Zn interaction was suggested. This new, previously unreported method of analysing interfacial interactions could be extended to other composite and membrane systems, where important interactions at the interface remain challenging to understand. Additionally, the sensitivity of the regression analysis to correction of the total scattering data was highlighted, suggesting careful interpretation when analysing interfacial interactions.

The multiple linear regression results were consistent with ^31^P NMR data and chemically sensitive electron tomography results, the latter of which showed close mixing of the two constituent phases, in which mixed, and not completely separate domains, are present, consistent for both the 1 : 1 and 1 : 3 blends. The segmentation used to acquire these images is not a standard approach for analysing composites and blends and instead describes a new method of using edge spread function (ESF) curves. This visualisation can be extended to other systems in which structure and extent of mixing is linked to a material's properties.

Such in-depth characterisation techniques may be applied to a host of novel composites where the interface is critical to determining overall mechanical performance.

Furthermore, nanoindentation measurements on the 1 : 1 and 1 : 3 blends indicated a distribution of average moduli, indicating the presence of a well-mixed blend with fine microstructure in both samples. This was in contrast to previous nanoindentation results on a_g_ZIF-62-inorganic glass composites, showing that judicious selection of the inorganic glass directly affects the thermal and mechanical properties of the end product.

Overall, the successful fabrication of this compositional series highlights the benefits of combining existing materials, inorganic and hybrid, to create novel glasses with markedly different mechanical and thermal properties. By doing so, the respective advantages of structurally and chemically diverse glass types can be combined, which opens the door to a realm of potential new applications. Developments in understanding and creating porous MOF glasses^14^ can lead to functional materials, in which the issues with producing bulk MOF glasses could be offset by combining them with alternative materials. Furthermore, exploration of these materials also contributes to understanding the underlying interfacial interactions in a more general way for select applications, *e.g.*, in adherent laminate structures or for hybrid solder applications and proton conductivity.

## Data availability

The experimental data that support the findings of this study are provided in the manuscript, its ESI†, or are available at https://doi.org/10.17863/CAM.101850.

## Author contributions

AMC, DAK, TDB and LW designed the project. AMC wrote the manuscript with input from all authors. AMC synthesised a_g_ZIF-62 starting material and blend samples and BPR synthesised 30Na_2_O–70P_2_O_5_ glass. AMC and CCB analysed the inorganic glass. Thermal analysis, PXRD, FTIR, ^1^H NMR and SEM-EDS were performed and analysed by AMC. Annular dark field scanning transmission electron microscopy (ADF-STEM) tomography and X-ray energy dispersive spectroscopy (EDS) was performed and analysed by JES and SMC. ^31^P NMR data was collected and processed by AM and RMB. Total scattering data was collected by AMC, CCB, AFS, GPR, DJMI and DAK. PDF analysis was done by AMC with input from DAK and CCB; interfacial analysis on PDF data was done by AMC, CCB, AFS and DAK. Raman spectroscopy and nanoindentation was performed and analysed by RS and LW. DAK, TDB and LW supervised the project and precured the funding.

## Conflicts of interest

There are no conflicts to declare.

## Supplementary Material

SC-014-D3SC02305B-s001
