# Supplementary material for: Structural insights into hybrid immiscible blends of metal–organic framework and sodium ultraphosphate glasses
Source: Chem Sci. 2023 Sep 12;14(42):11737–48. doi: 10.1039/d3sc02305b (PMC10619634; doi:10.1039/d3sc02305b)
Supplement: SC-014-D3SC02305B-s001 [file SC-014-D3SC02305B-s001.pdf]

Electronic Supplementary Material (ESI) for Chemical Science.
This journal is © The Royal Society of Chemistry 2023

Structural insights into hybrid immiscible blends of metal-organic framework and sodium ultraphosphate glasses

Ashleigh M. Chester,[a] Celia Castillo-Blas,[a] Roman Sajzew,[b] Bruno P. Rodrigues,[b] Ruben Mas Balleste,[c,d] Alicia Moya,[c] Jessica E. Snelson,[e] Sean M. Collins,[e] Adam F. Sapnik,[a] Georgina P. Robertson,[a,f] Daniel J.M. Irving,[f] Lothar Wondraczek,[b] David A. Keen[g] and Thomas D. Bennett[a*]

[a]*Department of Materials Science and Metallurgy, University of Cambridge, Cambridge, CB3 0FS, UK*
E-mail: tdb35@cam.ac.uk

[b]*Otto Schott Institute Materials Research, University of Jena, Fraunhoferstrasse 6, 07743 Jena, Germany*

[c]*Department of Inorganic Chemistry, Universidad Autónoma de Madrid, 28049, Madrid, Spain*

[d]*Institute for Advanced Research in Chemical Sciences (IAdChem), Universidad Autónoma de Madrid, 28049, Madrid, Spain*

[e]*School of Chemical and Process Engineering, School of Chemistry, and Bragg Centre for Materials Research, University of Leeds, Woodhouse Lane, LS2 9JT, UK*

[f]*Diamond Light Source Ltd., Diamond House, Harwell Campus, Didcot, Oxfordshire OX11 0DE, UK*

[g]*ISIS Facility, Rutherford Appleton Laboratory, Harwell Campus, Didcot, Oxfordshire OX11 0QX, UK*

Table of contents

1. **ZIF-62 glass characterisation**
    1.1 Crystalline ZIF-62 characterisation
    1.2 Chemical analysis: CHN/^{1}H NMR, density
    1.3 Controls: PXRD, TGA
    1.4 Thermal analysis: DSC, TMA
    1.5 Microscopy
2. **Inorganic glass characterisation**
    2.1 Compositional analysis and density
    2.2 Controls: PXRD, TGA
    2.3 Thermal analysis: DSC, TMA
    2.4 Microscopy
3. **Blend characterisation**
    3.1 PXRD analysis
    3.2 ^{1}H NMR spectroscopy
    3.3 FTIR spectroscopy
    3.4 Raman spectroscopy
    3.5 SEM-EDS
    3.6 Scanning transmission electron microscopy (STEM) and STEM-EDS
    3.7 Thermal analysis: TGA, DSC, TMA
    3.8 PDF analysis
    3.9 ^{31}P NMR spectroscopy

1. ZIF-62 starting material characterisation

1.1 Crystalline ZIF-62 characterisation

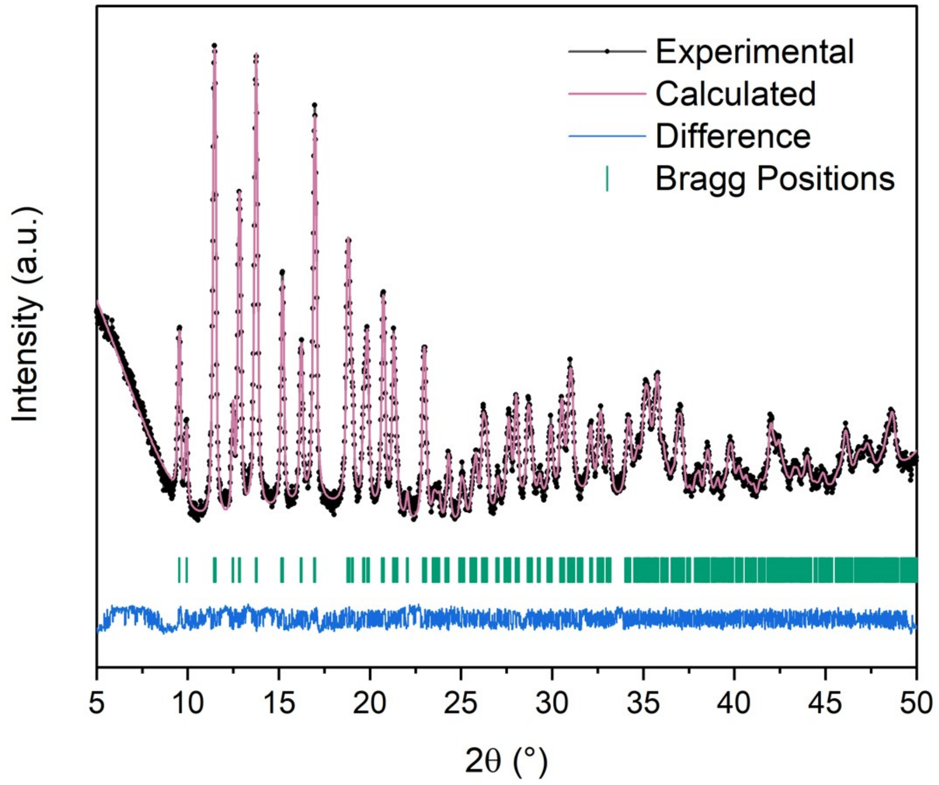

Figure S1. Pawley refinement of the crystalline batch of ZIF-62 used for the a_gZIF-62 synthesis, with experimental (grey dots), calculated (pink line), difference plot [$(I_{obs}-I_{calc})$] (blue line) and Bragg positions (green ticks). Obtained unit cell: R_{wp} = 5.40%, R_p = 3.90%, *a* = 15.469(4), *b* = 15.573(5), *c* = 17.988(5) Å, space group *Pbca*. Zero error 0.230(4).

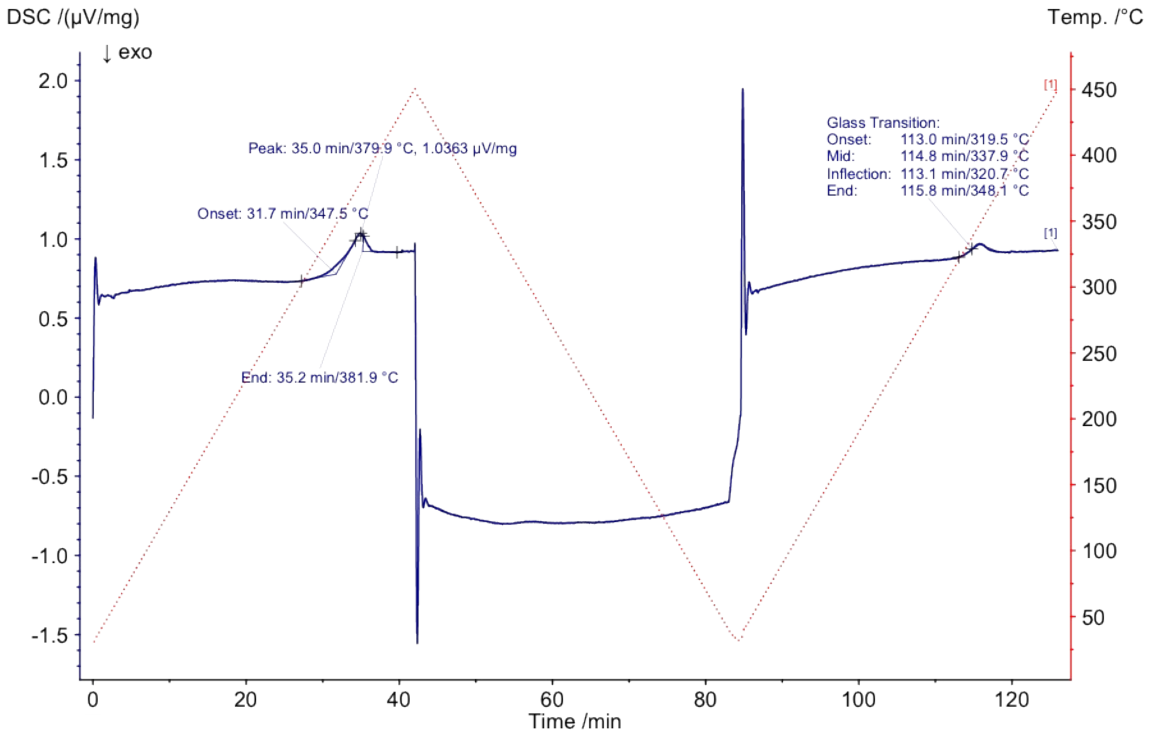

Figure S2. Full DSC up and down scans of the crystalline batch of ZIF-62 used for the glass synthesis, using a heating and cooling rate of 10 °C min^{-1}. A melting endotherm at 380 °C was observed in the first upscan, with a T_g at 338 °C observed in the second upscan. Dotted red lines denote temperature during the scan.

1.2 Chemical analysis of a_gZIF-62

CHN microanalysis indicated a composition of 39.55 C%, 3.10 H% and 25.83 N%, with little deviation from the CHN composition reported for mechanochemically synthesised a_gZIF-62 in the literature[1] , and acid-digested ^{1}H NMR of a_gZIF-62 confirmed the linker ratio of bIm: Im. The density found was 1.59 ± 0.004 g cm^{-3}, in line with reported density values for a_gZIF-62 of similar bIm: Im ratio by He pycnometry.[2] However,

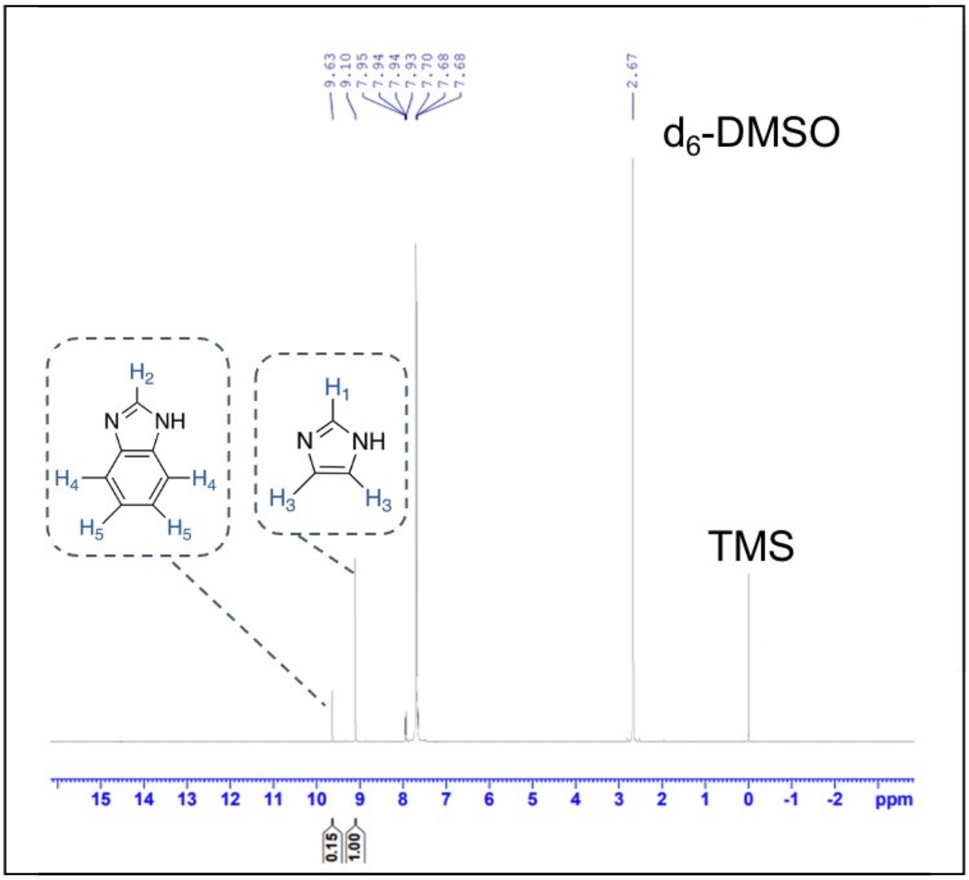

Figure S3. ^{1}H NMR spectrum of a_gZIF-62. δ_H (500 MHz, DCl (35%)/D_2O: DMSO-d_6 (1:5), TMS) 9.63 (1H, s, H_2), 9.10 (1H, s, H_1), 7.94 (2H, q, H_4), 7.70 (1H, s, H_3), 7.68 (2H, d, H_5), 2.67(DMSO), 0.00 (TMS).

1.3 Controls of a_gZIF-62

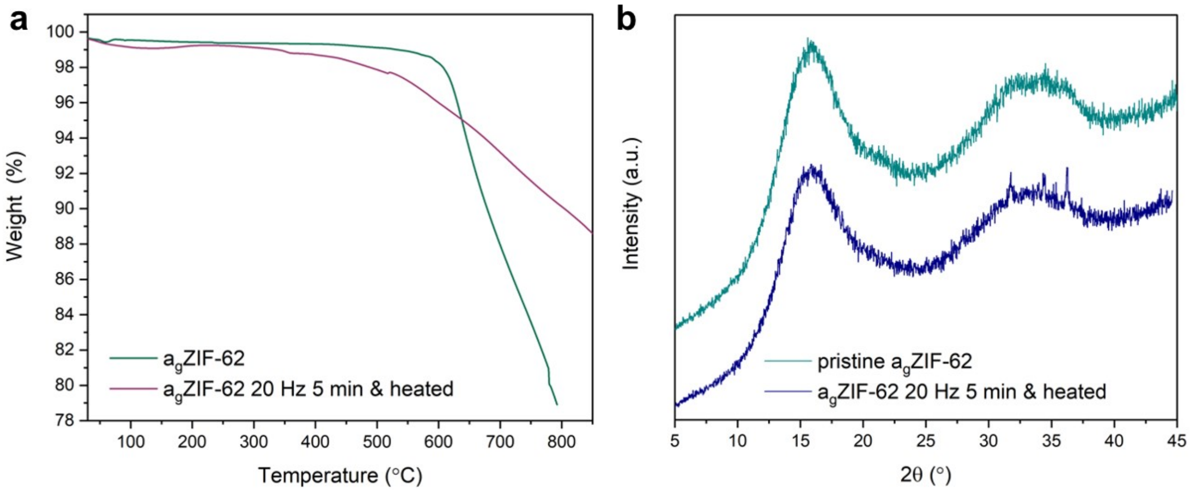

Figure S4. a. TGA profiles and **b.** PXRD patterns of the pristine a_gZIF-62 starting material and ball-milled and heat treated a_gZIF-62 control. Small Bragg peaks corresponding to ZnO appear in the heat treated control.

1.4 Thermal analysis of a_gZIF-62

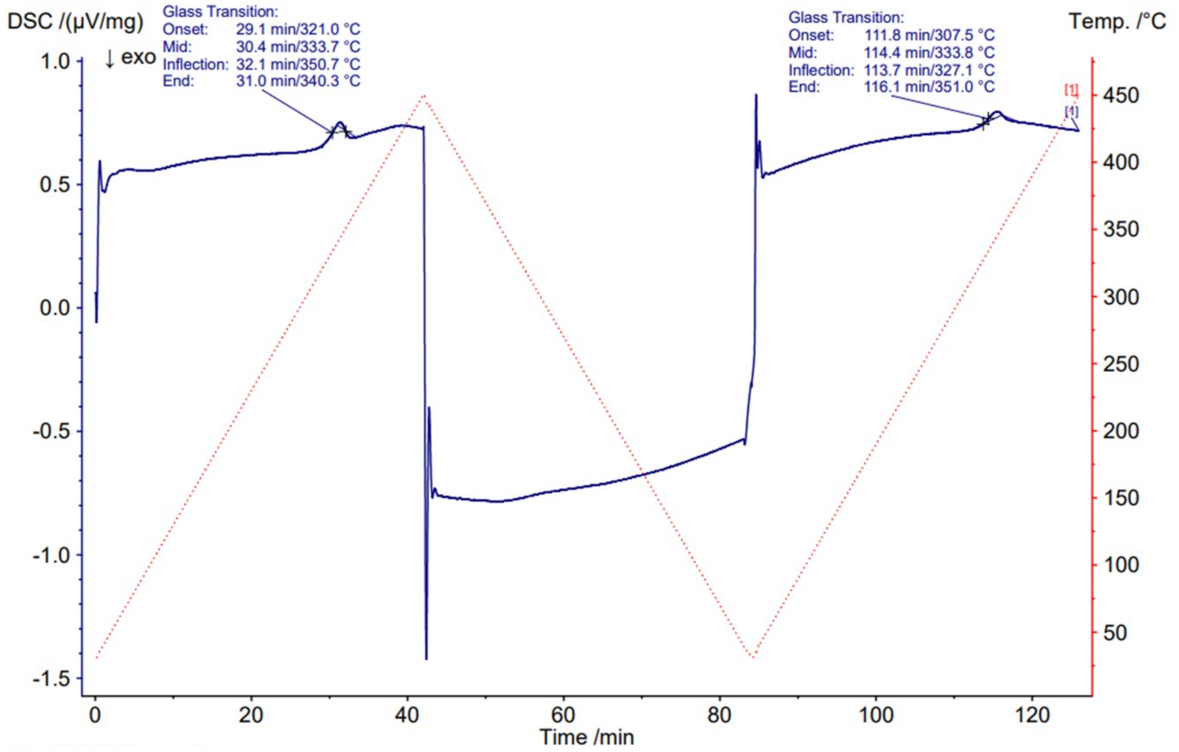

Figure S5. Full DSC up and down scans of a_gZIF-62 starting material using a heating and cooling rate of 10 °C min^{-1}.

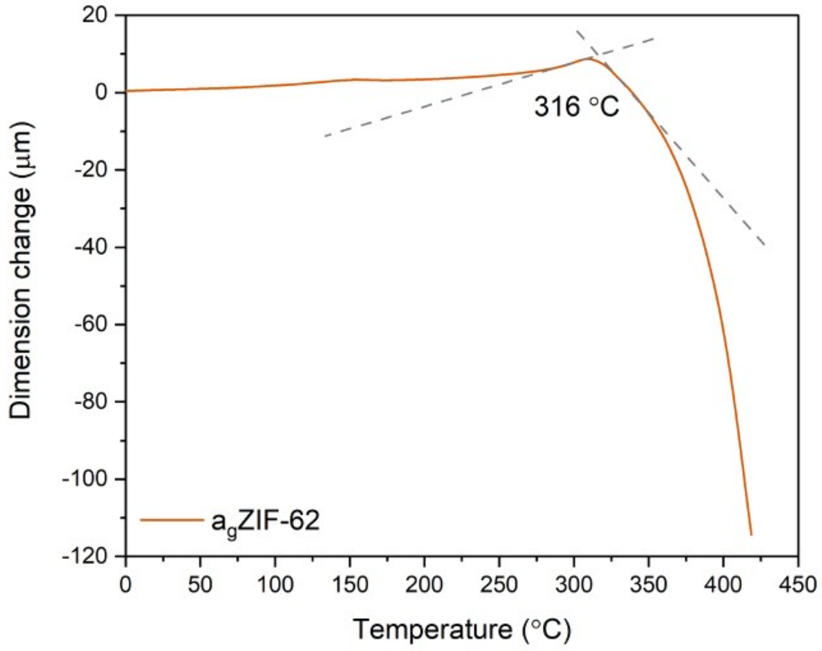

Figure S6. Thermomechanical analysis of a_gZIF-62 starting material, showing an inflection at 316 °C. A heating range of 30-450 °C was used.

1.5 Microscopy

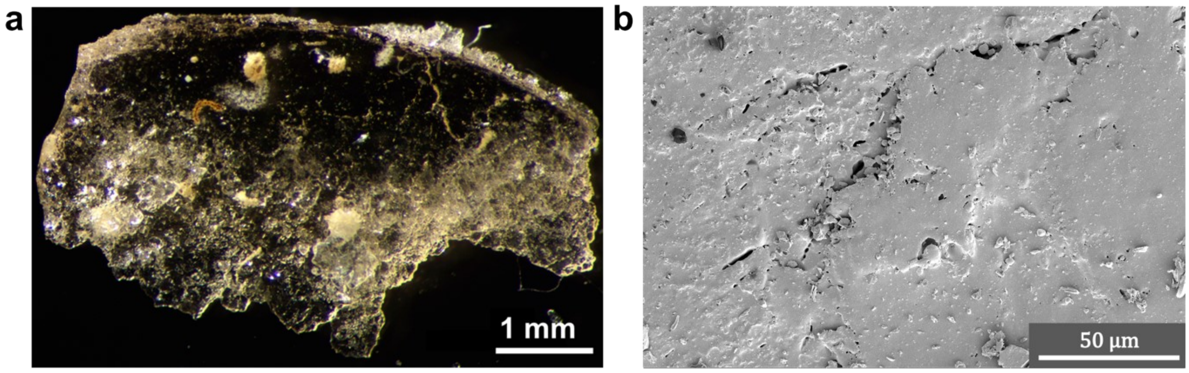

Figure S7 a. Optical microscopy and **b.** SEM images of a_gZIF-62 starting material using secondary electron imaging mode.

2. Inorganic glass characterisation

2.1 Chemical analysis and density measurements

The density of the $30Na_2O$-$70P_2O_5$ glass was 2.38 ± 0.0002 g cm^{-3}, in close agreement to literature values for glasses of similar composition.[3]

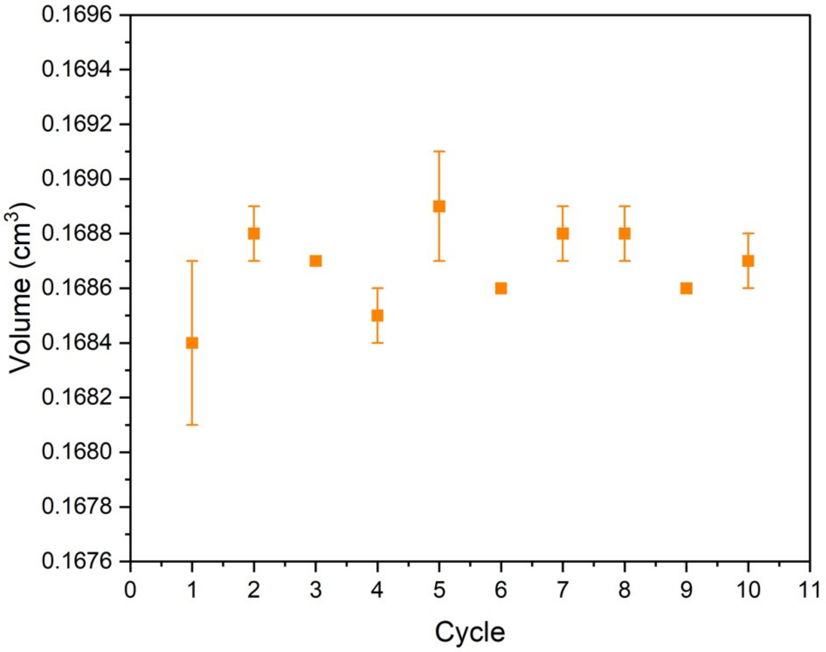

Figure S8. Volume of $30Na_2O$-$70P_2O_5$ measured over 10 cycles using He pycnometry, where the sample mass was 0.4015 g.

WDX spectroscopy indicated minor deviation of the measured results from the nominal composition.

Table S1. WDX spectroscopy on pristine $30Na_2O$-$70P_2O_5$.

**Nominal $30Na_2O$-$70P_2O_5$ Measured $29Na_2O$-$71P_2O_5$**			
	Na atom (%)	P atom (%)	O atom (%)
Nominal	10.34	24.14	65.52
Measured	9.97 ± 0.19	23.10 ± 0.22	66.92 ± 0.24

2.2 Controls of $30Na_2O\text{-}70P_2O_5$

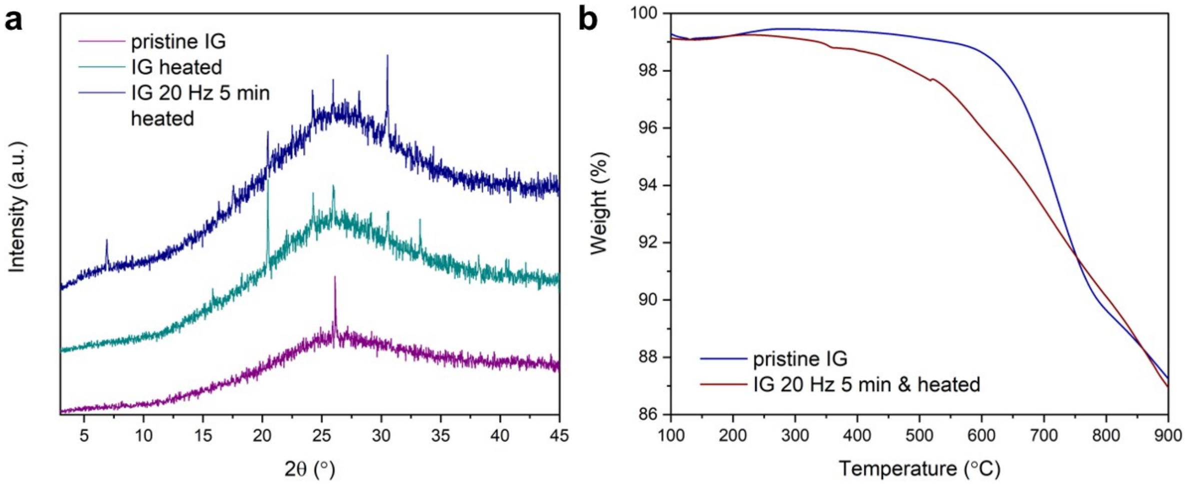

Figure S9. a. PXRD patterns and **b.** TGA profiles of the pristine $30Na_2O\text{-}70P_2O_5$ glass and ball-milled and heat treated $30Na_2O\text{-}70P_2O_5$ control. The peaks present in the IG heated (cyan curve) and ball milled, pelletised and heated control (dark blue curve) likely correspond to recrystallisation to a sodium phosphate or sodium hydrogen phosphate phase which could not be identified from the small number of weak peaks observed. These peaks are absent in the hybrid blends, which suggests a_gZIF-62, and the potential interactions between the glasses in the physical mixtures, stabilises the inorganic glass from recrystallisation.

2.3 Thermal analysis

The T_gs were assigned using the DSC second upscans instead of the first because after heating and cooling, surface water has been driven off and the thermal history of the glasses has been reset.

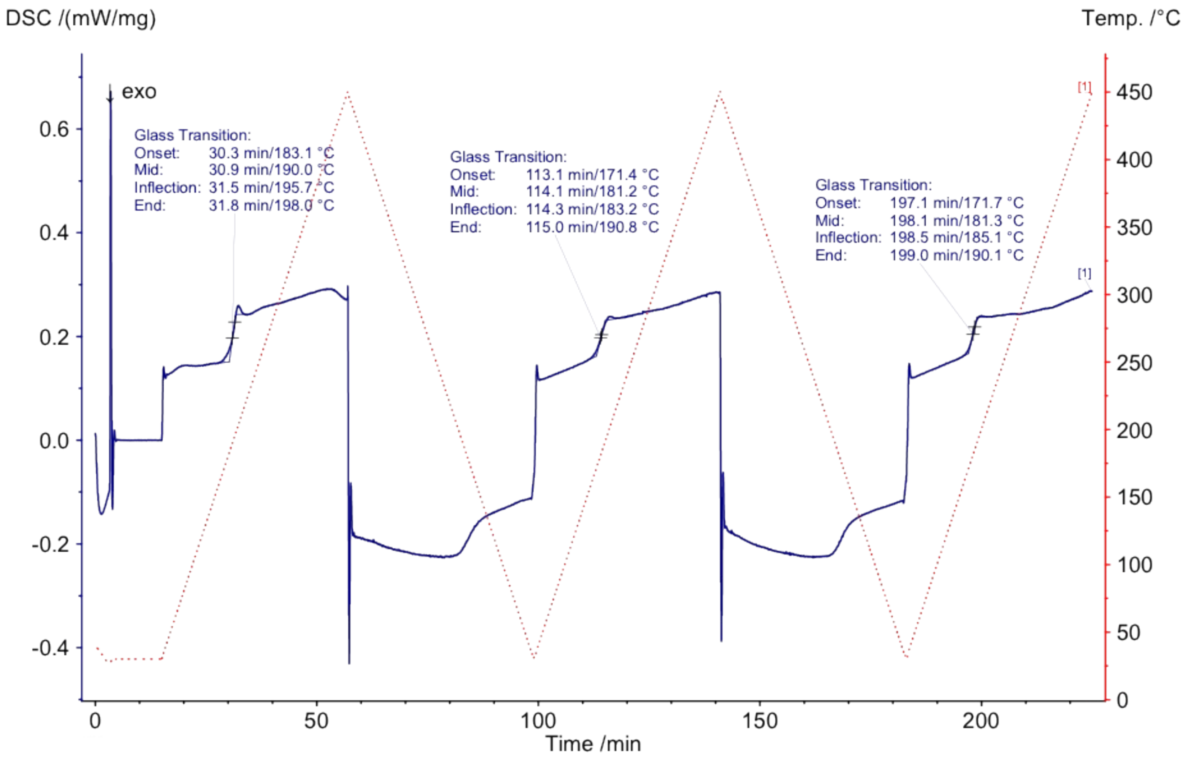

Figure S10. Full DSC up and down scans of $30Na_2O\text{-}70P_2O_5$ glass using a heating cycle of 30-450 °C. A third upscan was taken to confirm the reproducibility of the T_g.

The T_g obtained for the $30Na_2O$-$70P_2O_5$ is lower than the value reported in the literature. This is most likely the result of the hygroscopic nature of the glass, which makes precisely determining the T_g challenging as it is affected by atmospheric water.[3] In line with inorganic glass nomenclature, water itself can be considered as a network modifier in the same sense as sodium oxide and depolymerises the glass network, reducing the T_g of ultraphosphate glasses.[4]

The lack of an endothermic peak in the first DSC upscan suggests that this water uptake is not a surface effect, but rather a structural one. The glass transition is reproducible in both upscans and on cooling, suggesting the integration of water within the structure. This is consistent with the small peak at 1644.80 cm^{-1} in the glass FTIR (**Figure S17a**).[5,6]

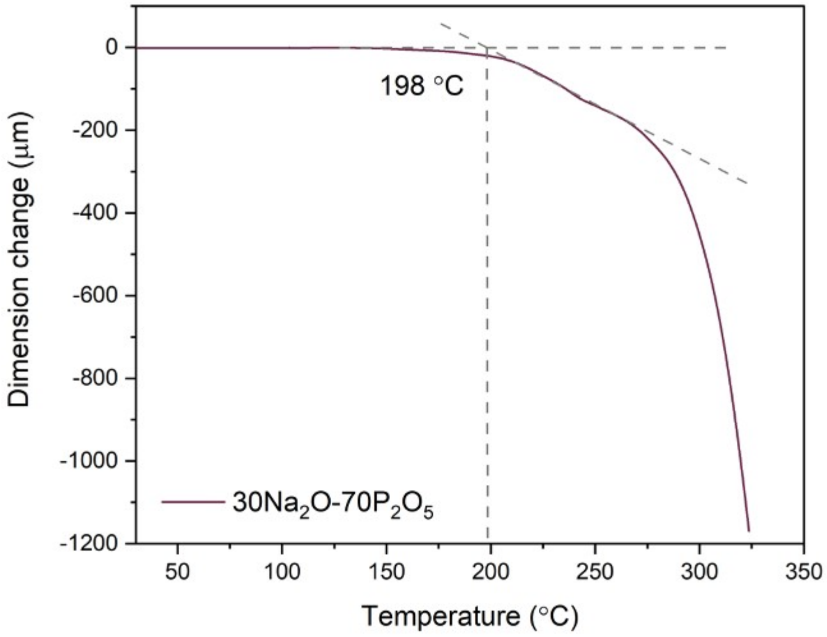

Figure S11. Thermomechanical analysis of $30Na_2O$-$70P_2O_5$ glass starting material, showing an inflection at 198 °C. A heating range of 30-250 °C was used.

2.4 Microscopy

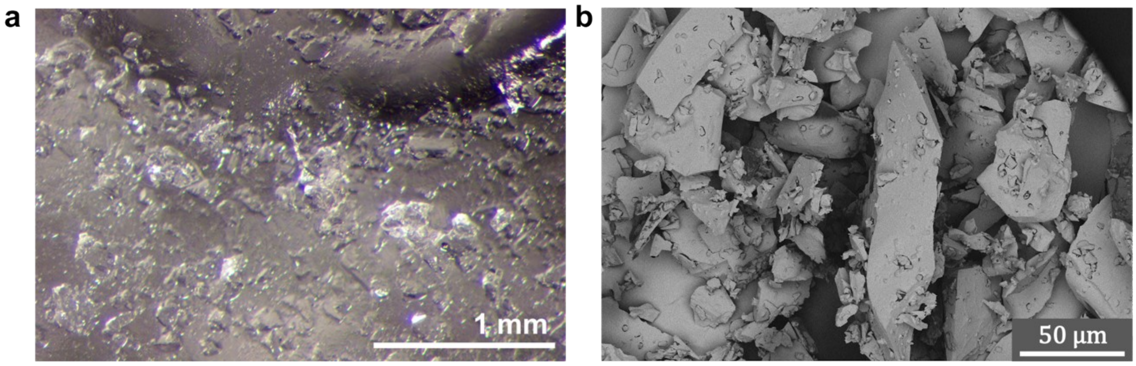

Figure S12 a. Optical images of bulk $30Na_2O$-$70P_2O_5$ glass and **b.** SEM image of powdered $30Na_2O$-$70P_2O_5$ in backscattered imaging mode.

3. Blend characterisation

3.1 PXRD analysis

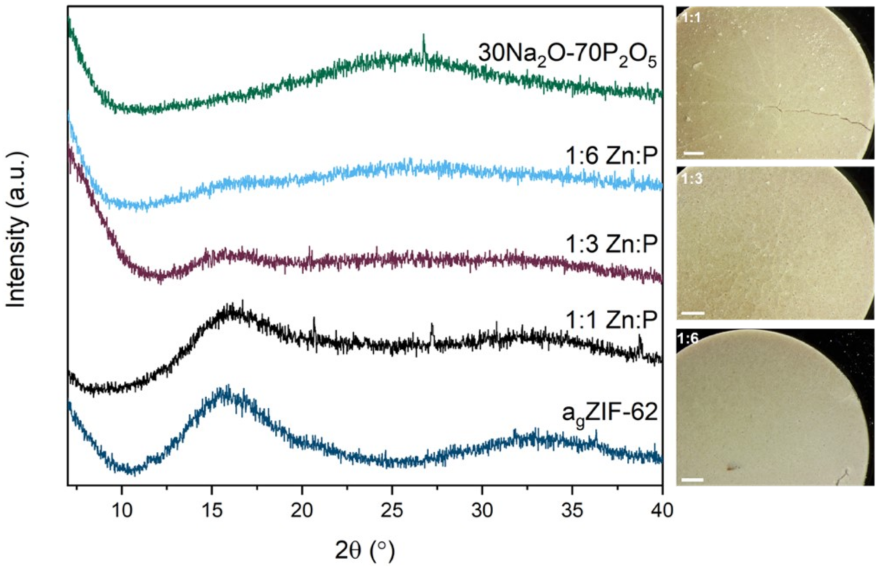

Figure S13. PXRD patterns of the physical mixtures of the three compositions, along with the starting materials. Optical images of the pelletised mixtures prior to heat treatment are shown on the right. Scale bar on all images is 1 mm.

3.2 ^{1}H NMR spectroscopy

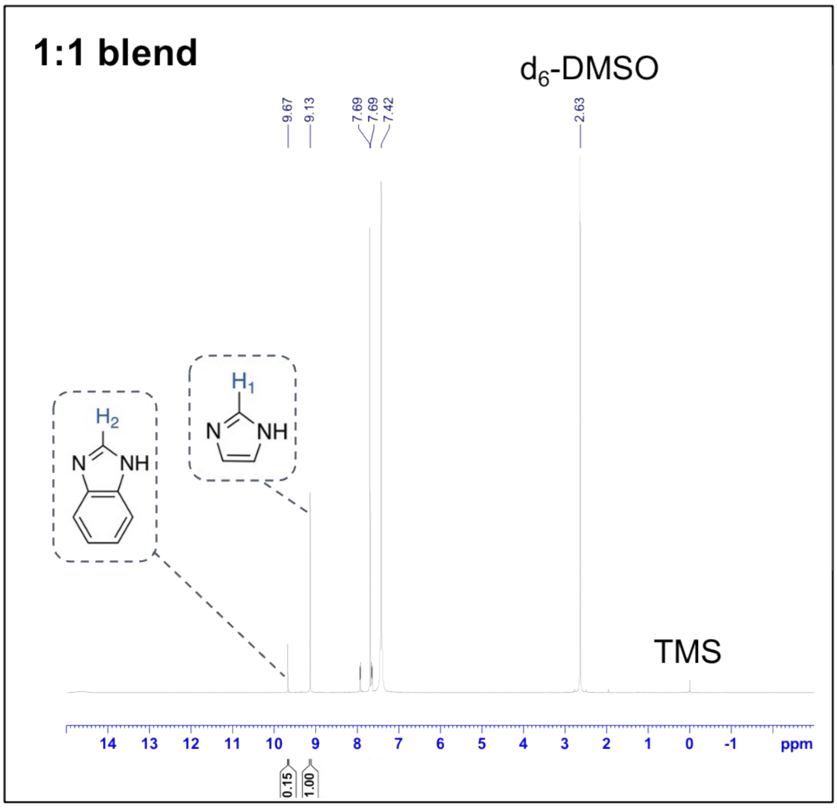

Figure S14. ^{1}H NMR spectrum of 1:1 blend. δ_H (500 MHz, DCl (35%)/D_2O: DMSO-d_6 (1:5), TMS) 9.67 (1H, s, H_2), 9.13 (1H, s, H_1), 7.69 (5H, m, aromatic), 2.63(DMSO), 0.00 (TMS). Peak at 7.94 ppm is likely a solvent peak, consistent with literature spectra on a_gZIF-62 in the same solvent.[1] Peak 7.42 ppm is also likely a solvent peak.

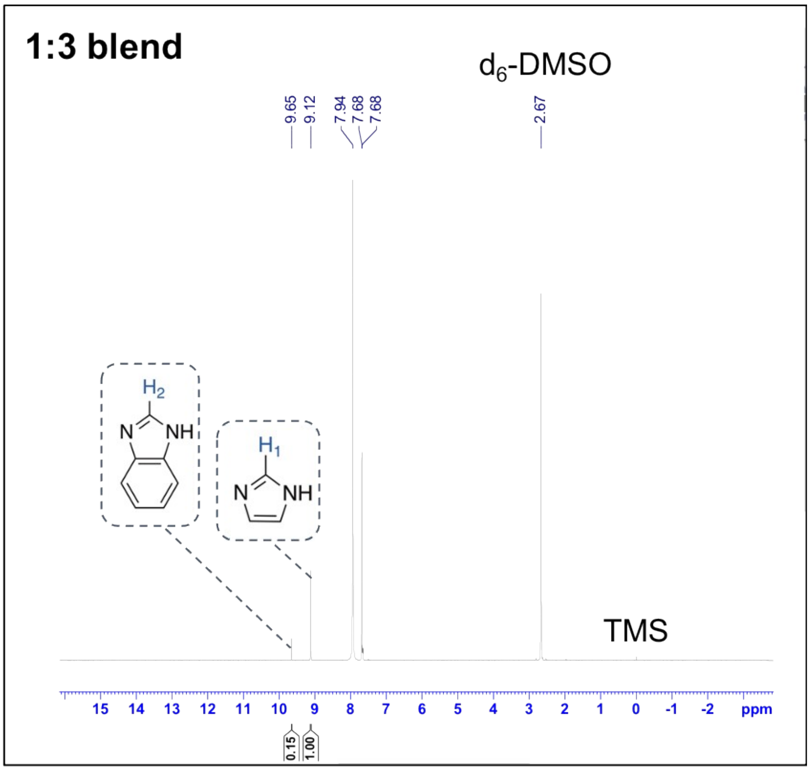

Figure S15. ^{1}H NMR spectrum of 1:3 blend. δ_H (500 MHz, DCl (35%)/D_2O: DMSO-d_6 (1:5), TMS) 9.65 (1H, s, H_2), 9.12 (1H, s, H_1), 7.68 (5H, m, aromatic), 2.63(DMSO), 0.00 (TMS). Peak at 7.94 ppm is likely a solvent peak, consistent with literature spectra on a_gZIF-62 in the same solvent.[1]

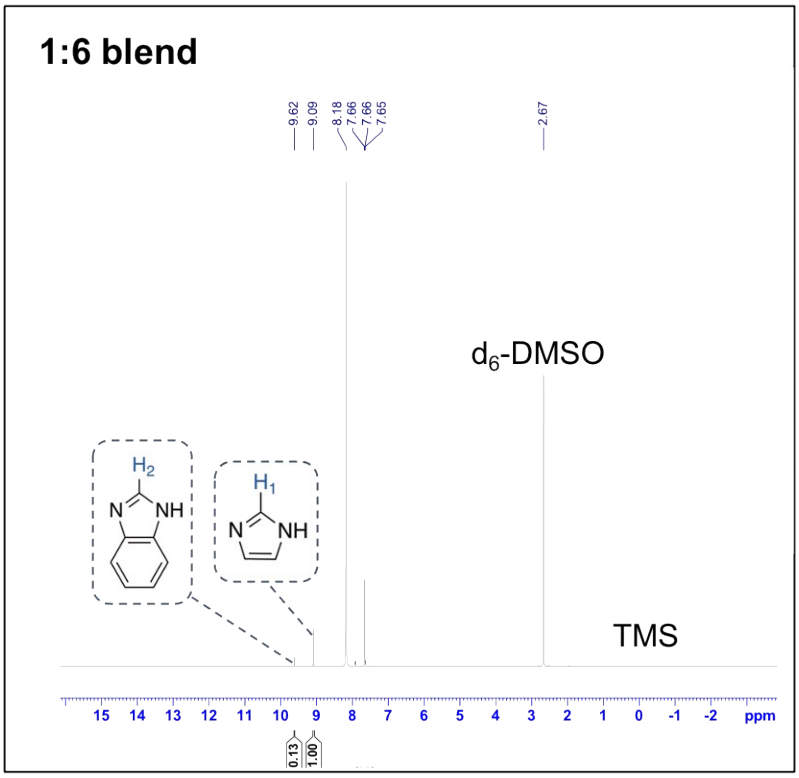

Figure S16. ^{1}H NMR spectrum of 1:6 blend. δ_H (500 MHz, DCl (35%)/D_2O: DMSO-d_6 (1:5), TMS) 9.62 (1H, s, H_2), 9.09 (1H, s, H_1), 7.66 (5H, m, aromatic), 2.67 (DMSO), 0.00 (TMS). Peak at 7.92 ppm is likely a solvent peak, consistent with literature spectra on a_gZIF-62 in the same solvent.[1] Peak at 8.18 ppm likely a solvent peak and the TMS peak is very small but present at 0 ppm.

3.3 FTIR spectroscopy

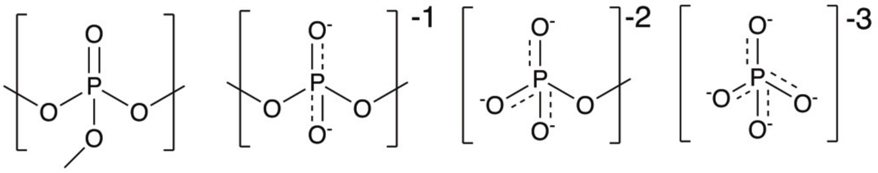

Q^3 Q^2 Q^1 Q^0

Key bands in the $30Na_2O$-$70P_2O_5$ FTIR spectrum are at 1318.48 (P=O bond in Q^3 unit), 912.63 (P-O-P asymmetric stretch), 744.19 (P-O-P symmetric stretch) and 528.72 and 586.55 cm^{-1} (both associated with P-O-P bending in and Q^2 and Q^3 units).[5,6] A small band at 1644.80 cm^{-1} is also evident, showing that P-OH bonds exist within the glass structure from the incorporation of water.[5,6]

Similarly, FTIR spectrum of a_gZIF-62 agrees with literature spectra. A sharp peak at 669 cm^{-1} is associated with C-H stretching in benzimidazole,[7] 1086 and 1320 cm^{-1} C-N stretching[8] and C-H stretch[7] at 3110 cm^{-1}.

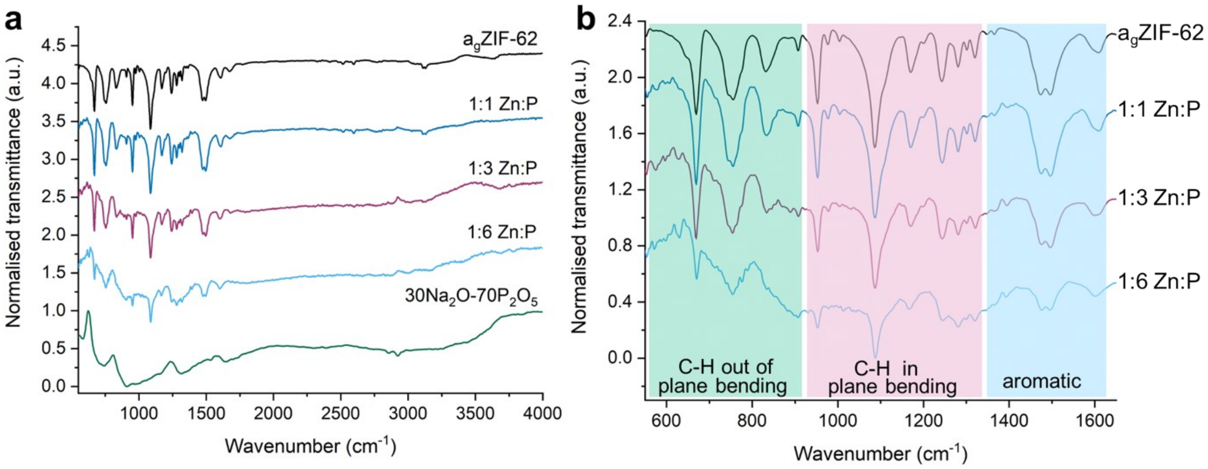

Figure S17. a. ATR-FTIR spectroscopy of all three blends and both starting materials and **b.** Zoomed in range of blends and a_gZIF-62.

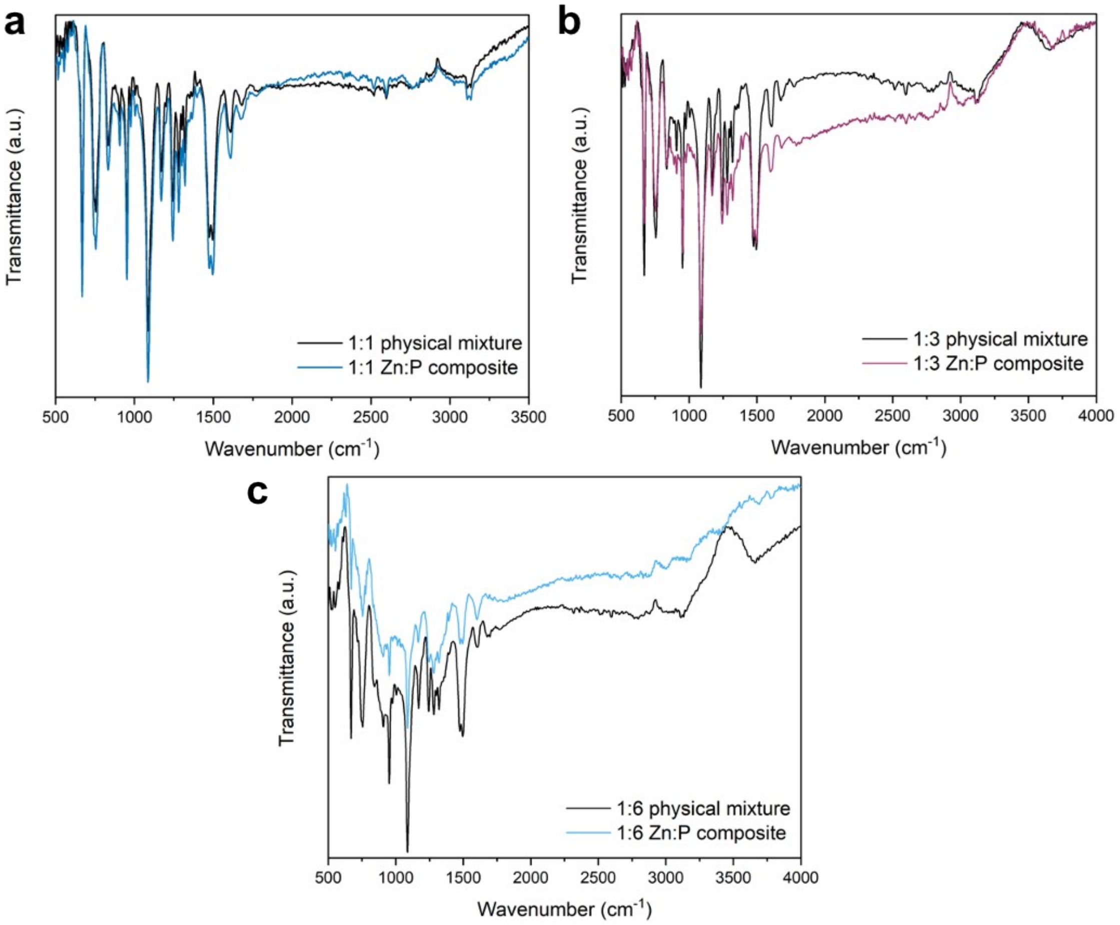

Figure S18. ATR-FTIR spectroscopy comparison of **a.** 1:1 physical mixture vs blend, **b.** 1:3 physical mixture vs blend and **c.** 1:6 physical mixture vs blend.

3.4 Raman spectroscopy

Two distinct bands are present in the Raman spectrum of $30Na_2O\text{-}70P_2O_5$ at 669 and 1153 cm^{-1} corresponding to symmetrical P-O-P stretching and symmetric PO_2 stretching in the Q^2 tetrahedra respectively.[3,9] The P=O stretching frequency would be expected above 1200 cm^{-1}.

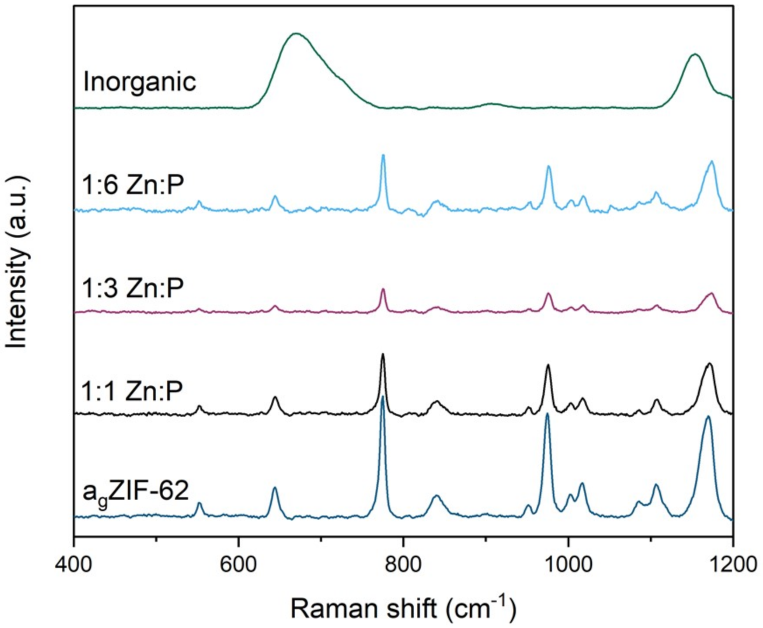

Figure S19. Raman spectra of the three blend samples and starting materials.

3.5 SEM-EDS analysis

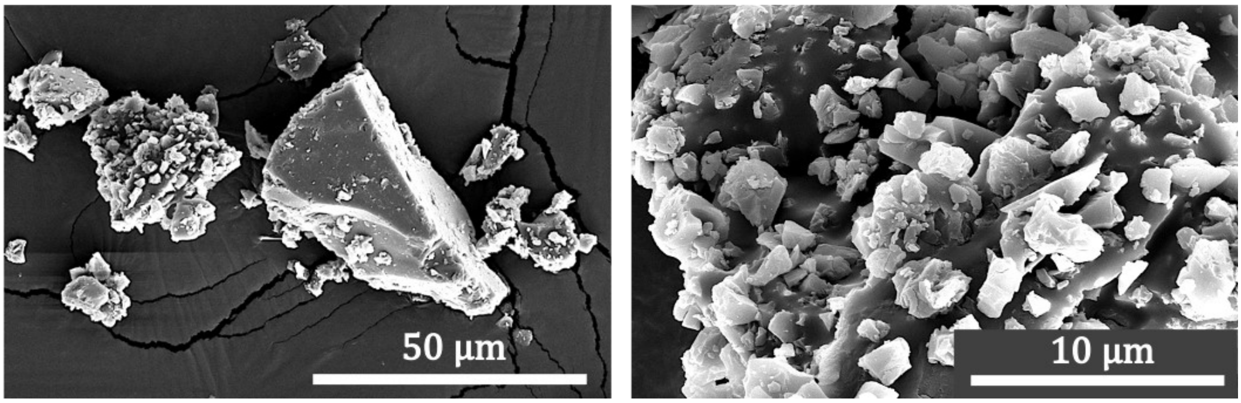

Figure S20. Secondary electron SEM images of 1:1 physical mixture, indicating a lack of flow prior to heat treatment.

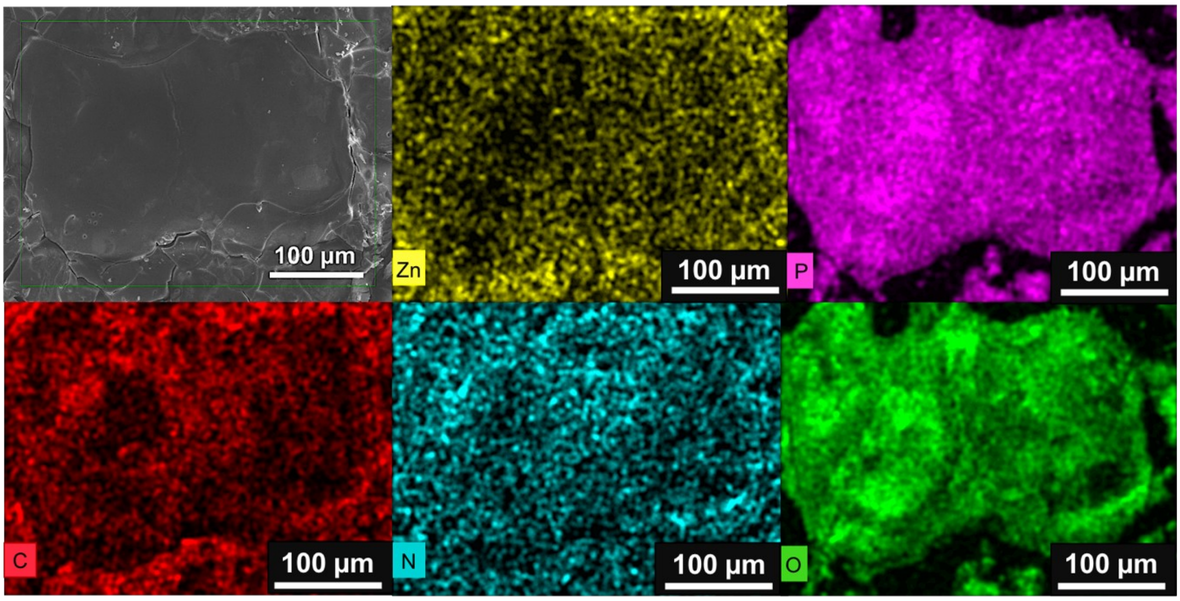

Figure S21. SEM-EDS elemental mapping of the 1:1 Zn:P blend, where scale bar is 100 µm in all images. Yellow, pink, green, red and blue maps represent zinc, phosphorous, oxygen, carbon and nitrogen respectively.

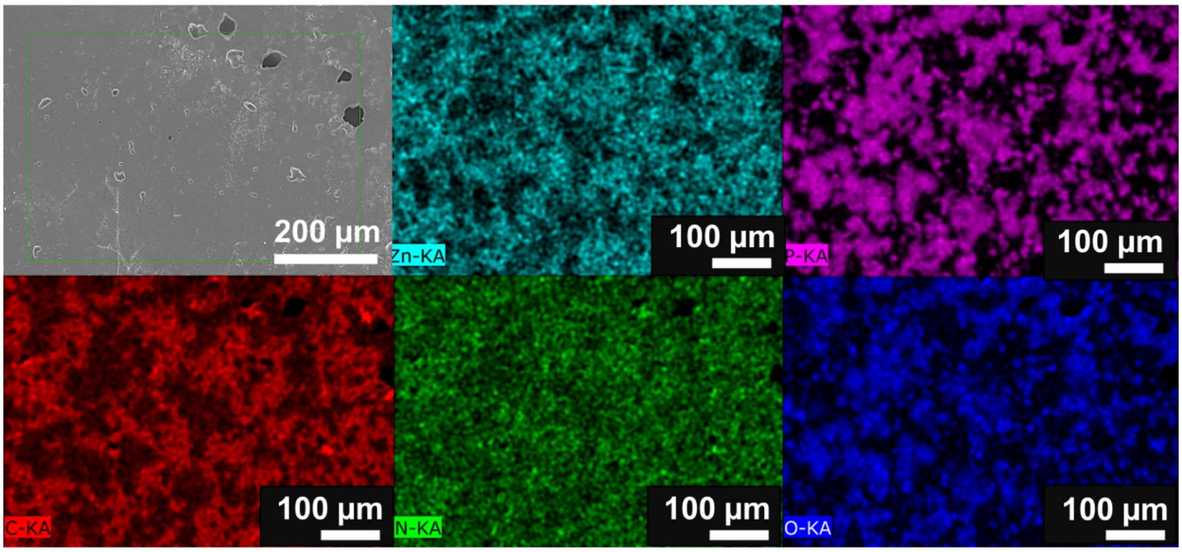

Figure S22. SEM-EDS elemental mapping of the 1:3 Zn:P blend. Pink, blue, dark blue, red and green represent phosphorous, zinc, oxygen, carbon and nitrogen respectively.

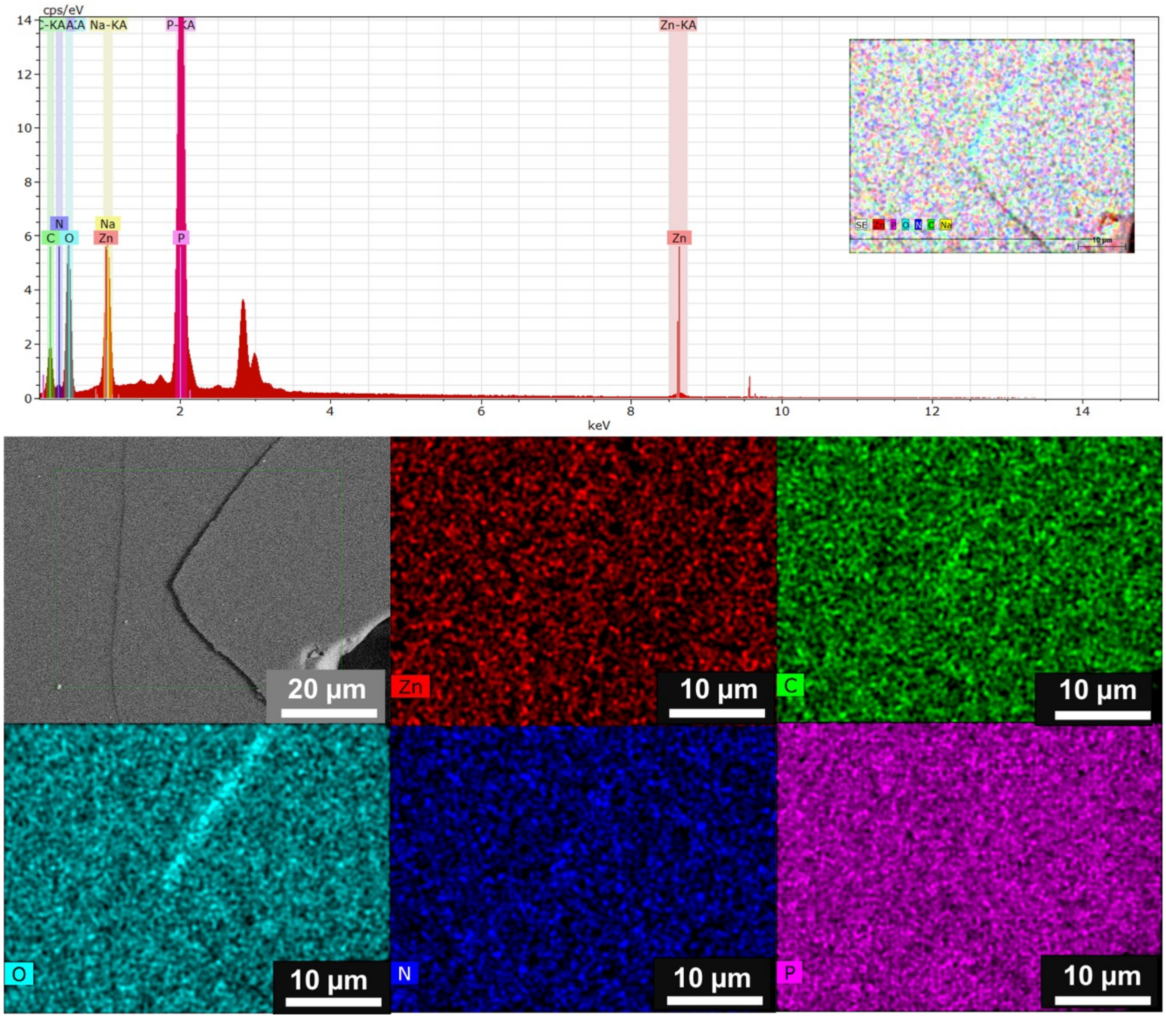

Figure S23. SEM-EDS elemental mapping of the 1:6 Zn:P blend. Cyan, pink, blue, red and green represent oxygen, phosphorous, nitrogen, zinc and carbon respectively.

3.6 Scanning transmission electron microscopy (STEM) and STEM-EDS

Initial image processing was carried out using the HyperSpy[10] open-source Python package. Tilt-series images were first aligned using cross-correlation routines in the Numpy and SciKit-Image Python libraries. Tilt-axis alignment was carried out by manual refinement together with filtered back-projection reconstructions implemented in SciKit-Image. Tomographic reconstructions were performed using a compressed sensing (CS) algorithm implemented in Python.[11,12] Briefly, this code implements a non-negative projector using the ASTRA Toolbox[13] together with total variation (TV) based regularisation in a primal-dual hybrid gradient algorithm.[14] First order TV regularisation was used in all reconstructions, accounting for material phases with homogeneous density between interfaces. Reconstructions were carried out on ARC3 nodes equipped with Nvidia P100 GPU cards, part of the High-Performance Computing facilities at the University of Leeds.

ADF-STEM tomography enables three-dimensional atomic number Z contrast.[15] Given information about the multi-component formation process and EDS spectra showing P and O signals (attributed uniquely to the inorganic glass) and Zn, C, and N signals (attributed uniquely to a_gZIF-62), tomographic reconstruction volumes were inspected for intensity distributions showing two distinct average atomic number densities within the particle volume. An edge spread function (ESF) approach implemented in HyperSpy was used to identify distinct intensity phases, following the methods outlined in work by Yuan[16] and Collins.[17] Briefly, the volume retained after intensity-based thresholding was calculated for a series of threshold values spanning the intensity range. Sigmoid inflections in the resulting curves, modelled by the ESF,[18,19] indicate phase boundaries. Here, we used the ESF approach to identify candidate threshold ranges for particle/exterior and inorganic glass/a_gZIF-62 phase boundaries. The exact thresholds were then further refined by manual adjustment in ImageJ to ensure the particle/exterior interfaces did not exclude sample material. After the application of thresholds, additional extraneous intensity outside the particle due to residual reconstruction artefacts were removed from the volume ImageJ.

The final segmented volumes were visualised in Paraview (Kitware) as isosurface renderings. EDS maps were generated in Esprit software (Bruker) for P, O, Zn, C and N *K* lines. Due to the overlap of the Na *K* and Zn *L* lines no maps were generated for Na. EDS maps provided validation for the 3D segmentation results from ADF-STEM, with unique single-phase features visible in EDS maps matched to those visualised in 3D by segmentation of ADF-STEM tomography.

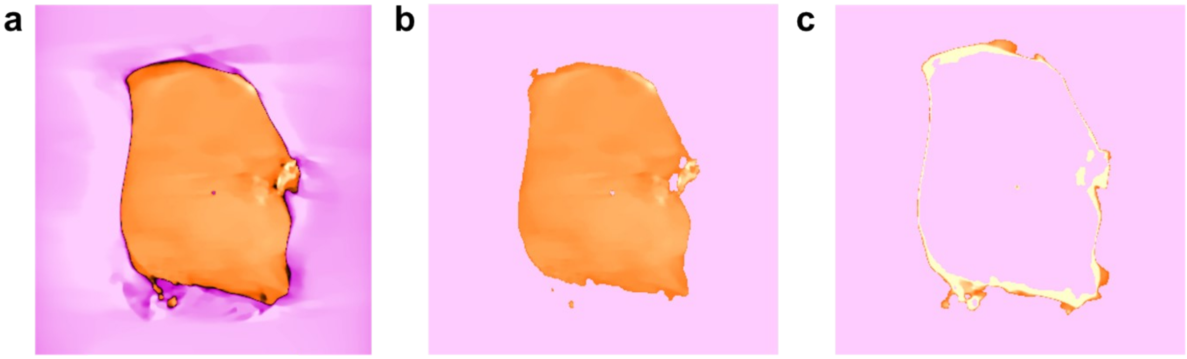

Figure S24. a. Cross section of the 1:3 blend particle, **b.** Inner structure/phase after separation/thresholding and **c.** Outer structure/phase after separation/thresholding, showing how the two phases were separated.

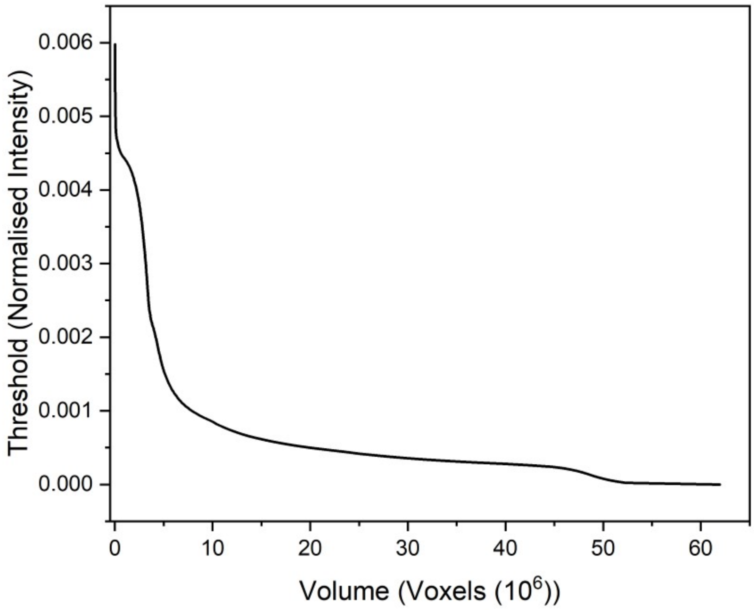

Figure S25. Edge spread function of the 1:3 blend sample.

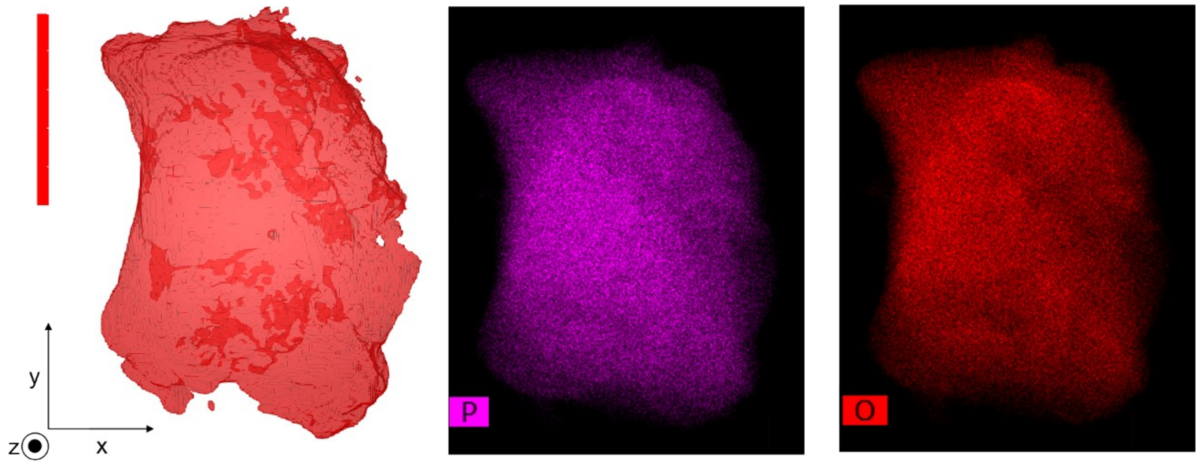

Figure S26. 2D EDS elemental maps of oxygen and phosphorous that comprise the inorganic phase of a particle of the intermediate 1:3 blend sample.

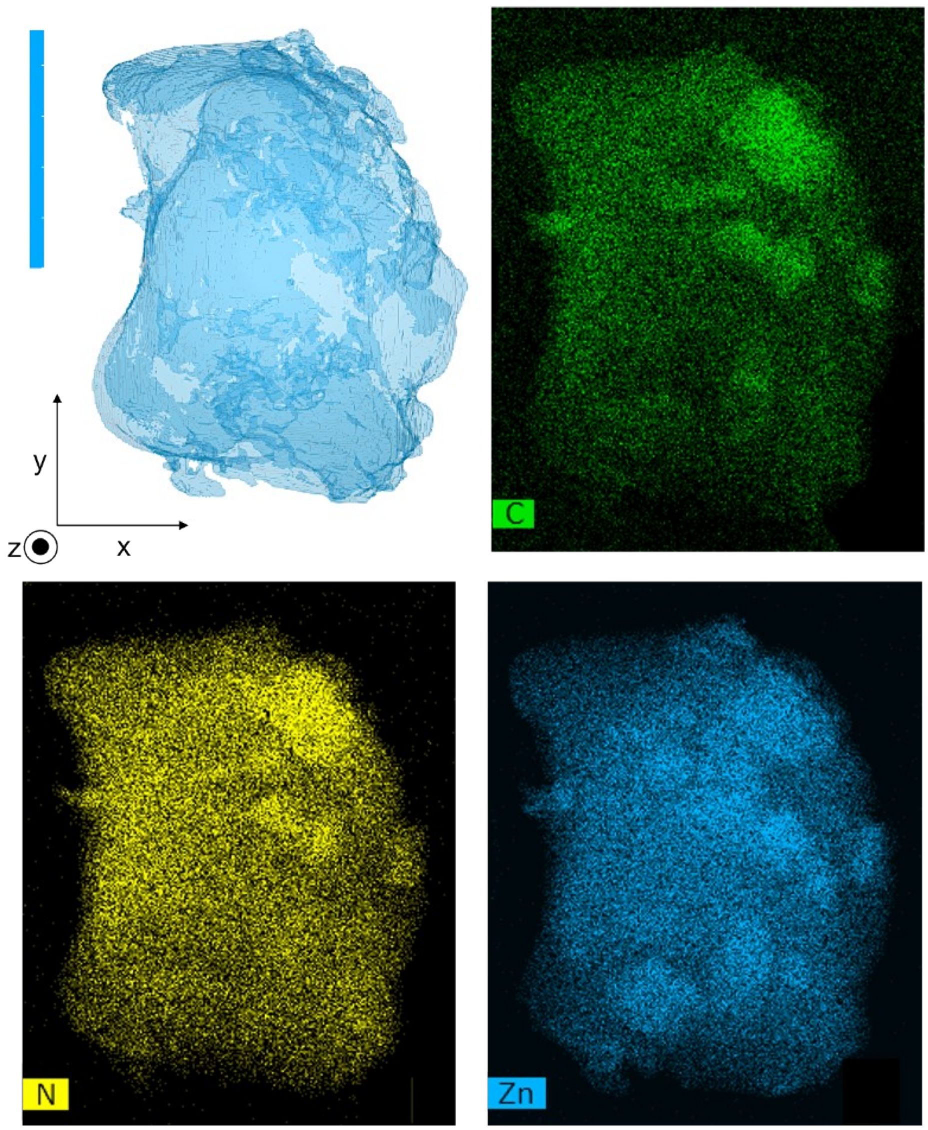

Figure S27. 2D EDS elemental maps of carbon, nitrogen and zinc that comprise the a_gZIF-62 phase of a particle of the intermediate 1:3 blend sample. The Zn K line was used to produce the Zn elemental map.

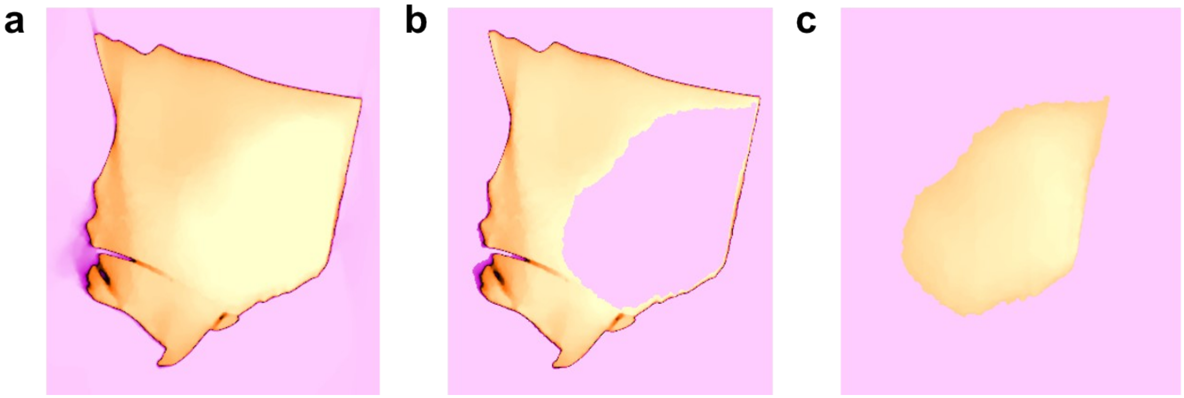

Figure S28. a. Cross section of the 1:1 blend particle showing a difference in intensity, **b.** Outer structure after separation/thresholding and **c.** Inner structure after separation/thresholding, showing how the two phases were separated.

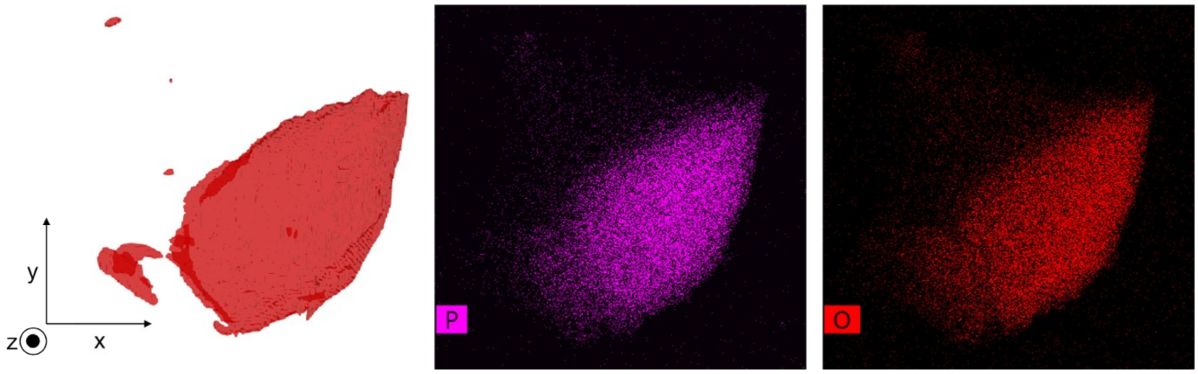

Figure S29. 2D EDS elemental maps of oxygen and phosphorous that comprise the inorganic phase of a particle of the 1:1 blend sample.

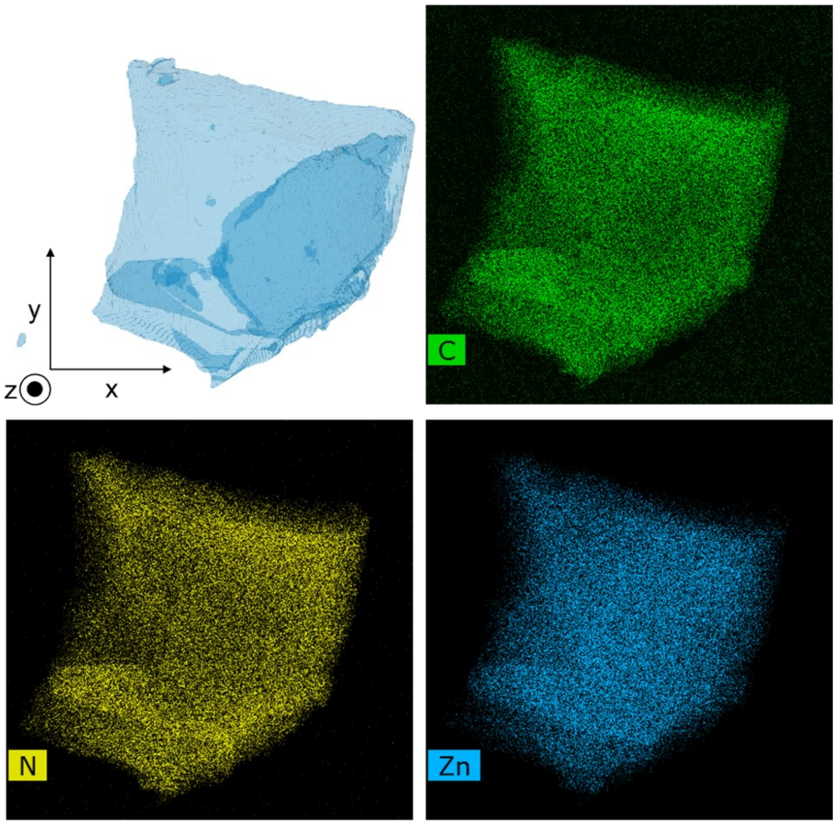

Figure S30. 2D EDS elemental maps of carbon, nitrogen and zinc that comprise the a_gZIF-62 phase of a particle of the 1:1 blend sample. The Zn K line was used to produce the Zn elemental map.

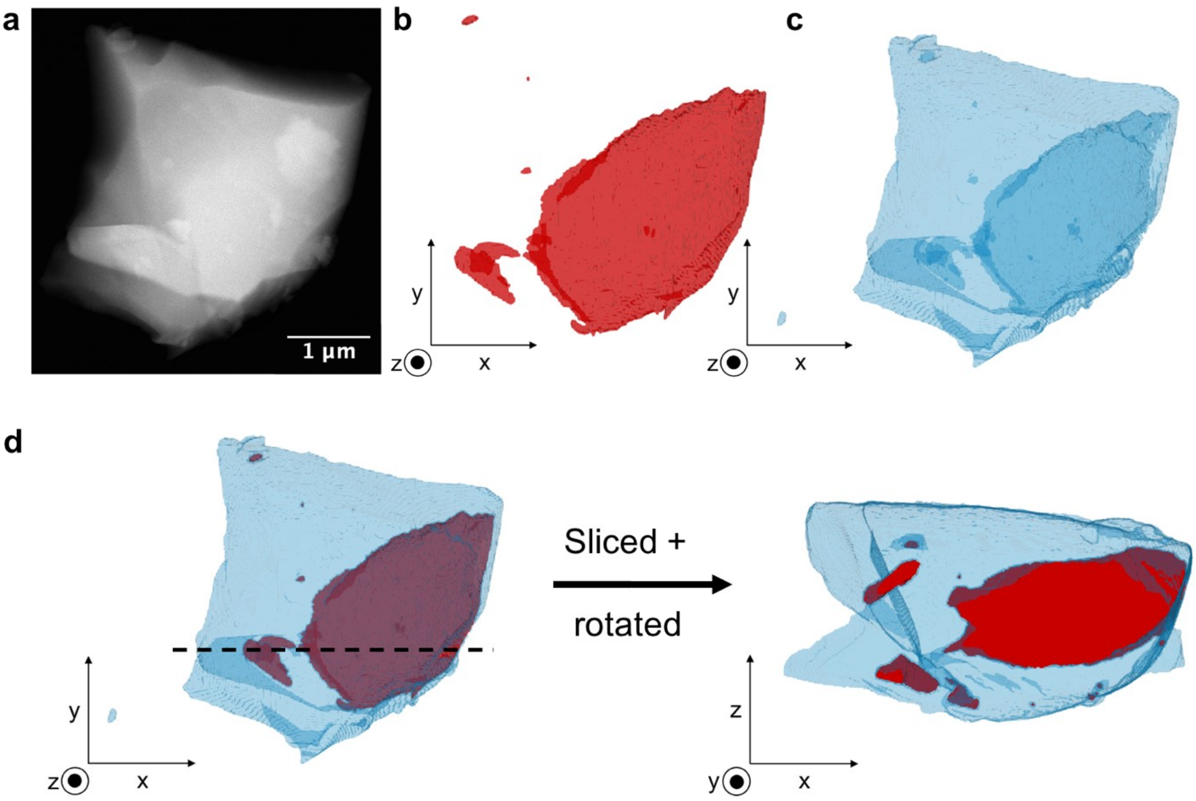

Figure S31. a. Annular dark field STEM (ADF-STEM) image of the 1:1 blend, **b.** ADF-STEM tomography of the inorganic glass phase of the grain, **c.** ADF-STEM tomography of the corresponding $a_gZIF\text{-}62$ phase of the particle and **d.** Combined phases of the studied particle, showing close mixing of the individual phases.

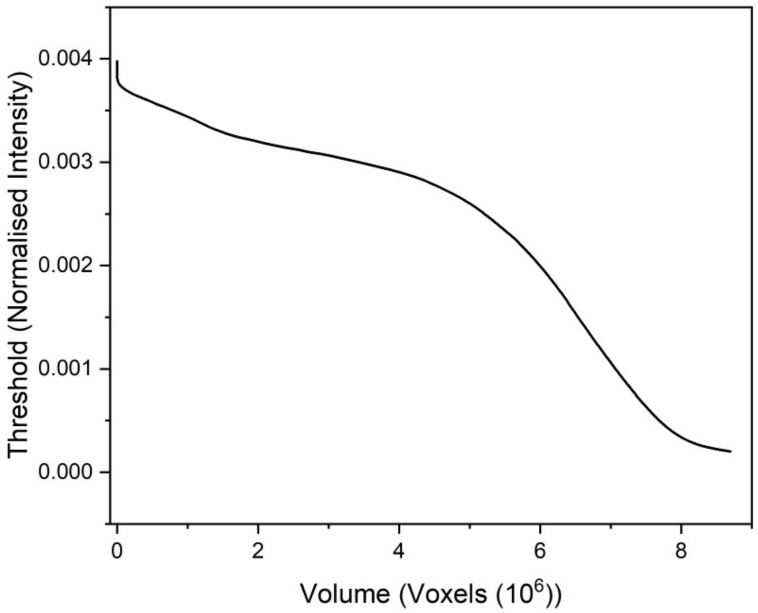

Figure S32. Edge spread function of the 1:1 blend sample.

3.7 Thermal analysis

Very minor mass loss was observed for all three physical mixtures at the working temperature for blend synthesis (400 °C). The T_w selected was a compromise between promoting liquid phase mixing at a temperature sufficiently higher than the T_g of a_gZIF-62, given its high viscosity at T_g, and avoiding minor decomposition of the physical mixtures during blend formation.

Results from FTIR, ^{1}H NMR, PXRD and Raman indicate negligible decomposition of the blends and thus this T_w was selected.

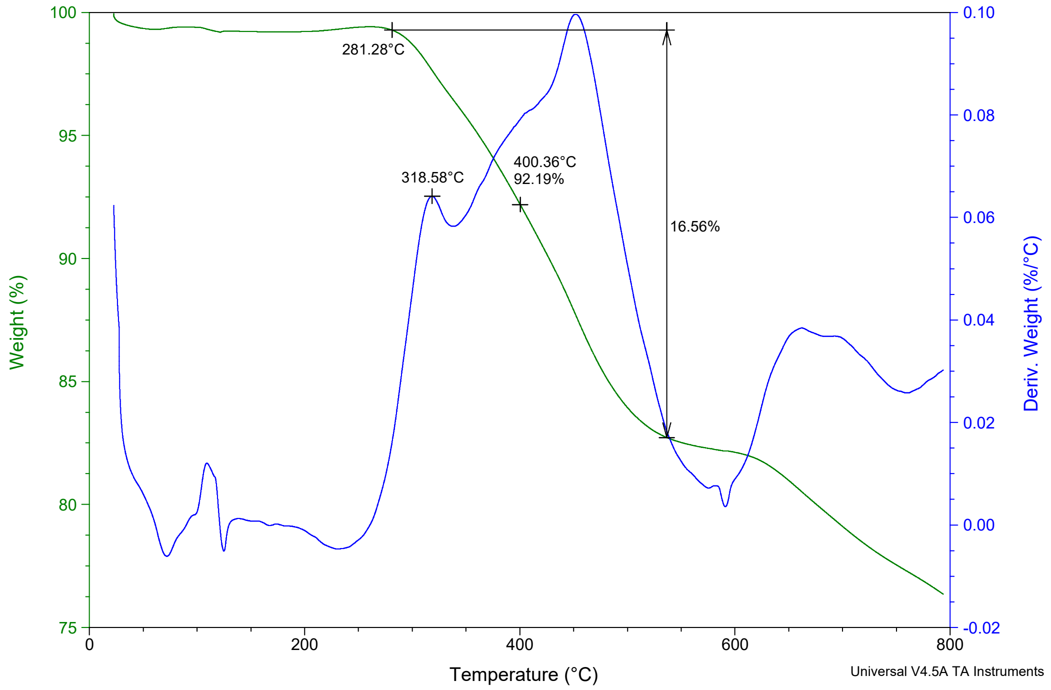

Figure S33. TGA trace of the 1:1 physical mixture, showing the change in weight and derivative of the weight curve with respect to temperature.

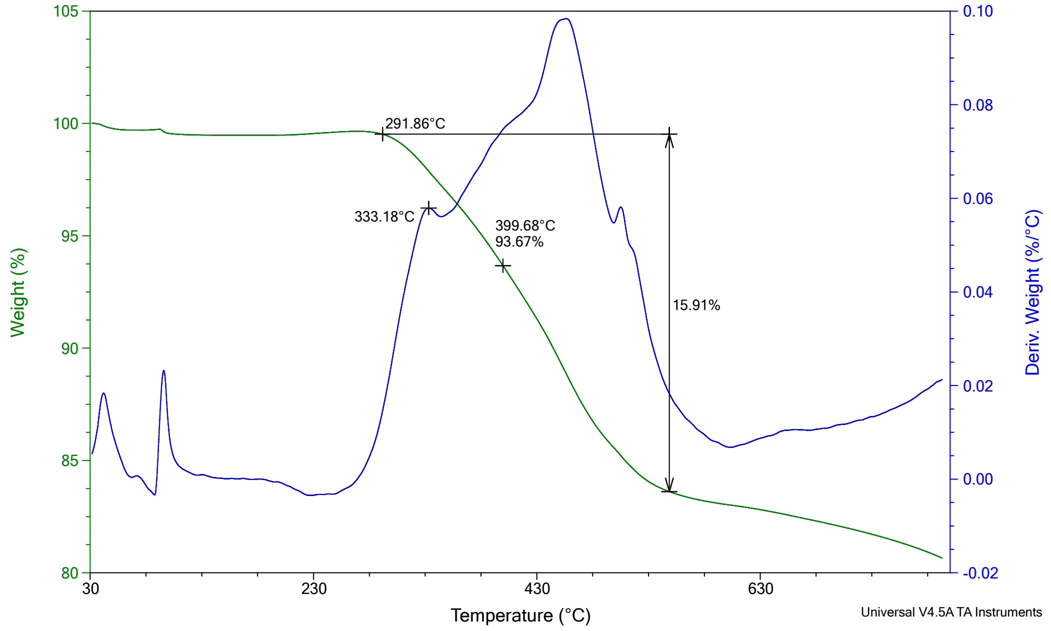

Figure S34. TGA trace of the 1:3 physical mixture, showing the change in weight and derivative of the weight curve with respect to temperature.

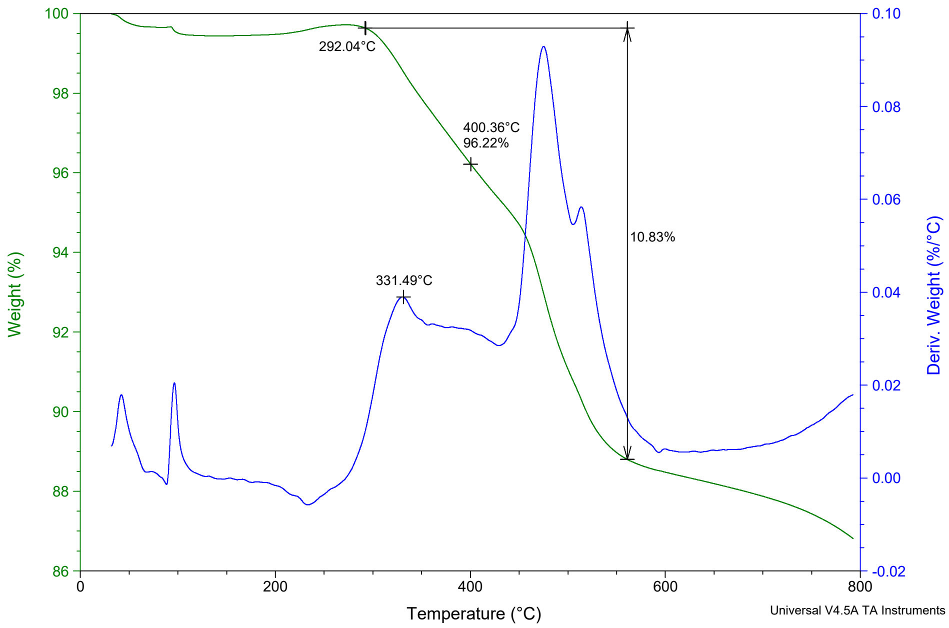

Figure S35. TGA trace of the 1:6 physical mixture, showing the change in weight and derivative of the weight curve with respect to temperature.

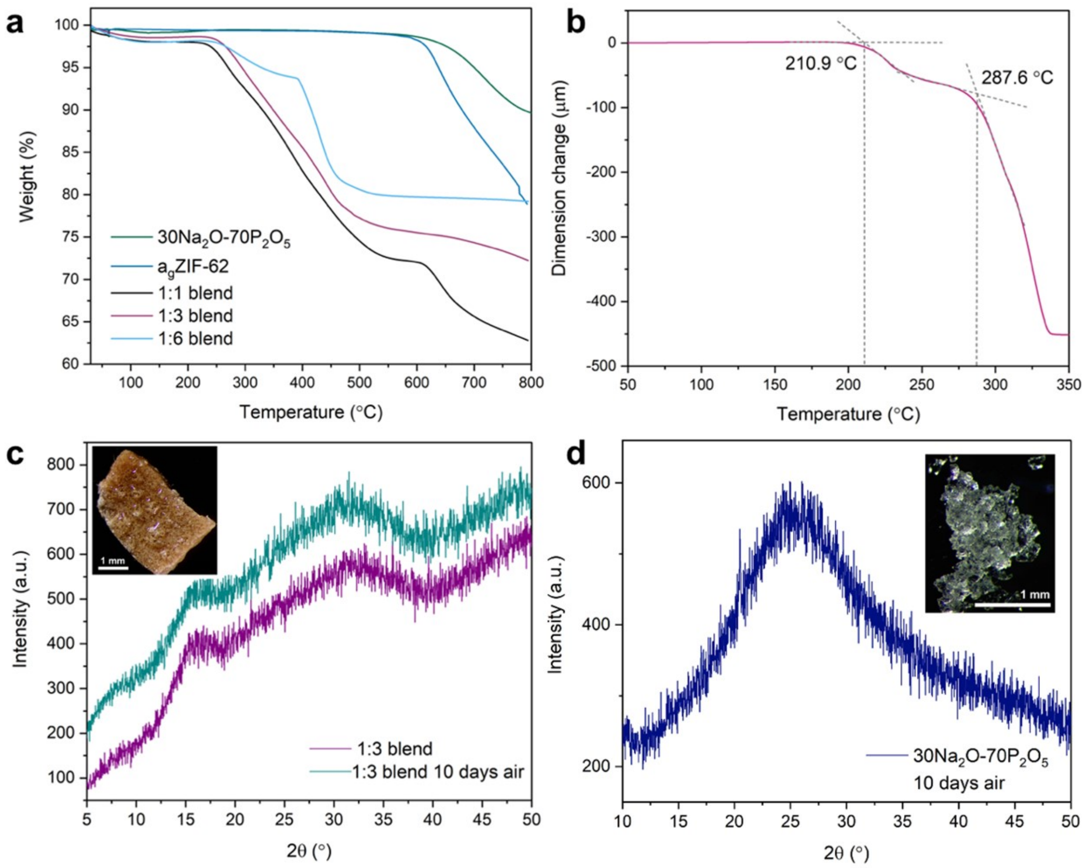

Figure S36. a. TGA profiles of all three blends and the pristine starting materials using a heating rate of 10 °C min^{-1} and **b.** TMA curve of the 1:6 sample to confirm the T_g value obtained from DSC. A heating range of 30-350 °C was used for the TMA analysis, **c.** PXRD of the 1:3 blend and 1:3 blend after 10 days air exposure with inset showing the blend after air exposure and **d.** PXRD of the pristine inorganic glass after 10 days air exposure with inset showing the gel-like sample formed from the air exposure. Scale bar on both insets is 1 mm.

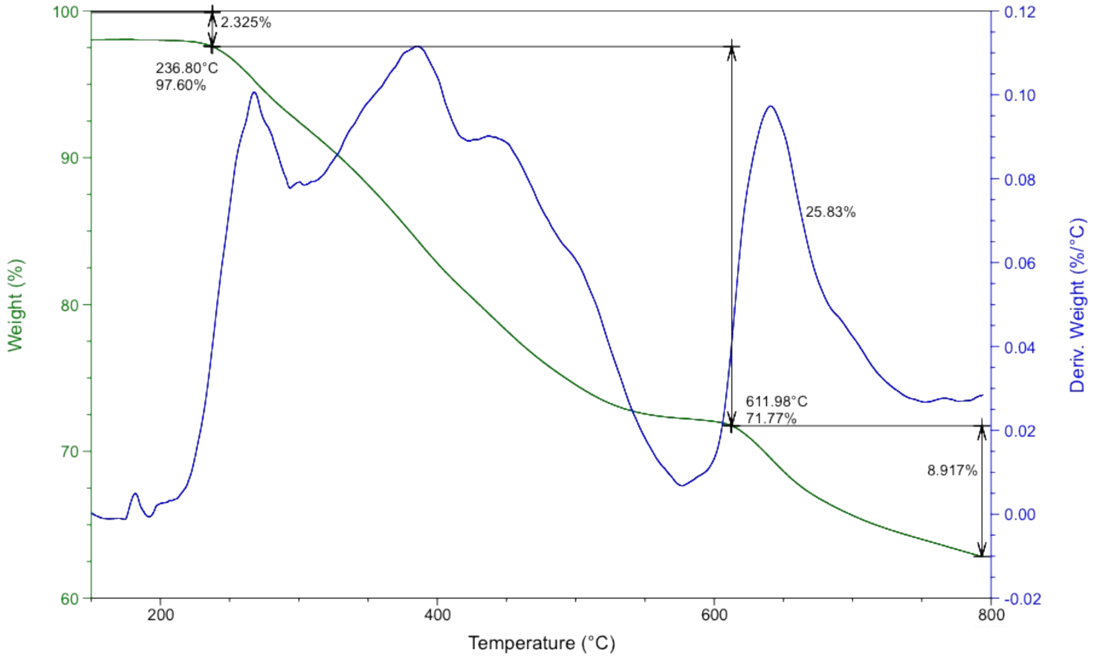

Figure S37. TGA trace of the 1:1 blend, showing the change in weight and derivative of the weight curve with respect to temperature.

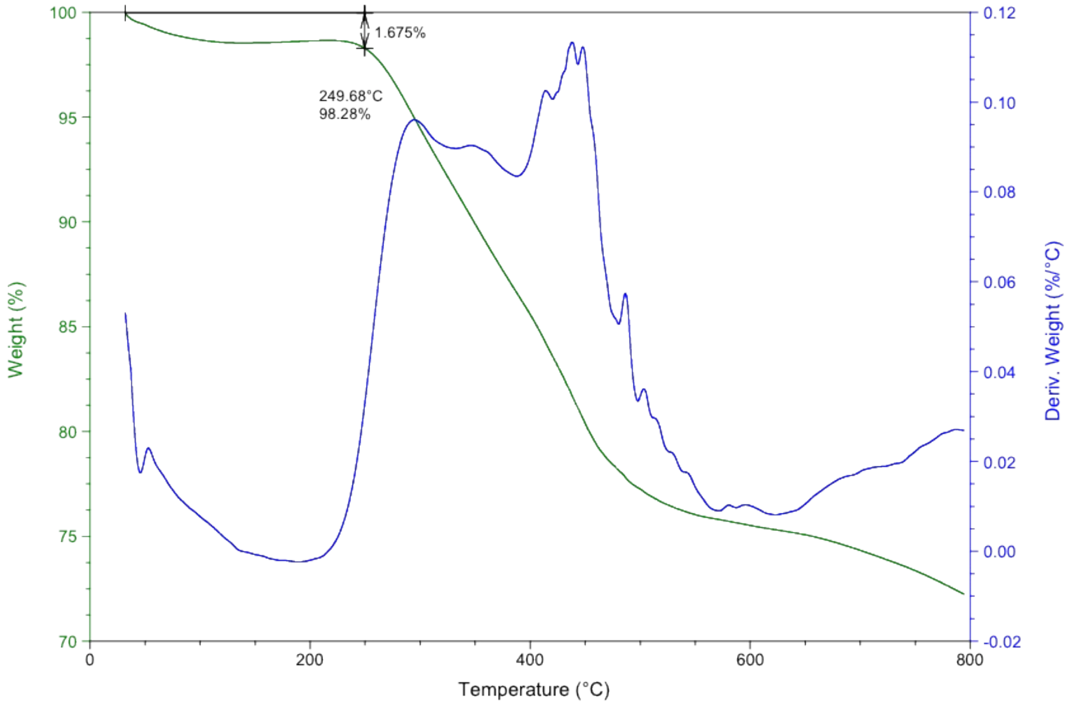

Figure S38. TGA trace of the 1:3 blend, showing the change in weight and derivative of the weight curve with respect to temperature.

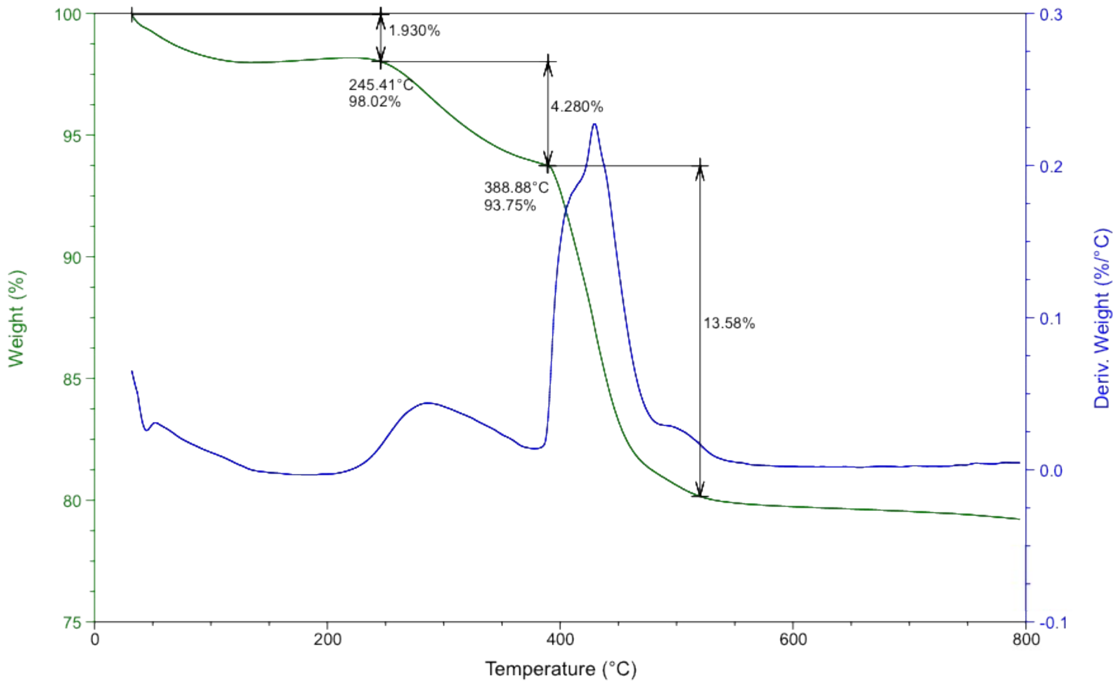

Figure S39. TGA trace of the 1:6 blend, showing the change in weight and derivative of the weight curve with respect to temperature.

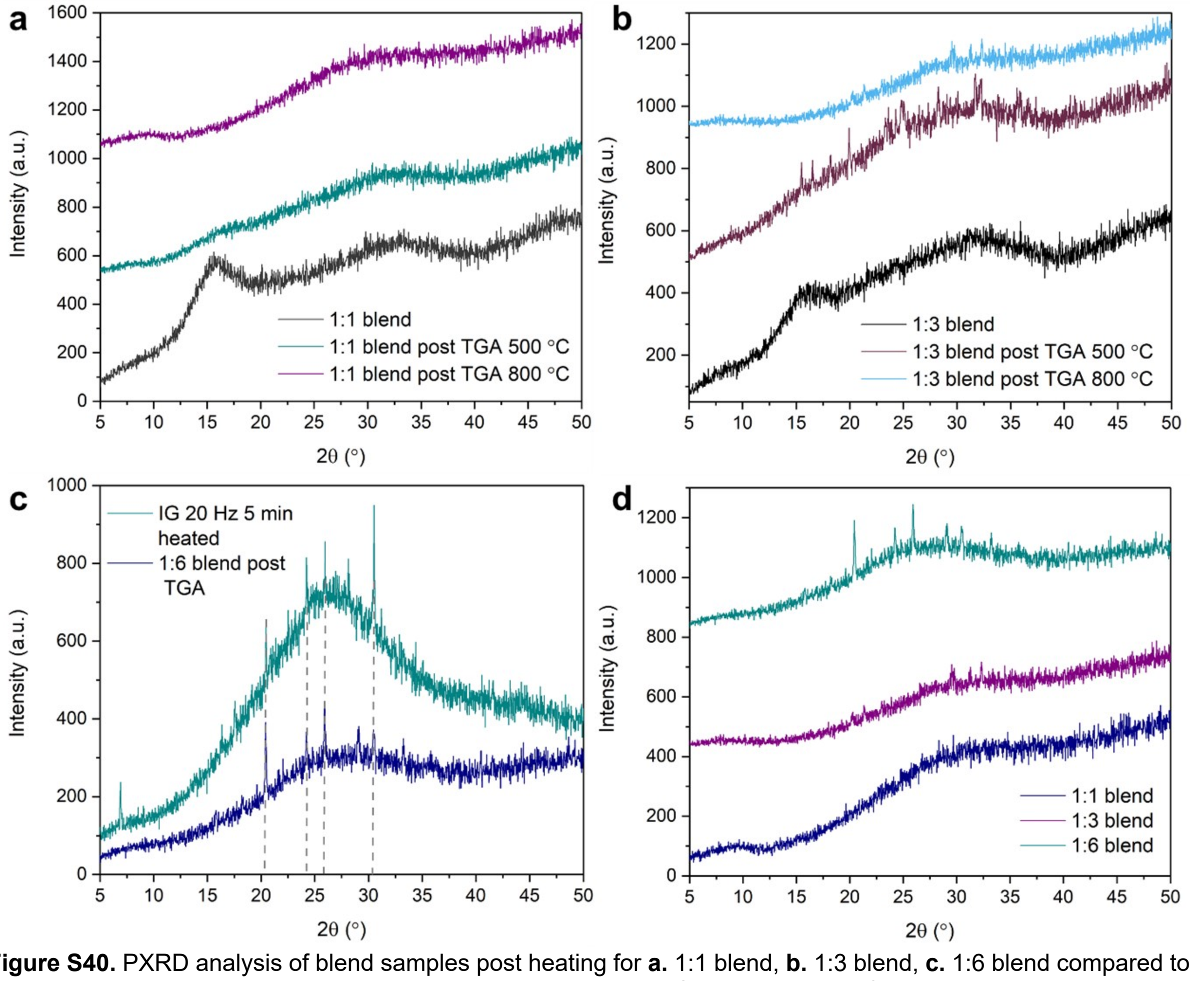

Figure S40. PXRD analysis of blend samples post heating for **a.** 1:1 blend, **b.** 1:3 blend, **c.** 1:6 blend compared to the ball milled, pelletised and heat-treated inorganic glass and **d.** Comparison plot of all blends post heating to 800 °C.

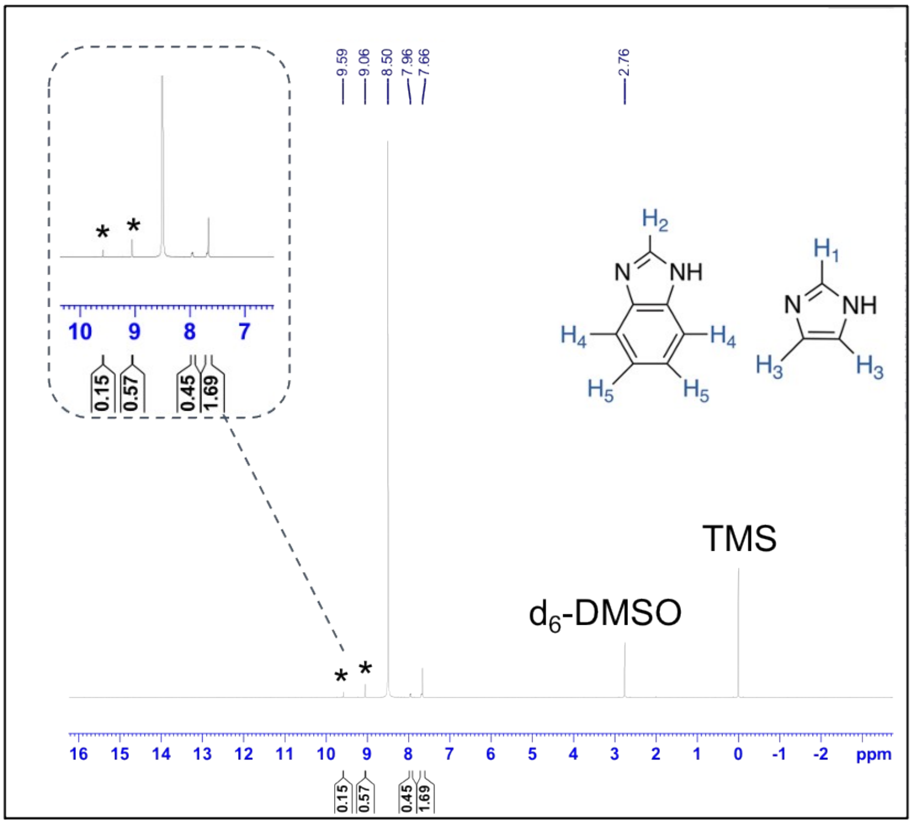

Figure S41. ^{1}H NMR of the 1:3 blend after heating to 500 °C. δ_H (500 MHz, DCl (35%)/D_2O: DMSO-d_6 (1:5), TMS) 9.59 (1H, s, H_2), 9.06 (1H, s, H_1), 7.66 (5H, m, aromatic), 2.76(DMSO), 0.00 (TMS). Peak at 8.50 ppm is likely a solvent peak, close to literature spectra on a_gZIF-62 in the same solvent.[1] Unidentified peak at 7.96 ppm. Asterisks represent the proton signals used to assess the integrity of the agZIF-62 framework.

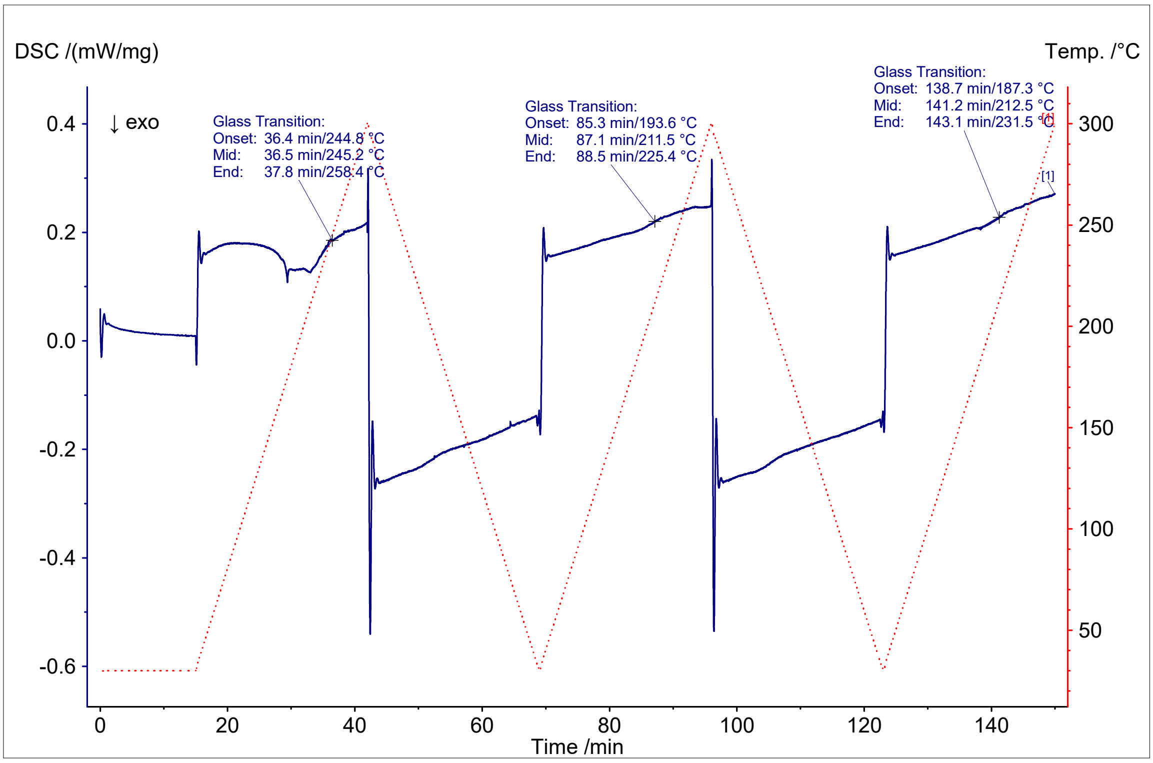

Figure S42. Full DSC up and down scans of the 1:1 physical mixture using a heating cycle of 30-300 °C.

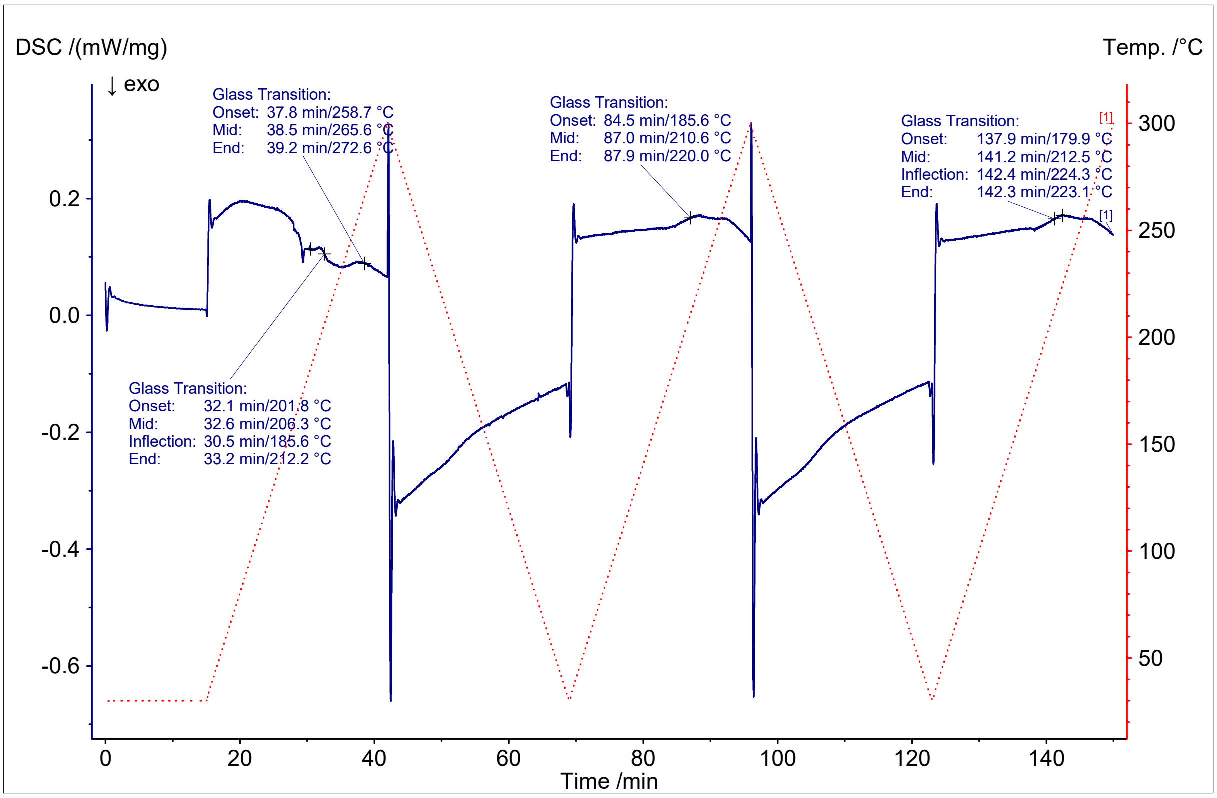

Figure S43. Full DSC up and down scans of the 1:3 physical mixture using a heating cycle of 30-300 °C.

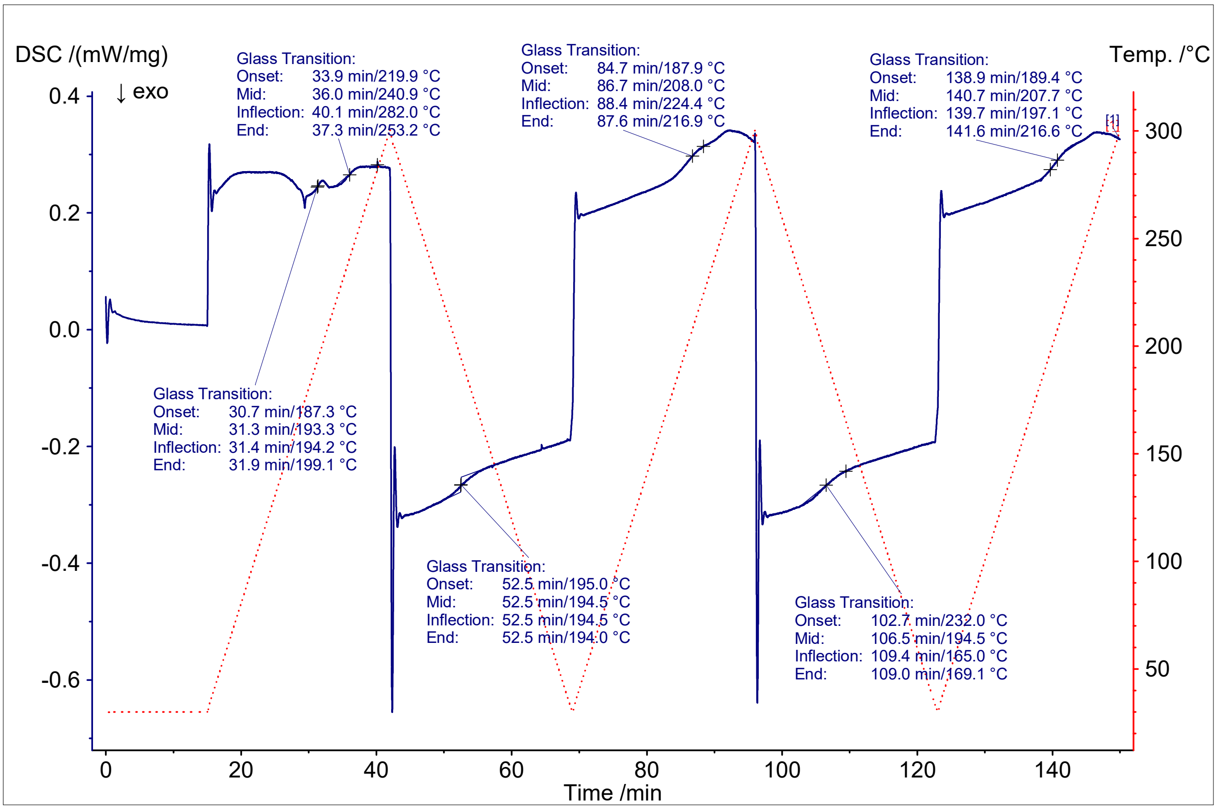

Figure S44. Full DSC up and down scans of the 1:6 physical mixture using a heating cycle of 30-300 °C.

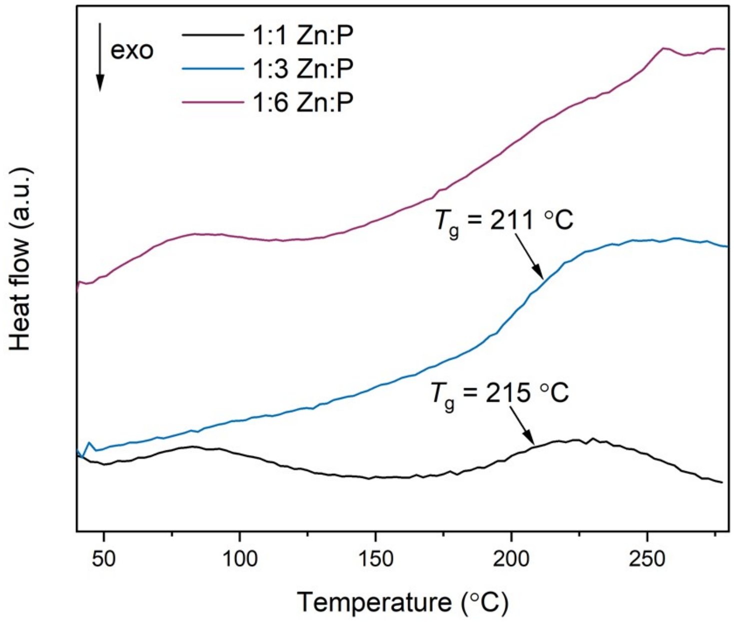

Figure S45. First DSC upscans of all three blends and the starting materials using a heating and cooling rate of 10 °C min^{-1}.

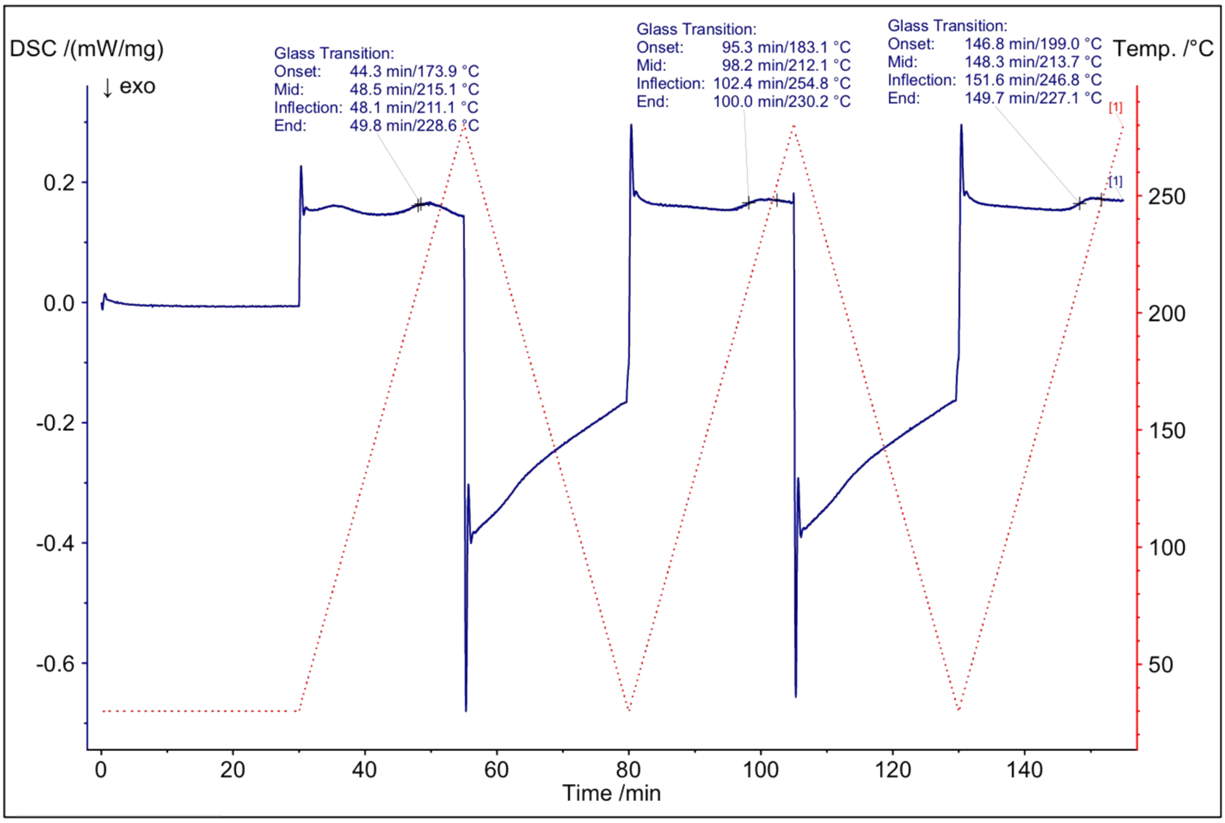

Figure S46. Full DSC up and down scans of the 1:1 blend using a heating and cooling rate of 10 °C min^{-1}. A third upscan was taken to confirm the reproducibility of the T_g.

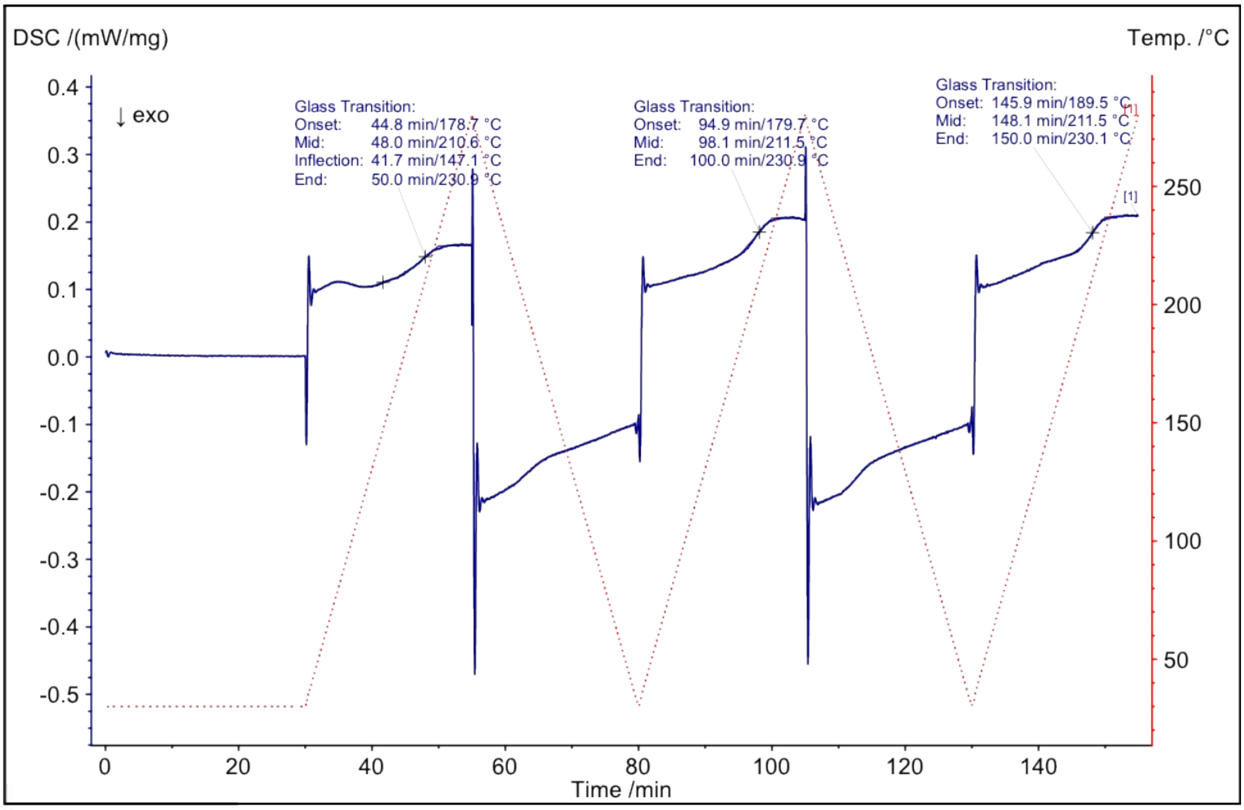

Figure S47. Full DSC up and down scans of the 1:3 blend using a heating and cooling rate of 10 °C min^{-1}. A third upscan was taken to confirm the reproducibility of the T_g.

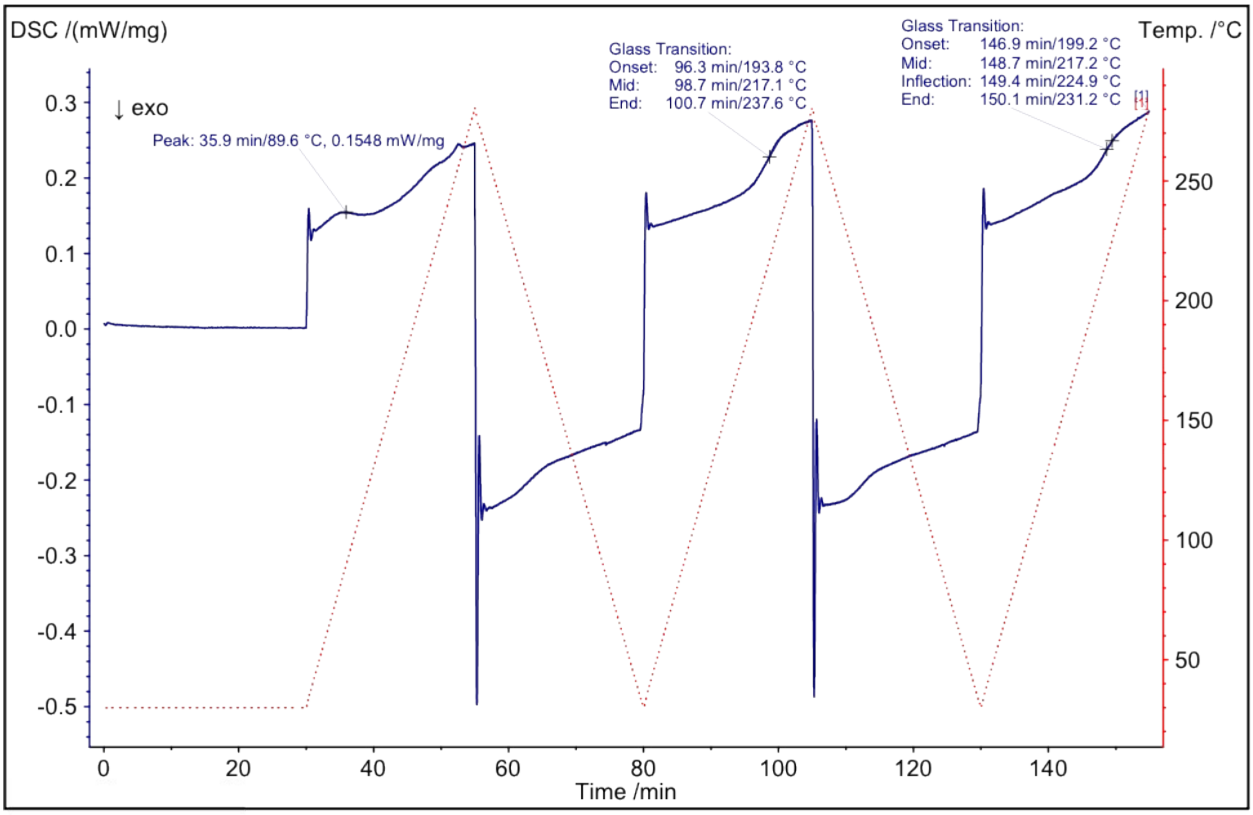

Figure S48. Full DSC up and down scans of the 1:6 blend using a heating and cooling rate of 10 °C min^{-1}. A third upscan was taken to confirm the reproducibility of the T_g.

3.8 PDF analysis

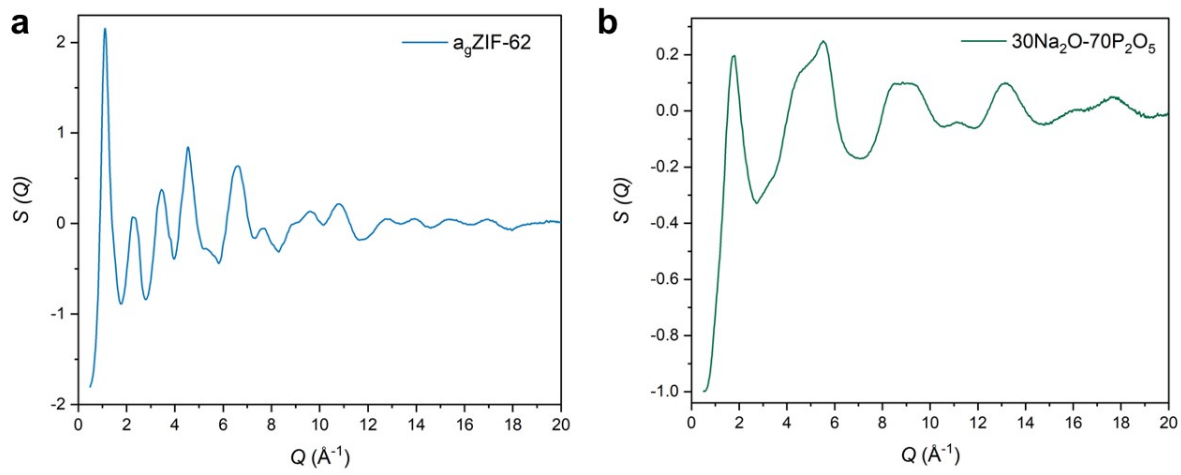

Figure S49. X-ray total scattering structure factor, *S*(*Q*), of pristine **a.** a_gZIF-62 and **b.** $30Na_2O-70P_2O_5$.

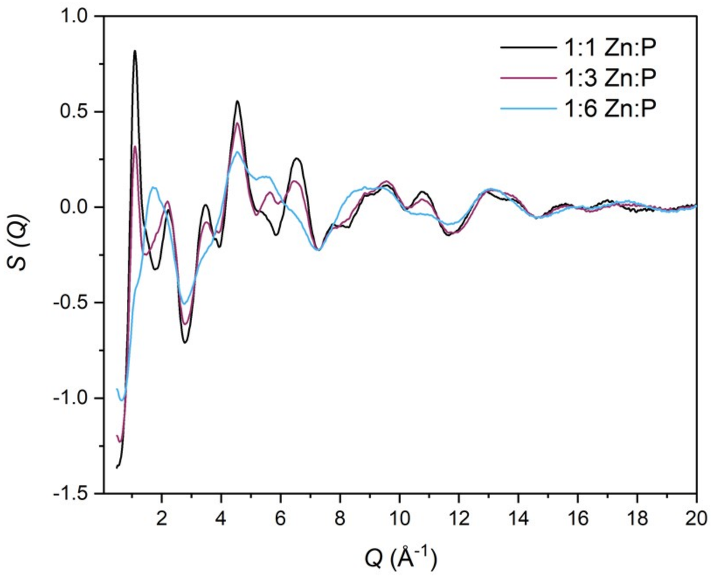

Figure S50. X-ray total scattering structure factor, *S*(*Q*), data of the three blend samples.

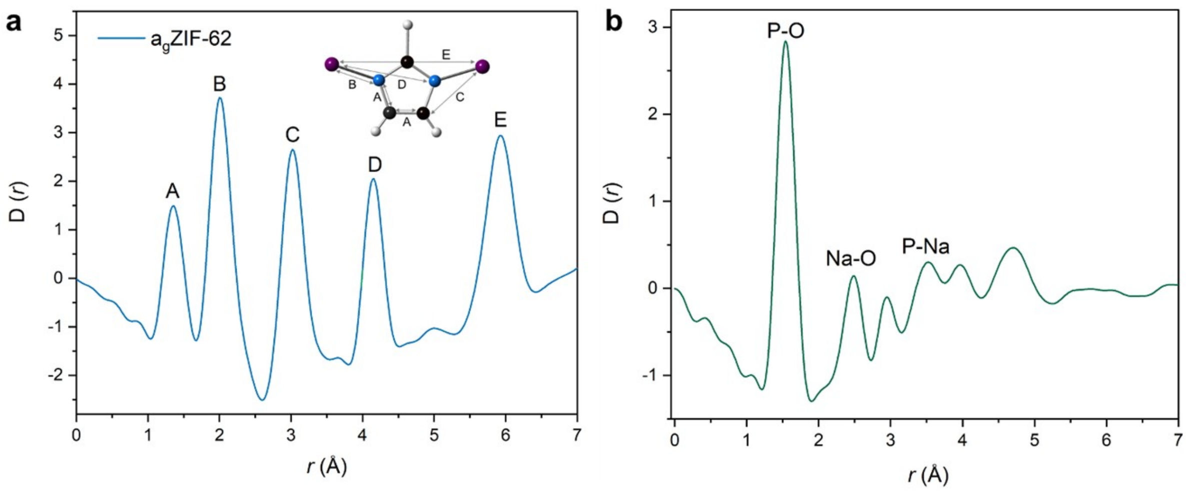

Figure S51. X-ray pair distribution function *D*(*r*) of pristine **a.** a_gZIF-62 and **b.** $30Na_2O-70P_2O_5$ with assigned correlations.

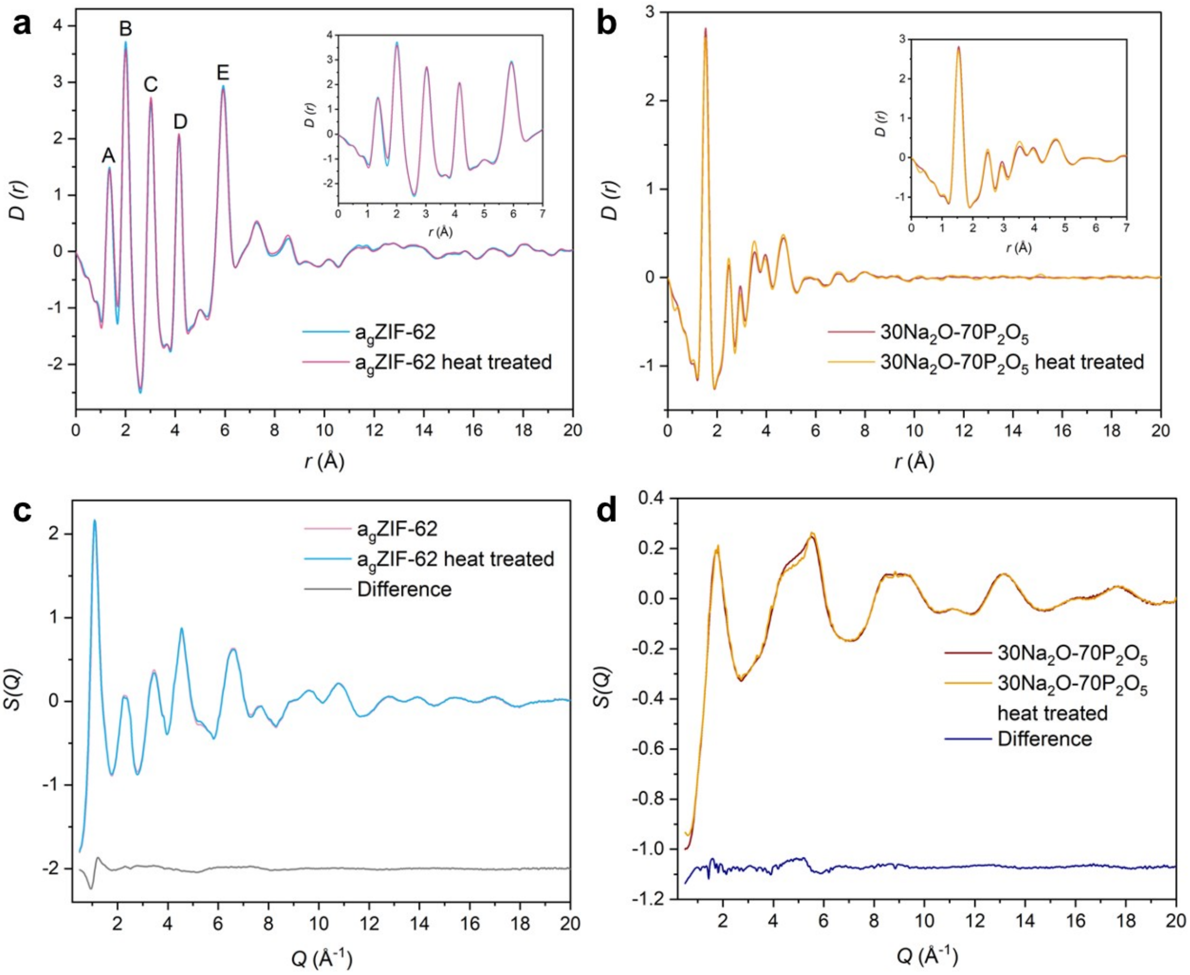

Figure S52. X-ray pair distribution function *D*(*r*) of heated treated vs pristine **a.** a_gZIF-62 and **b.** $30Na_2O$-$70P_2O_5$.

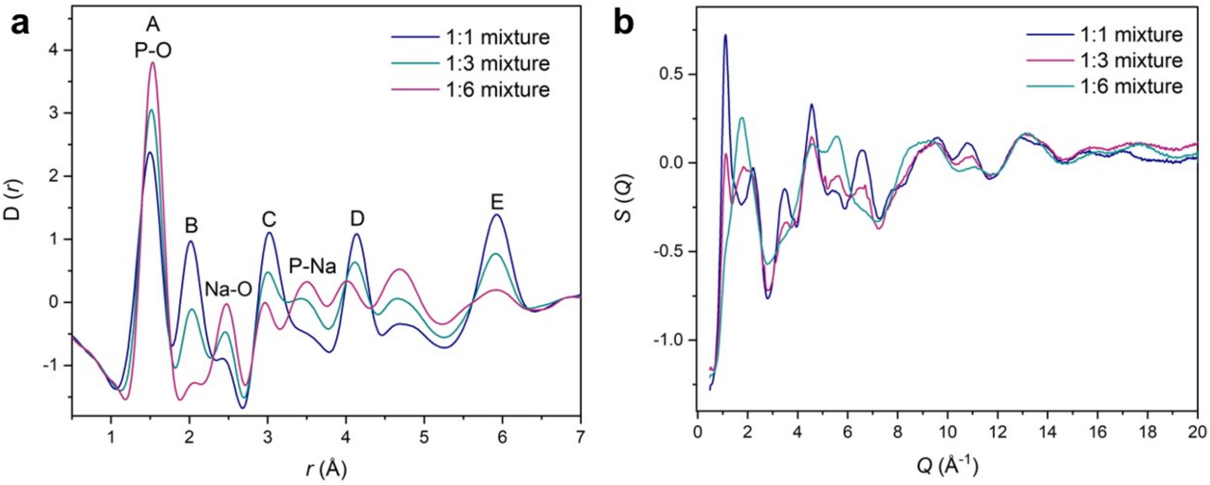

Figure S53. a. *D*(*r*) and **b.** Total scattering structure factor, *S*(*Q*), data of the three physical mixtures.

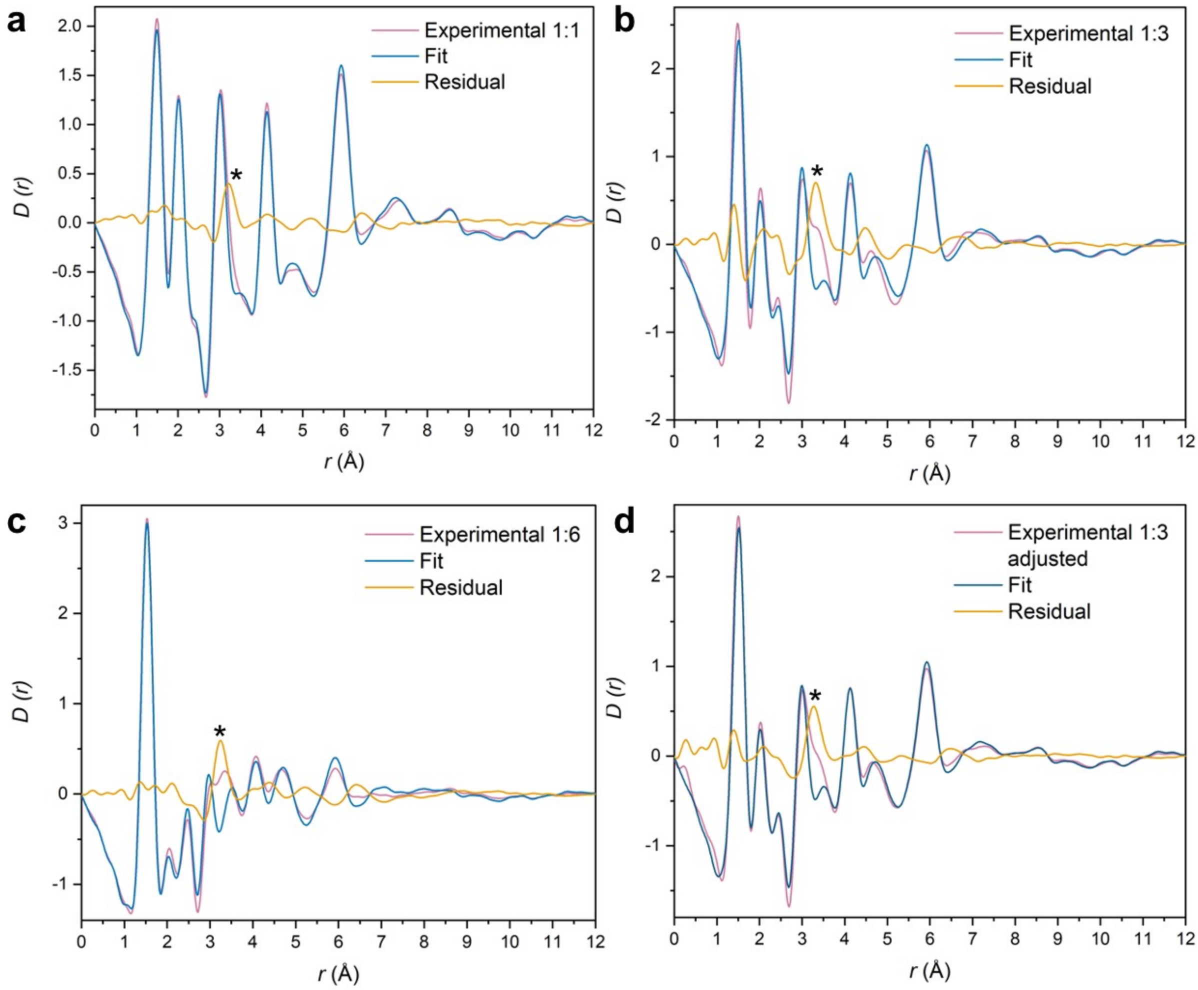

Figure S54. Experimental $D(r)$ plotted against the multiple linear regression fits of **a.** 1:1 blend, **b.** 1:3 blend, **c.** 1:6 blend and **d.** adjusted 1:3 blend. An asterisk denotes the potential interface peak in all four plots.

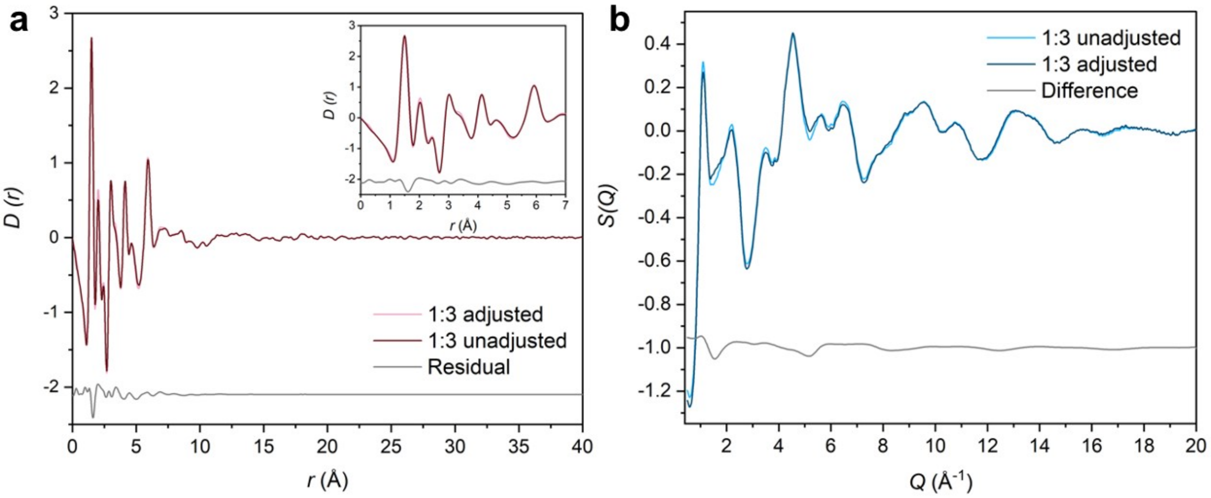

Figure S55. a. $D(r)$ comparison of the 1:3 adjusted and unadjusted sample, showing minor differences between the two $D(r)$ and **b.** Structure factor, $S(Q)$, comparison of the 1:3 adjusted and unadjusted sample.

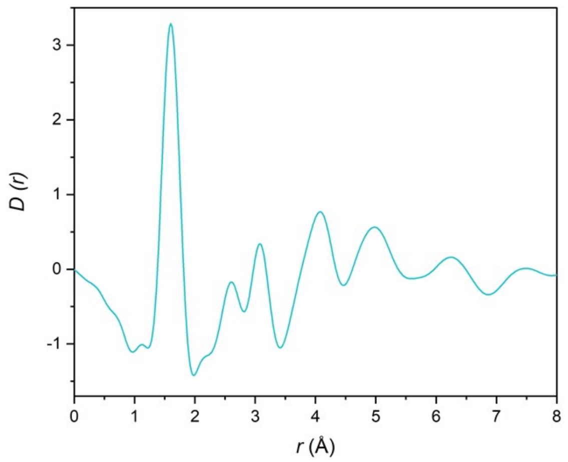

Figure S56. $D(r)$ of the empty borosilicate capillary used for data correction, showing a large peak at r = 1.6 Å.

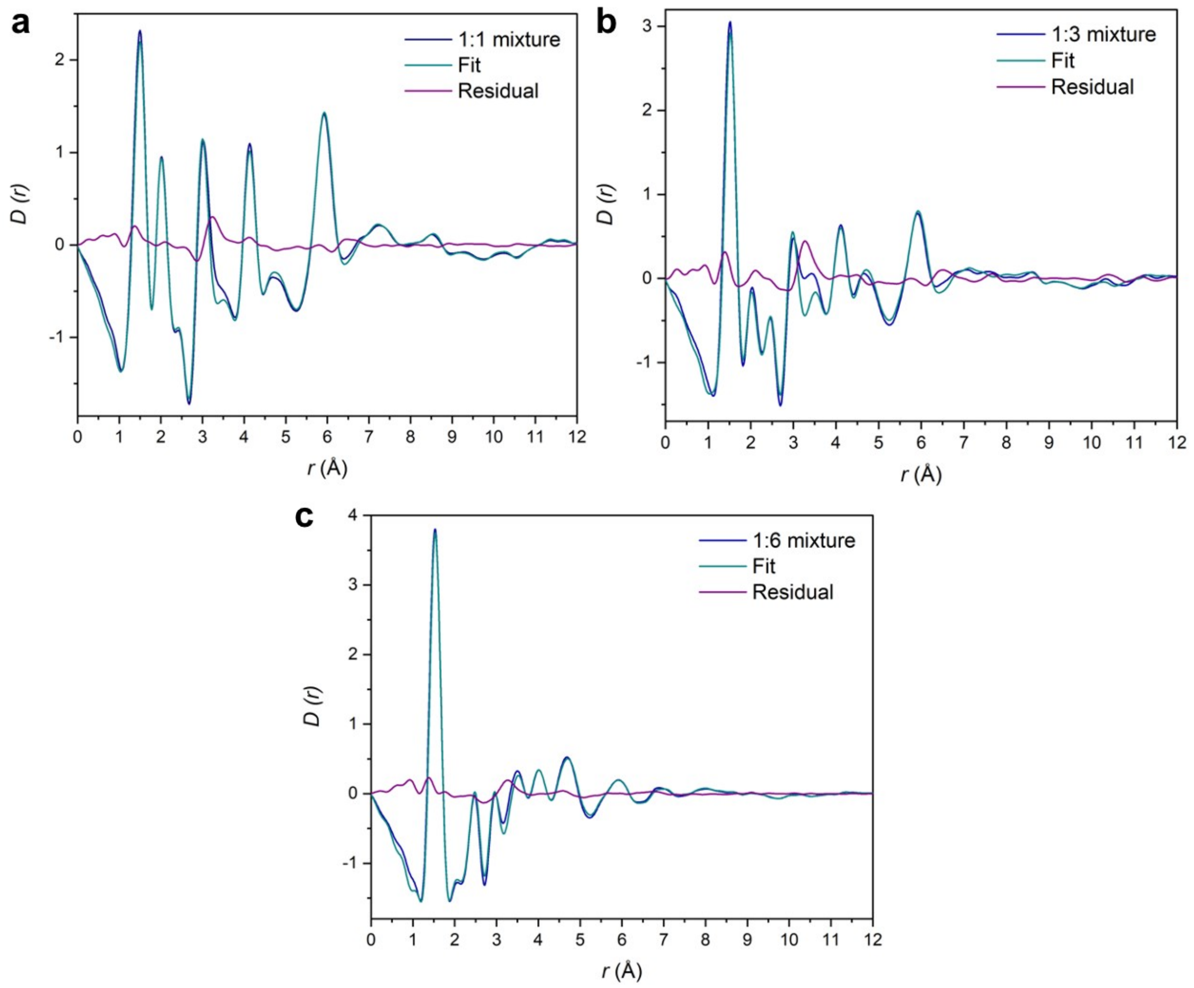

Figure S57. Experimental $D(r)$ plotted against the multiple linear regression fits of **a.** 1:1 physical mixture, **b.** 1:3 physical mixture and **c.** 1:6 physical mixture.

Table S2. R^2 values from the two-component multiple linear regression fits.

Sample	R^2 value	Reduced χ^2	Residual peak (Å)
1:1 physical mixture	0.988	0.00105	3.24
1:3 physical mixture	0.972	0.00211	3.28
1:6 physical mixture	0.992	0.00079	3.27
1:1 blend	0.985	0.00144	3.22
1:3 blend	0.932	0.00513	3.33
1:3 adjusted blend	0.964	0.0027	3.27
1:6 blend	0.963	0.0026	3.25

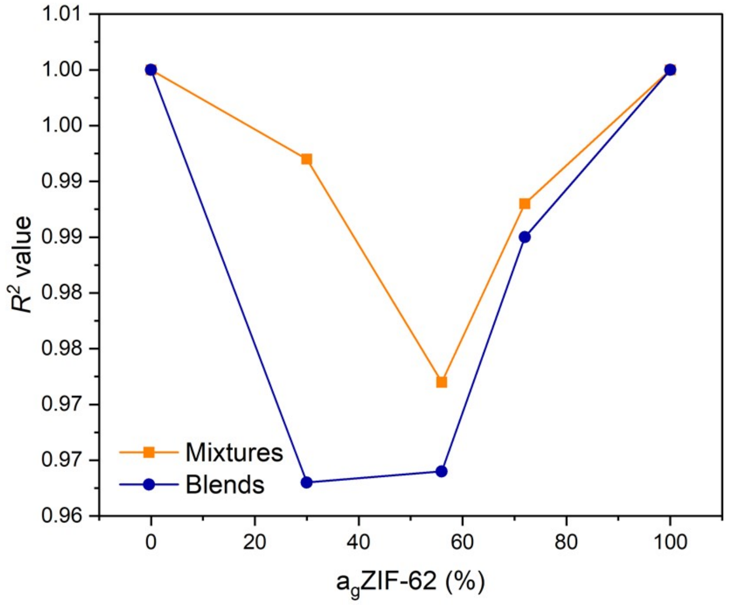

Figure S58. Goodness of fit R^2 values obtained from the multiple linear regression fits for the physical mixtures and blends. The % of $a_gZIF-62$ (x-axis) corresponds to the weight % of $a_gZIF-62$ in the blends.

Table S3. C_1 and C_2 values from the two-component multiple linear regression fits.

Sample	C_1 value	C_2 value
1:1 physical mixture	0.492 $\pm$ 0.001	0.780 $\pm$ 0.003
1:3 physical mixture	0.280 $\pm$ 0.002	1.051 $\pm$ 0.004
1:6 physical mixture	0.074 $\pm$ 0.001	1.337 $\pm$ 0.002
1:1 blend	0.550 $\pm$ 0.001	0.685 $\pm$ 0.003
1:3 blend	0.392 $\pm$ 0.003	0.836 $\pm$ 0.006
1:3 adjusted blend	0.363 $\pm$ 0.002	0.916 $\pm$ 0.004
1:6 blend	0.144 $\pm$ 0.002	1.077 $\pm$ 0.004

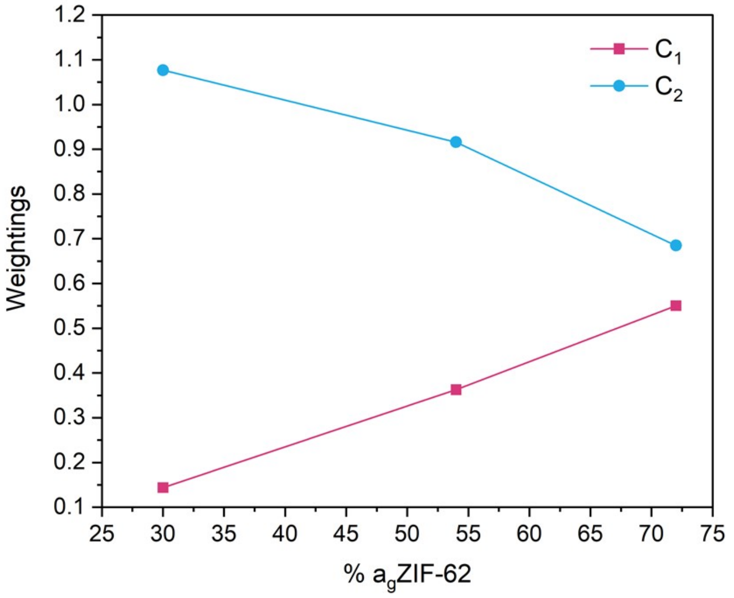

Figure S59. C_1 and C_2 across the compositional series with increasing % a_gZIF-62. C_1 and C_2 are linked to the amount of a_gZIF-62 and inorganic glass in the blends respectively. The % of a_gZIF-62 (x-axis) corresponds to the weight % of a_gZIF-62 in the blends.

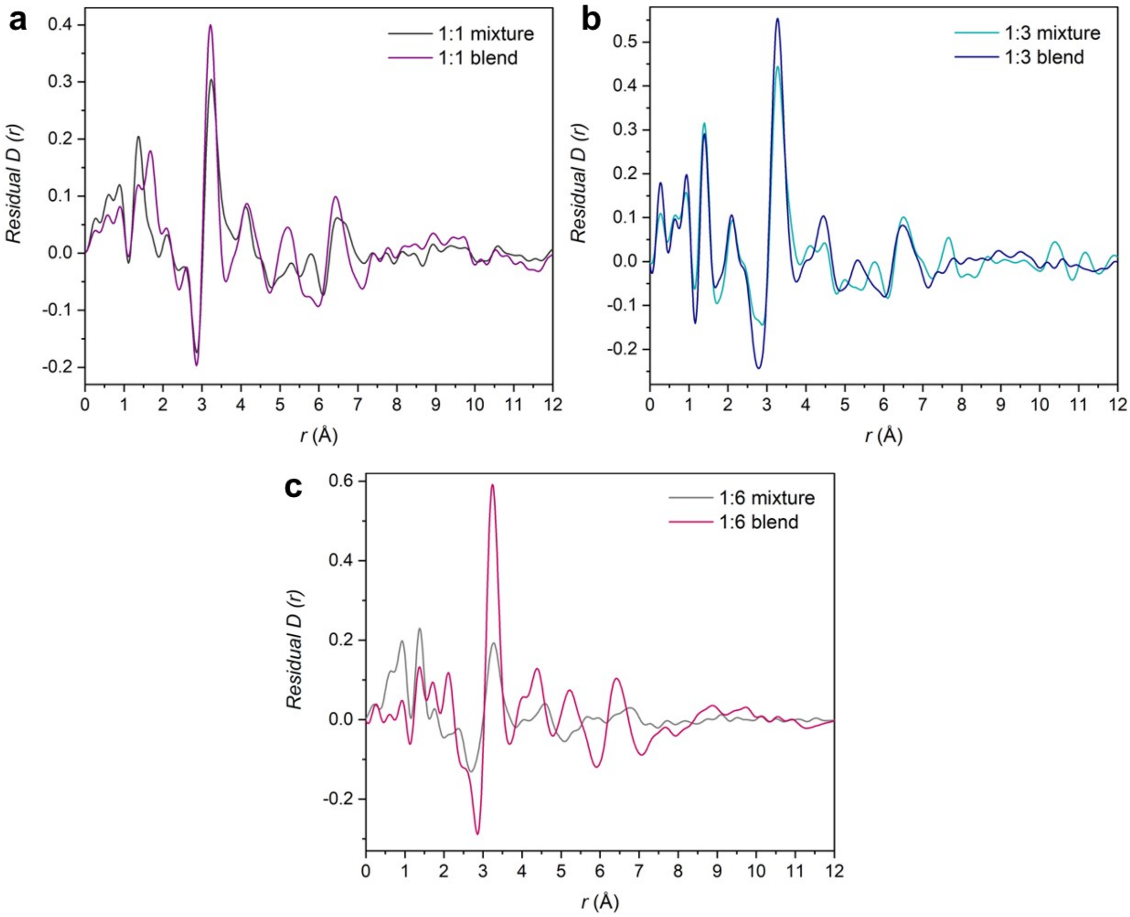

Figure S60. Residuals from the multiple linear regression fits of **a.** 1:1 physical mixture and blend, **b.** 1:3 physical mixture and blend, and **c.** 1:6 physical mixture and blend.

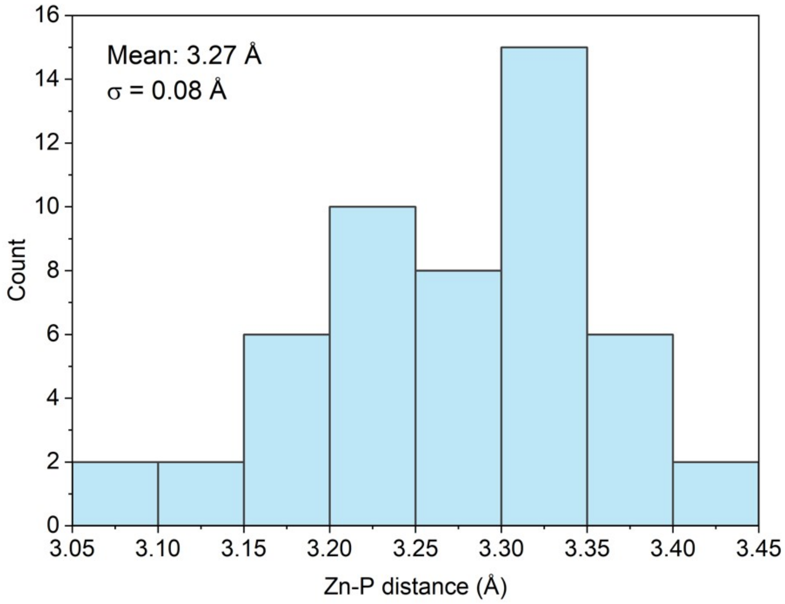

Figure S61. Average Zn-P distance via a bridging oxygen atom from three zinc phosphate CIFs, CCDC numbers 1007095, 2310787 and 2310789.

3.9 ^{31}P NMR spectroscopy

Table S4. Chemical shifts obtained from the Gaussian fitting of Direct ^{31}P and CP ^{31}P {^{1}H} NMR data.

Sample	Adjusted R^2 (^{31}P)	Peak chemical shift (ppm) ^{31}P	Adjusted R^2 (^{31}P{^{1}H})	Peak chemical shift (ppm) ^{31}P{^{1}H}
$30Na_2O$-$70P_2O_5$ (IG)	0.980	-38.5<br>-26.4<br>-10.7<br>-6.5<br>0.2	0.995	-37.8<br>-26.1<br>-10.7<br>-8.8<br>-4.6<br>0.8<br>3.9
1:6 blend (70% IG)	0.998	-35.0<br>-24.0<br>-10.3	0.998	-35.0<br>-23.9<br>-10.4
1:3 blend (44% IG)	0.999	-35.0<br>-23.6<br>-9.5	0.997	-35.0<br>-23.5<br>-9.9
1:1 blend (28% IG)	0.997	-35.0<br>-23.6<br>-9.6	0.993	-35.0<br>-23.5<br>-10.1

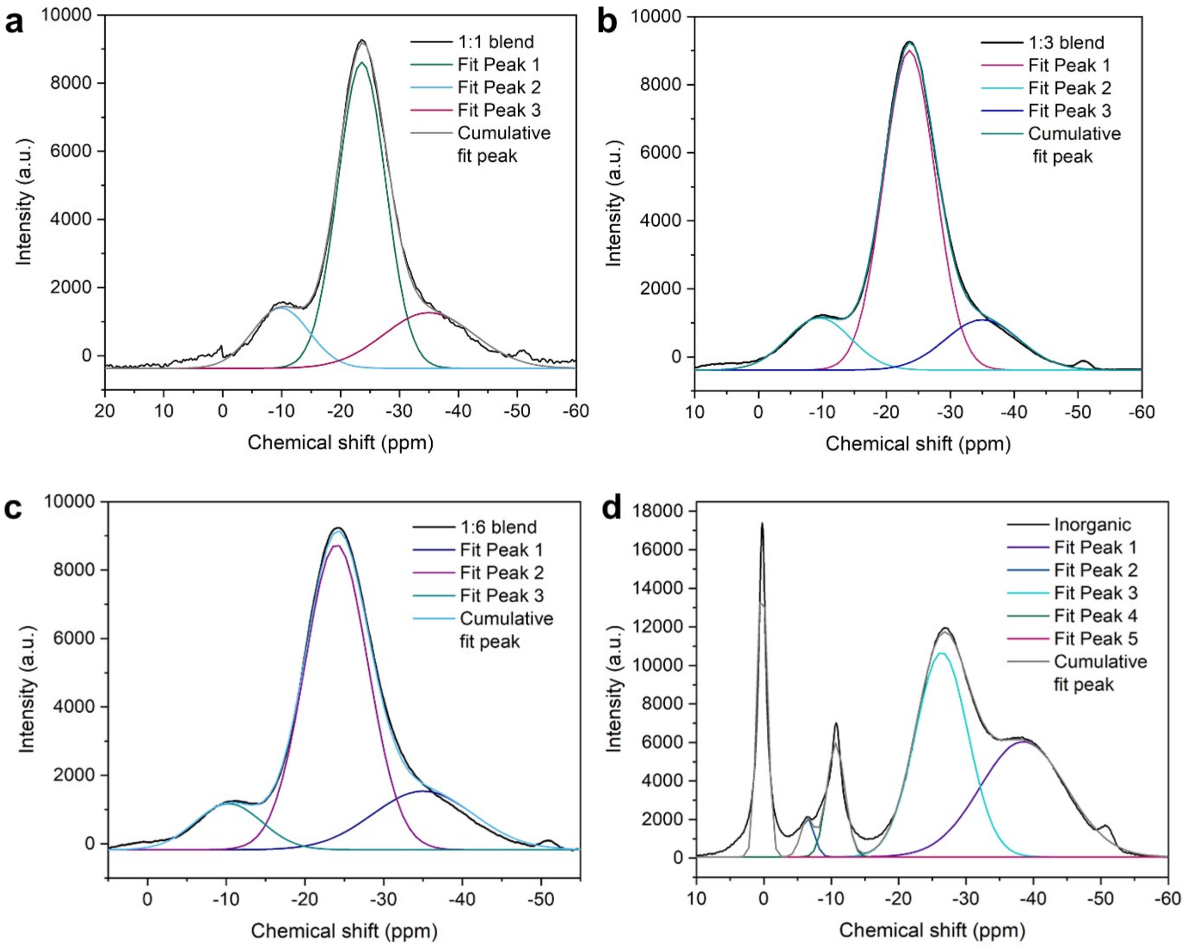

Figure S62. Gaussian fitting of the ^{31}P NMR data for **a.** 1:1 blend, **b.** 1:3 blend, **c.** 1:6 blend and **d.** $30Na_2O$-$70P_2O_5$ inorganic glass.

Table S5. Peak intensity changes in ^{31}P NMR vs ^{31}P $\{^1H\}$ NMR data.

Sample	Intensity of peak at ± -10 ppm	Intensity of peak at ± -35 ppm	Intensity ratio
1:1 blend ^{31}P	1766.0	1637.0	1.08
1:1 blend ^{31}P $\{^1H\}$	4752.9	1228.4	3.87
1:3 blend ^{31}P	1532.0	1476.2	1.04
1:3 blend ^{31}P $\{^1H\}$	3801.1	912.6	4.17
1:6 blend ^{31}P	1347.5	1715.0	0.79
1:6 blend ^{31}P $\{^1H\}$	2766.2	895.0	3.09

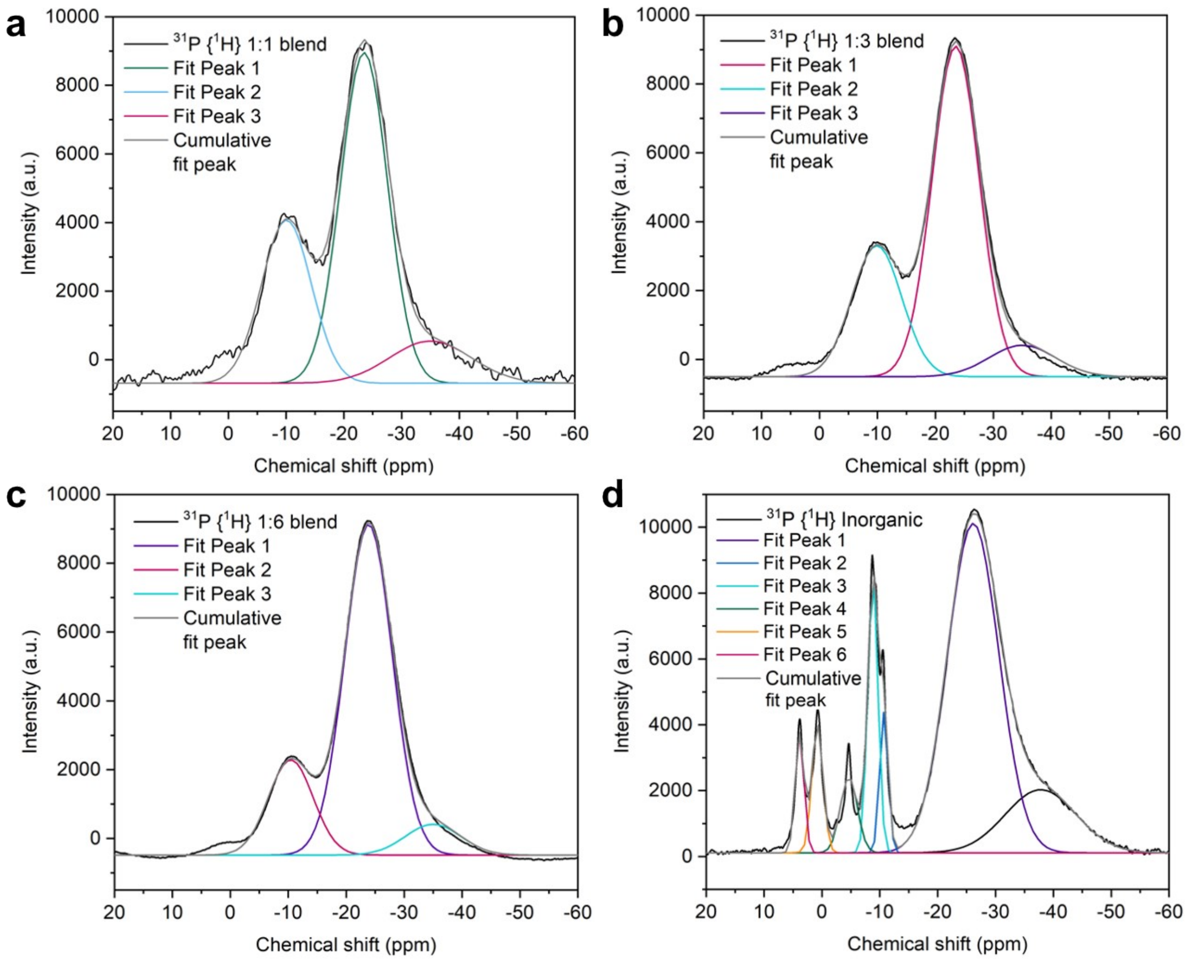

Figure S63. Gaussian fitting of the ^{31}P {^{1}H} NMR data for **a.** 1:1 blend, **b.** 1:3 blend, **c.** 1:6 blend and **d.** $30Na_2O$-$70P_2O_5$ inorganic glass.

The difference in the peaks observed on the same pure inorganic glass sample on direct and CP spectra can be attributed to different intensity gains because of cross-polarisation phenomena in the P-OH groups in the glass. These intensity gains are caused by hydrolysis of the glass network from air exposure prior to sample measurement. Their absence in the blends' ^{31}P {^{1}H} MAS NMR spectra suggests that hybridisation with a_gZIF-62 stabilises the glass against this hydrolysis processes. This is also evident in the PXRD analysis, where the inorganic control displays Bragg peaks corresponding to recrystallisation after ball milling, pelletisation and heating. These are absent in the blend PXRDs. Such evolution of pure inorganic glass sample (before and after heating treatment) is very dependent on the manipulation and storage conditions and therefore, it is not always reproducible. As a proof of this concept, a different batch of $30Na_2O$-$70P_2O_5$ and its heat-treated control (under the experimental conditions) was measured, in which lower degree of chemical evolution was observed (**Figure S64**). In this case, only a limited hydrolysis has been observed after heating treatment. This suggests that differences in the inorganic glass' direct and CP spectra arise from hydrolysis of the glass, instead of the heat treatment itself.

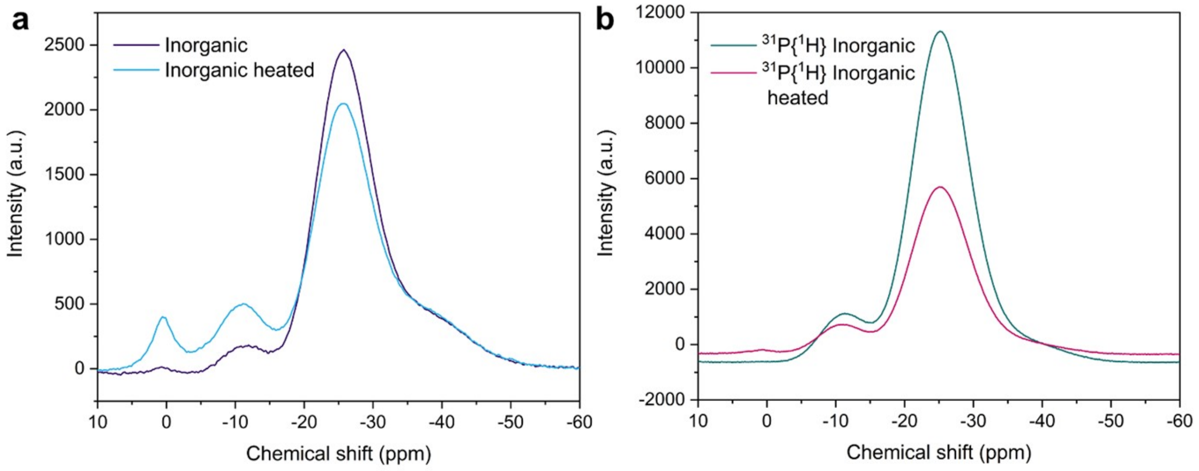

Figure S64. Comparison of second batch of inorganic glass and its heat treated control **a.** Direct ^{31}P and **b.** ^{31}P $\{^1H\}$ cross polarisation.

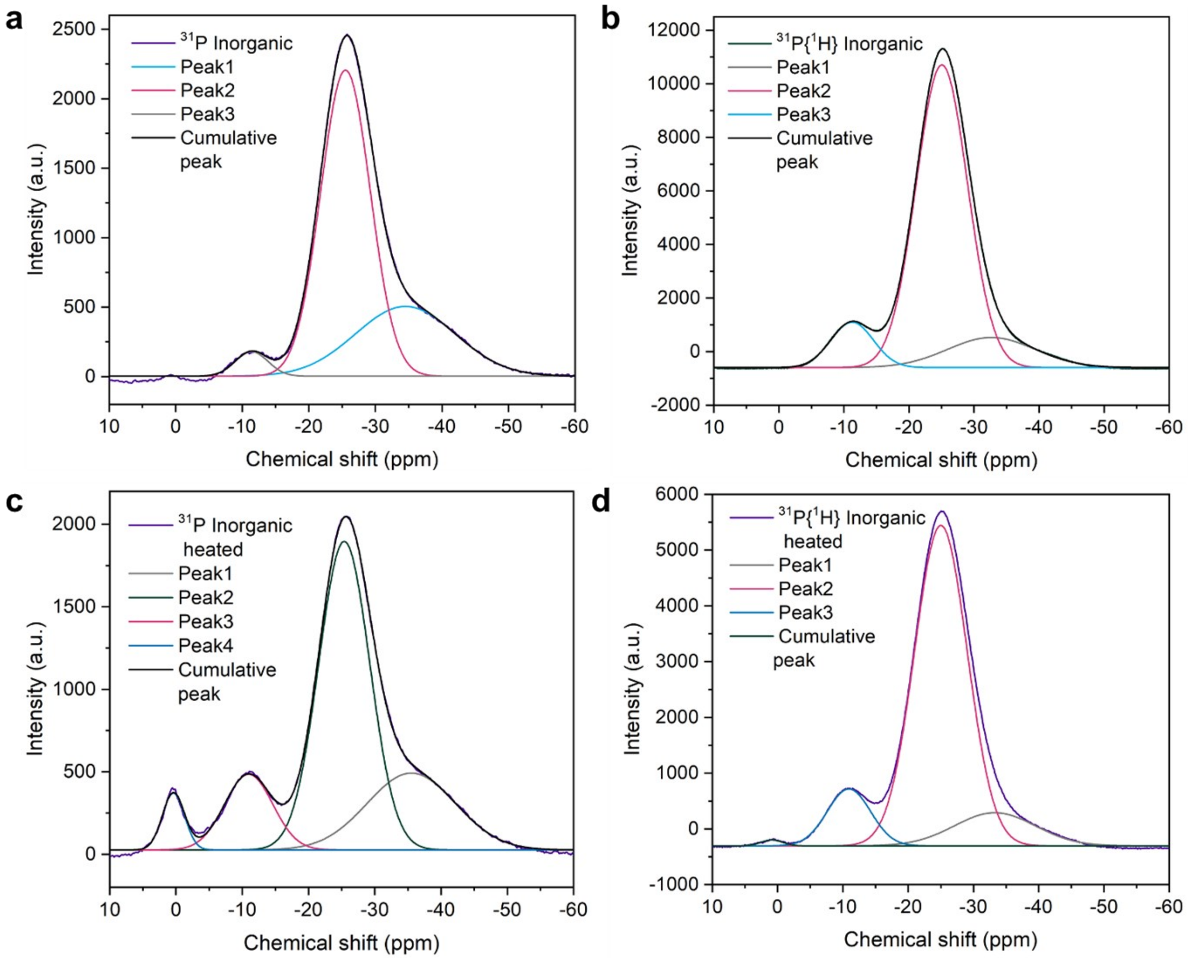

Figure S65. Gaussian fitting of the second batch of inorganic glass, **a.** Direct ^{31}P and **b.** ^{31}P $\{^1H\}$ cross polarisation and Gaussian fitting of the heat treated second batch of inorganic glass, **c.** Direct ^{31}P and **d.** ^{31}P $\{^1H\}$ cross polarisation.

Table S6. Chemical shifts obtained from the Gaussian fitting of Direct ^{31}P and CP $^{31}P\ \{^{1}H\}$ NMR data of the second batch of inorganic glass and its heat-treated control.

**Sample**	**Adjusted R^2 (^{31}P)**	**Peak chemical shift (ppm) ^{31}P**	**Adjusted R^2 ($^{31}P\{^{1}H\}$)**	**Peak chemical shift (ppm) $^{31}P\{^{1}H\}$**
Second batch of inorganic glass	0.998	-34.5<br>-25.5<br>-11.4	0.9997	-32.7<br>-25.1<br>-11.3
Heat treated second batch of inorganic glass	0.998	-35.5<br>-25.4<br>-11.0<br>0.4	0.9996	-33.3<br>-25.0<br>-10.9<br>0.9

References

1. M. F. Thorne, M. L. R. Gómez, A. M. Bumstead, S. Li and T. D. Bennett, *Green Chem.*, 2020, **22**, 2505–2512.
2. M. F. Thorne, A. F. Sapnik, L. N. McHugh, A. M. Bumstead, C. Castillo-Blas, D. S. Keeble, M. Diaz Lopez, P. A. Chater, D. A. Keen and T. D. Bennett, *Chem. Commun.*, 2021, **57**, 9272–9275.
3. R. K. Brow, *J Non Cryst Solids*, 2000, **263–264**, 1–28.
4. N. H. Ray, *J. Polym. Sci. Polym. Chem. Ed.*, 1973, **11**, 2169–2177.
5. M. A. Ghauri, S. A. Siddiqi, W. A. Shah, M. G. B. Ashiq and M. Iqbal, *J Non Cryst Solids*, 2009, **355**, 2466–2471.
6. P. K. Jha, O. P. Pandey and K. Singh, *J. Mol. Struct.*, 2015, **1083**, 278–285.
7. S. Mohan, N. Sundaraganesan and J. Mink, *Spectrochimica Acta Part A: Molecular Spectroscopy*, 1991, **47**, 1111–1115.
8. M. Y. B. Zulkifli, Y. Yao, R. Chen, M. Chai, K. Su, X. Li, Y. Zhou, R. Lin, Z. Zhu, K. Liang, V. Chen and J. Hou, *Chem. Commun.*, 2022, **58**, 12297–12300.
9. J. J. Hudgens, R. K. Brow, D. R. Tallant and S. W. Martin, *J Non Cryst Solids*, 1998, **223**, 21–31.
10. F. De La Peña, E. Prestat, V. T. Fauske, P. Burdet, J. Lähnemann, P. Jokubauskas, T. Furnival, M. Nord, T. Ostasevicius, K. E. MacArthur, D. N. Johnstone, M. Sarahan, J. Taillon, T. Aarholt, Pquinn-Dls, V. Migunov, A. Eljarrat, J. Caron, C. Francis, T. Nemoto, T. Poon, S. Mazzucco, Actions-User, N. Tappy, N. Cautaerts, Suhas Somnath, T. Slater, M. Walls, F. Winkler and H. W. Ånes, hyperspy/hyperspy (version v1.7.3) Zenodo 2022.
11. J. Hou, P. Chen, A. Shukla, A. Krajnc, T. Wang, X. Li, R. Doasa, L. H. G. Tizei, B. Chan, D. N. Johnstone, R. Lin, T. U. Schülli, I. Martens, D. Appadoo, M. S. Ari, Z. Wang, T. Wei, S.-C. Lo, M. Lu, S. Li, E. B. Namdas, G. Mali, A. K. Cheetham, S. M. Collins, V. Chen, L. Wang and T. D. Bennett, *Science*, 2021, **374**, 621–625.
12. J. Böhning, T. A. M. Bharat and S. M. Collins, *Structure*, 2022, **30**, 408-417.e4.
13. W. van Aarle, W. J. Palenstijn, J. De Beenhouwer, T. Altantzis, S. Bals, K. J. Batenburg and J. Sijbers, *Ultramicroscopy*, 2015, **157**, 35–47.
14. T. Goldstein, M. Li, X. Yuan, E. Esser and R. Baraniuk, , DOI:10.48550/ARXIV.1305.0546.
15. P. A. Midgley, M. Weyland, J. M. Thomas and B. F. G. Johnson, *Chem. Commun.*, 2001, 907–908.
16. Y. Yuan, K. E. MacArthur, S. M. Collins, N. Brodusch, F. Voisard, R. E. Dunin-Borkowski and R. Gauvin, *Ultramicroscopy*, 2021, **220**, 113166.
17. S. M. Collins, K. E. MacArthur, L. Longley, R. Tovey, M. Benning, C.-B. Schönlieb, T. D. Bennett and P. A. Midgley, *APL Materials*, 2019, **7**, 091111.
18. D. Zanaga, T. Altantzis, L. Polavarapu, L. M. Liz-Marzán, B. Freitag and S. Bals, *Part. Part. Syst. Charact.*, 2016, **33**, 396–403.
19. D. Zanaga, T. Altantzis, J. Sanctorum, B. Freitag and S. Bals, *Ultramicroscopy*, 2016, **164**, 11–16.
